# 1,5-Disubstituted tetrazoles as PD-1/PD-L1 antagonists†

**DOI:** 10.1039/d3md00746d

**Published:** 2024-02-21

**Authors:** Robin van der Straat, Rosalie Draijer, Ewa Surmiak, Roberto Butera, Lennart Land, Katarzyna Magiera-Mularz, Bogdan Musielak, Jacek Plewka, Tad A. Holak, Alexander Dömling

**Affiliations:** a Department of Drug Design, University of Groningen 9713 AV Groningen The Netherlands; b Department of Organic Chemistry, Faculty of Chemistry, Jagiellonian University 30-387 Kraków Poland; c Institute of Molecular and Translational Medicine, Faculty of Medicine and Dentistry and Czech Advanced Technology and Research Institute, Palackȳ University in Olomouc Olomouc Czech Republic alexander.domling@upol.cz

## Abstract

The progress in cancer survival and treatment has witnessed a remarkable transformation through the innovative approach of targeting the inhibitory immune checkpoint protein PD-1/PD-L1 complex by mAbs, *e.g.* pembrolizumab (Keytruda). While generating 17.2 billion U.S. dollars in revenue in 2021, the true significance of these developments lies in their ability to enhance cancer patient outcomes. Despite the proven efficacy of mAbs in inhibiting the PD-1/PD-L1 signaling pathways, they face significant challenges, including limited response rates, high production costs, missing oral bioavailability, and extended half-lives that can lead to immune-related adverse effects. A promising alternative approach involves the use of small molecules acting as PD-1/PD-L1 antagonists to stimulate PD-L1 dimerization. However, the precise mechanisms of action of these molecules remain partially understood, posing challenges to their development. In this context, our research focuses on the creation of a novel scaffold based on the Ugi tetrazole four-component reaction (UT-4CR) to develop low-molecular-weight inhibitors of PD-L1. Employing structure-based methods, we synthesized a library of small compounds using biphenyl vinyl isocyanide, leading to the discovery of a structure–activity relationship among 1,5-disubstituted tetrazole-based inhibitors. Supported by a cocrystal structure with PD-L1, these inhibitors underwent biophysical testing, including HTRF and protein NMR experiments, resulting in the identification of potent candidates with sub-micromolar PD-L1 affinities. This finding opens opportunities to the further development of a new class of PD-L1 antagonists, holding promise for improved cancer immunotherapy strategies.

## Abstract 2

Utilizing a combination of structure-based design, MCR synthesis, biophysics, and protein crystallography to innovate a novel tetrazole scaffold targeting the PD-1/PD-L1 immune checkpoint protein complex.

## Graphical abstract

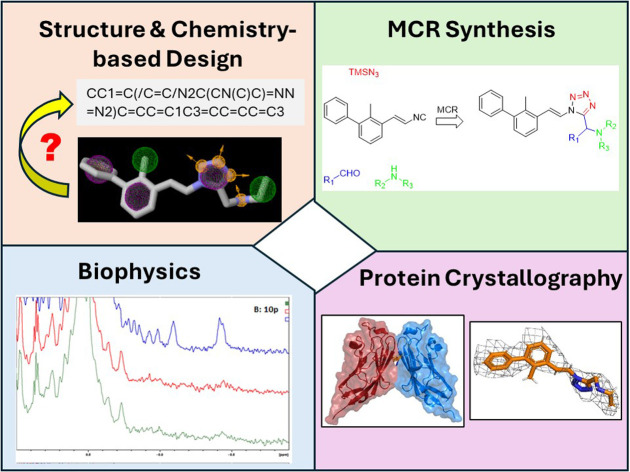

## 1. Introduction

The programmed death 1 (PD-1) receptor and its ligand PD-L1 have become an important target in cancer immunotherapy.^1^ Therapies based on the immune checkpoint PD-1/PDL-1 complex have significantly improved the clinical outcome of lethal cancers such as lymphoma, melanoma, and lung and breast cancers.^2^ Overexpression of membrane bound PD-L1 by tumor cells attenuates T-cell signaling^3^ to evade immune surveillance due to its binding with T-cell membrane bound PD-1. However, the immunosuppressive conditions can be reversed by blocking the interaction of PD-1 and PD-L1 and restoring the activity of the body's immune cells to kill tumor cells.^4^ Research in the last few decades has led to several monoclonal antibodies (mAbs) targeting PD-1 such as pembrolizumab,^5^ nivolumab^6^ and cemiplinab^7^ or monoclonal antibodies targeting PDL-1 like atezolizumab,^8^ avelumab^9^ and durvalumab.^10^ Despite the effectiveness of mAbs, they are by far not suitable for all patients, have high production costs, show adverse effects, and lack oral bioavailability, and resistance is observed.^11,12^ Alternatively, small molecule inhibitors antagonizing PD-1/PDL-1 are investigated. Since our first description of the human PD-1/PDL-1 crystal structure and our first cocrystal structure describing a small molecule in a PD-L1 dimer, a myriad of small molecules based on our pharmacophore model were described and a race began towards developing such compounds for clinical use.^13–21^ As part of our continuing research towards small molecules that antagonize PD-1/PDL-1, we present here the synthesis, biological activity, and structural basis of 1,5-disubstituted tetrazoles as PD-1/PD-L1 antagonists.

**Fig. 1:**
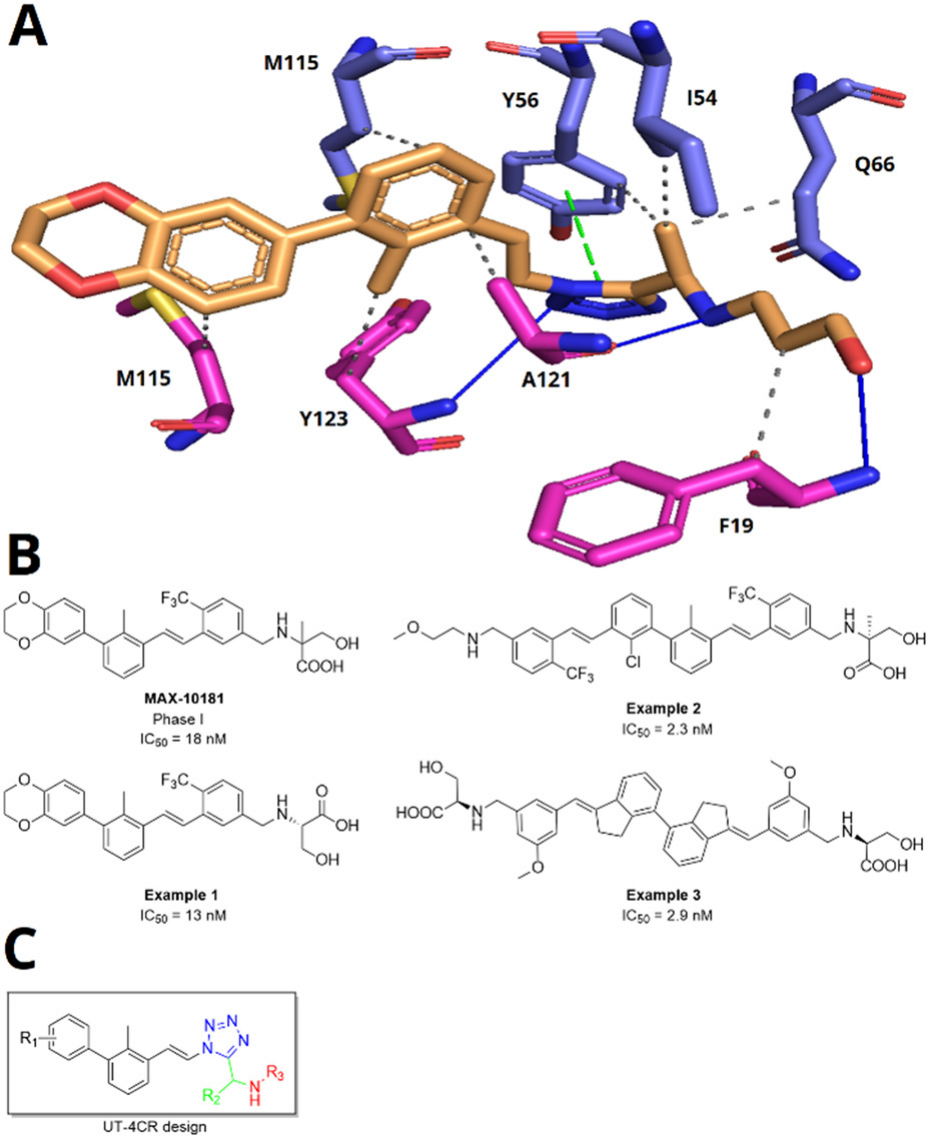
Small molecule PD-1/PD-L1 antagonist. (A) Modelling of a 1,5-disubstituted tetrazole into a PD-L1 dimer structure (PDB ID: 7NLD). (B) Examples of patented PD-L1 dimerizers by Maxinovel Pharma. (C) Design of 2,5-disubstituted tetrazoles by the UT-4CR. The colour code depicts the different fragments: the core biphenyl and linker isocyanide-derived motif in black, tetrazole in blue, the aldehyde-derived motif in green, and the primary amine-derived motif in red.

Based on the simplicity of execution, convergence, fast assembly, sustainability, structural diversity of, and our expertise in multi-component reaction (MCR) chemistry, we were asking if MCR scaffolds can be discovered to potently antagonizing the PD-1/PDL-1 interaction.^22,23^ Previously, we described 2-aminoimidazo pyridines as potent PD-1/PDL-1 antagonists which can be assembled by a convergent and versatile GBB-3CR MCR.^21^ Here, we explore if 1,5-disubstituted tetrazoles, which can be accessed in one step by the UT-4CR, can bind to PD-L1. Docking studies of hypothetical 1,5-disubstituted tetrazoles into the previously described PD-L1 small molecule crystal structure informed us about such possibility (Fig. 1A). For this, we designed different 1,5-disubstituted tetrazoles informed by our previously described pharmacophore model using MOLOC software.^21^ The core of these inhibitors is based upon the biphenyl moiety that interacts with the hydrophobic pocket by amino acids Met-115, Ala-121 and Tyr-123. The linker used plays an important role in managing the correct orientation of the aromatic and tail groups. Here, we designed an alkene linker similar to the compound in clinical trial Phase I MAX-10181 (Fig. 1B).^24^ The tetrazole, we hypothesize, can potentially interact due to its aromatic and *cis*-carboxamide-bioisosteric properties to facilitate the π–π interaction with Tyr-56 and hydrogen bond interaction with the Tyr-123 hydroxy group. Alkanolamines were selected to establish hydrogen bond interactions with Ala-121 and Phe-19 and to improve physical properties (Fig. 1A).

**Scheme 1:**
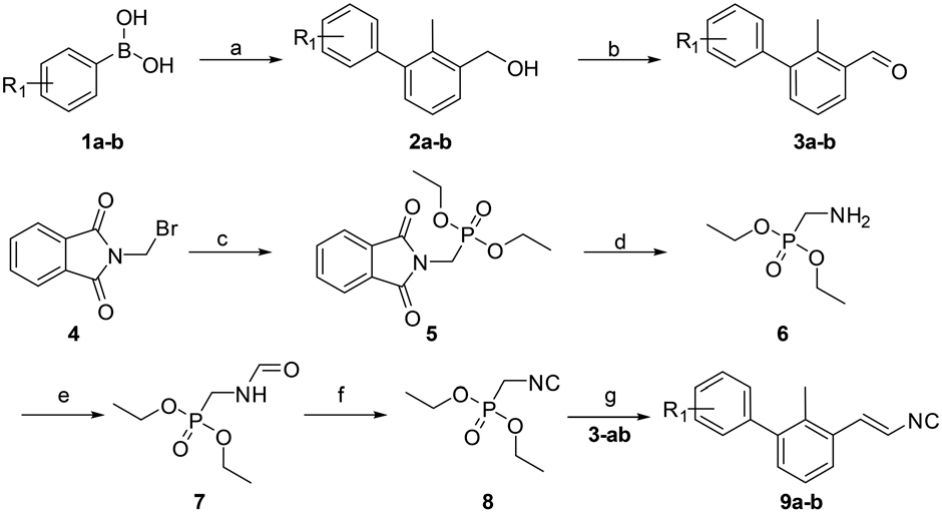
Synthetic route to compounds 9a and b. Reagents and conditions: (a) Pd(dppf)Cl_2_, toluene/ethanol/NaHCO_3_ (sat. aq.) (5 : 1 : 5) (0.3 M), 85 °C, 12 h; (b) PCC (2 eq.), DCM, rt, 3 h; (c) triethylphosphite (1.25 eq.), reflux, 4 h; (d) hydrazine hydrate (1.2 eq.), EtOH (0.25 M), rt to reflux, 4 h; (e) ethyl formate, reflux, 12 h; (f) phosphorus oxychloride (1.05 eq.), Et_3_N (5 eq.), DCM (1 M), −20 °C to rt, 3 h; (g) 1) LDA (1.2 eq.), THF (1.0 M), −78 °C to −20 °C, 30 min, 2) 3a and b (1.0 eq.), −20 °C to rt, 12 h. R^1^ = H, [3,4]-(OC_2_H_4_O).

Often PD-1/PD-L1 antagonists are synthesized by lengthy and linear sequential synthesis pathways. To avoid such a time-consuming process, we implemented the power of MCRs for the one-pot approach towards 1,5-disubstituted tetrazoles. For this, we used the Ugi tetrazole reaction (UT-4CR) which is a four component reaction of amines, aldehydes and isocyanides in the presence of an azide source, *e.g.* TMSN_3_ or NaN_3_, to grant access to drug-like molecules (Fig. 1C).^25^ In our design, the biphenyl moiety is the core of the scaffold, which is introduced by the use of (*E*/*Z*)-3-(2-isocyanovinyl)-2-methyl-1,1′-biphenyl 9a and b (Scheme 1). Additionally, three suitable substituents are attached to the core, namely, tetrazole (Fig. 1C, blue), aldehyde (Fig. 1C, green) and amine (Fig. 1C, red) moieties.

## 2. Results and discussion

### 2.1 Chemistry

A viable and scalable synthesis of biphenyl substituted vinyl isocyanide was investigated first (Scheme 1). The twisted biphenyl moiety was synthesized *via* the Suzuki cross-coupling reaction between (3-bromo-methylphenyl)-methanol and boronic acids 1a and b as described earlier by our group.^21^ Following the Suzuki reaction, we oxidized the alcohols 2a and b to the corresponding aldehydes using two equivalents of pyridinium chlorochromate in excellent yields of 90–94%. Diethyl isocyanomethylphosphonate was synthesized as described previously.^26^ Consequently, a modified Wittig reaction was used to obtain isocyanides 9a and b.^27,28^

**Scheme 2:**
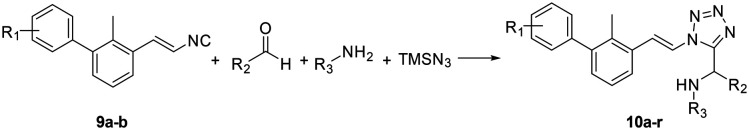
UT-4CR reaction. Reagents and conditions: Sc(OTf)_3_ (5 mol%), DCM/MeOH (1 : 1) (1.0 M), 8–12 h, r.t.

Having in hand multi-gram amounts of the desired isocyanides, we synthesized a small library of compounds based on the Ugi tetrazole reaction (UT-4CR, Scheme 2). Based on the modeling studies, we selected aliphatic amines containing a hydroxy group with different chain lengths for potential hydrogen bond interactions of the UT-4CR product –OH and –NH groups with the PD-L1 amino acid residues. As an aldehyde component, we selected paraformaldehyde or acetaldehyde. In the case of acetaldehyde, this should favor van der Waals interactions with the Ile-54 and Tyr-56 in the pocket of the PD-L1 dimer. Trimethylsilylazide was chosen as an azide source to allow the tetrazole formation for generating π stacking with Tyr-123.

The reaction conditions used for the UT-4CR are scandium triflate (5 mol%) as the catalyst and 1 : 1 DCM/MeOH as the solvent system at a concentration of 1 M with regard to the other starting materials which were used in an equimolar ratio. Stirring at room temperature for 8–12 hours generated the corresponding UT-4CR products in acceptable yields (18–57%). Subsequently, the target compounds were purified over silica using flash chromatography. The final compounds 10a–r (Table 1) were obtained and were analyzed *via* high-resolution mass spectrometry and ^1^H and ^13^C NMR spectroscopy (see the ESI†).

**Table 1:** SAR toward PD-L1 based on the HTRF methodology

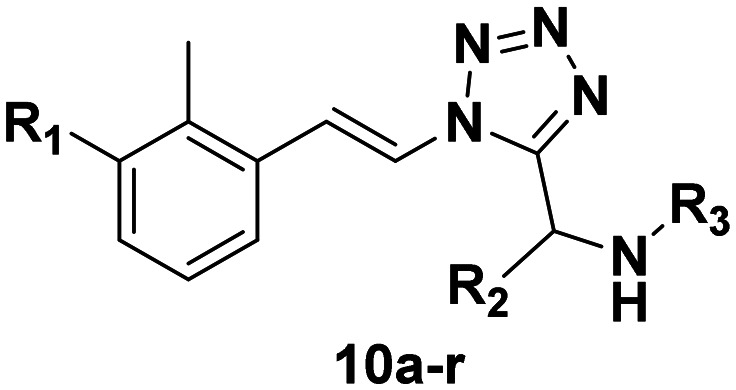				
Cp	R^1^	R^2^	R^3^	IC_50_ (nM)
10a	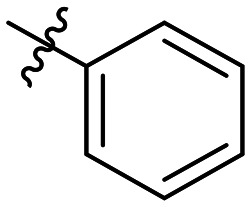	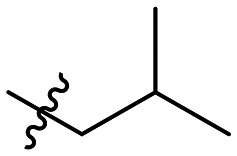	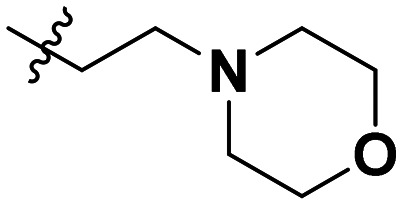	Inactive
10b	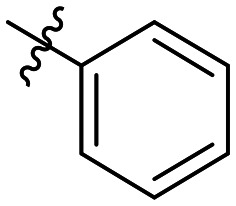	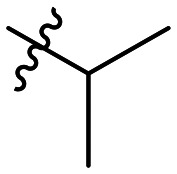	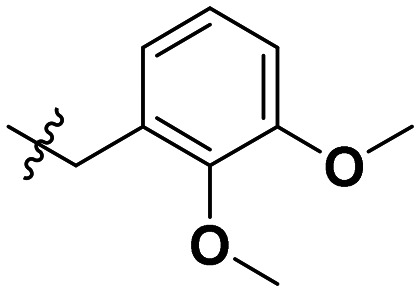	Inactive
10c^a^	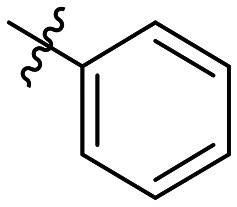	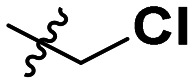	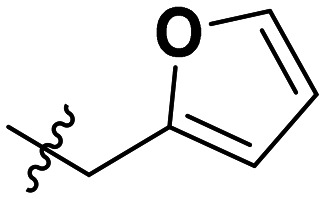	Inactive
10d^a^	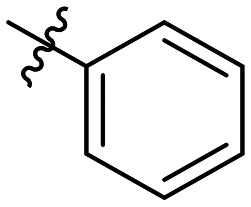	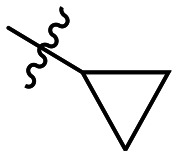	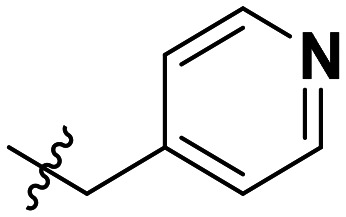	1770
10e	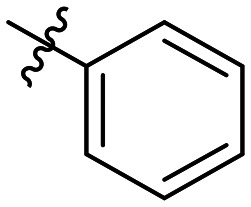	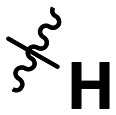	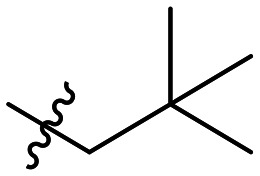	Inactive
10f	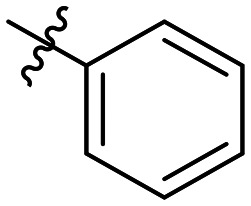	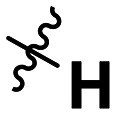	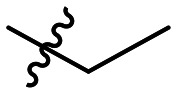	408
10g^a^	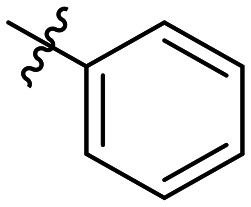	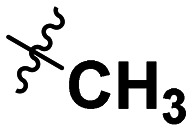	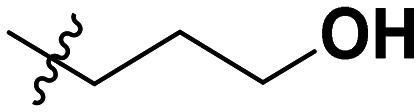	1800
10h	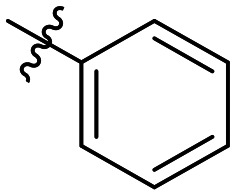	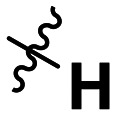	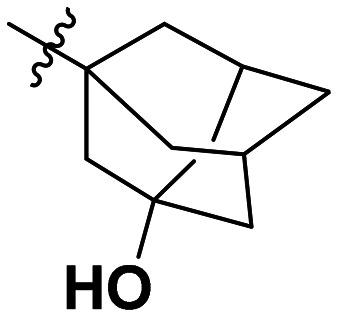	Inactive
10i	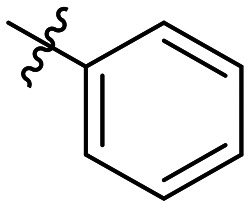	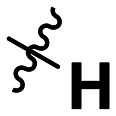	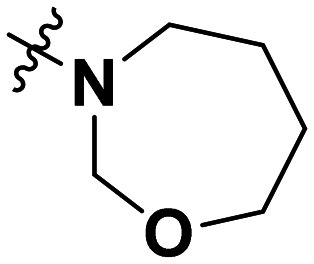	460
10j	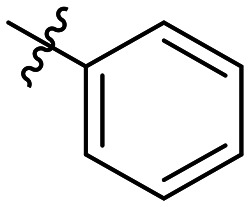	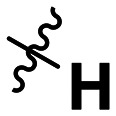	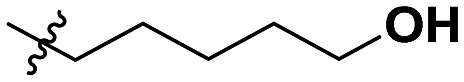	1840
10k	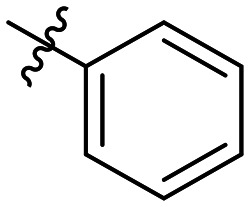	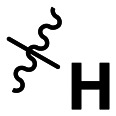	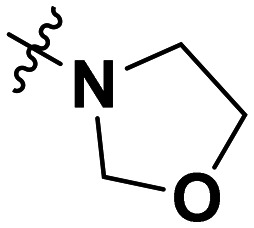	1000
10l	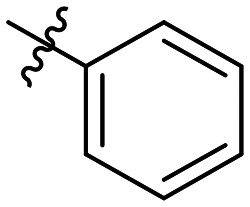	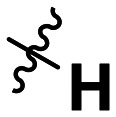	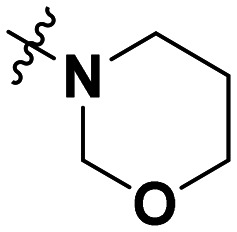	944
10m	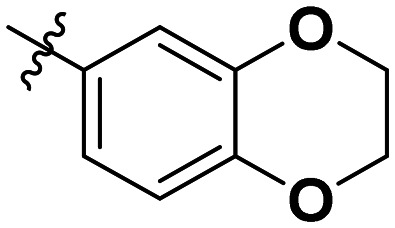	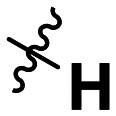	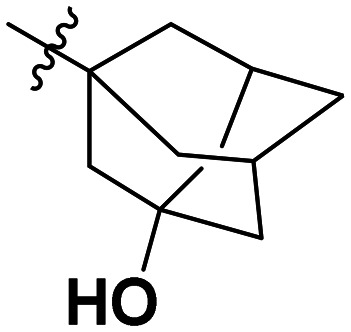	Inactive
10n	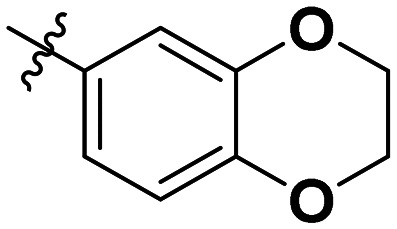	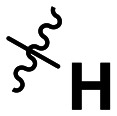	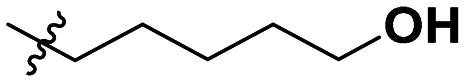	2850
10o	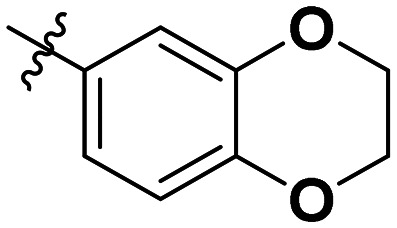	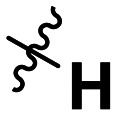	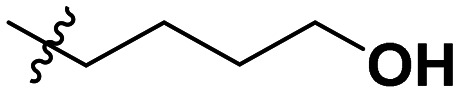	1130
10p	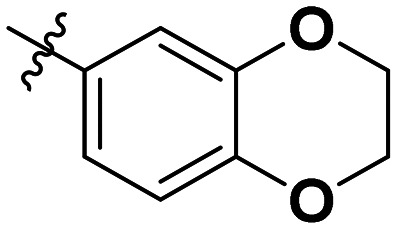	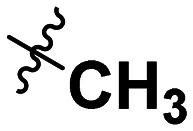	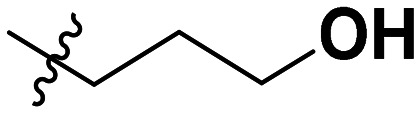	254
10q^a^	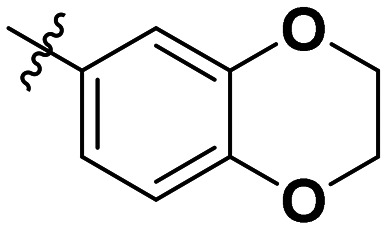	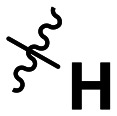	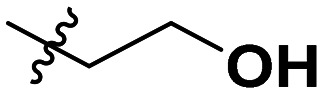	Inactive
10r	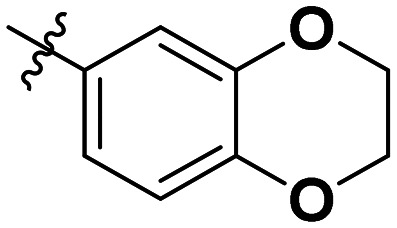	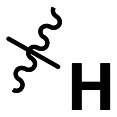	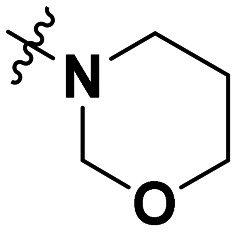	1130

a Racemic mixture.

### 2.2 Homogeneous time-resolved fluorescence

The synthesized compounds were tested toward binding to PD-L1 by the HTRF (homogeneous time-resolved fluorescence) assay in a one-point experiment, validating the percentage of dissociation of the PD-1/PD-L1 complex at 50 and 5 nM concentrations of each inhibitor (Table 1). Under these conditions, the BMS-1166 compound, used herein as a reference, gave the value of 42.1 ± 6.4% of undissociated complex. The IC_50_ of BMS-1166 in the HTRF assay was reported previously, with a value of 3.89 ± 0.19 nM.^29^ The majority of the tested compounds show slight activity towards dissociating the PD-1/PDL-1 complex. Several of the obtained compounds show better activity toward PD-L1 which included compounds 10f, 10l, and 10p. For compounds 10f, 10l and 10p, an IC_50_ determination was performed (Fig. S1–S3†). Moreover, the estimation of the IC_50_ was executed for all the synthesized compounds which allows us to validate the structure–activity relationship studies. Introducing an aliphatic chain in the α-position to the tetrazole ring (*e.g.*10a, 10c) larger than a methyl group (10g, 10p) was deleterious. No aliphatic side chain but hydrogen at the α-position led to active compound 10f. Likewise, introducing bulky amino alcohols, hydroxy adamantyl amine or a bulky aliphatic chain (*e.g.*10e, 10h) in contrast to the ring structure (*e.g.*10i) or the aliphatic alcohol (10j) was deleterious. The exchange of distal phenyl to benzo-14-dioxane results in a variable activity change comparing compounds 10g and 10p and compounds 10j and 10n. These differences indicate that the carbinolamine's chain length influences how the R^1^ subgroup binds.

### 2.3 NMR titration

**Fig. 2:**
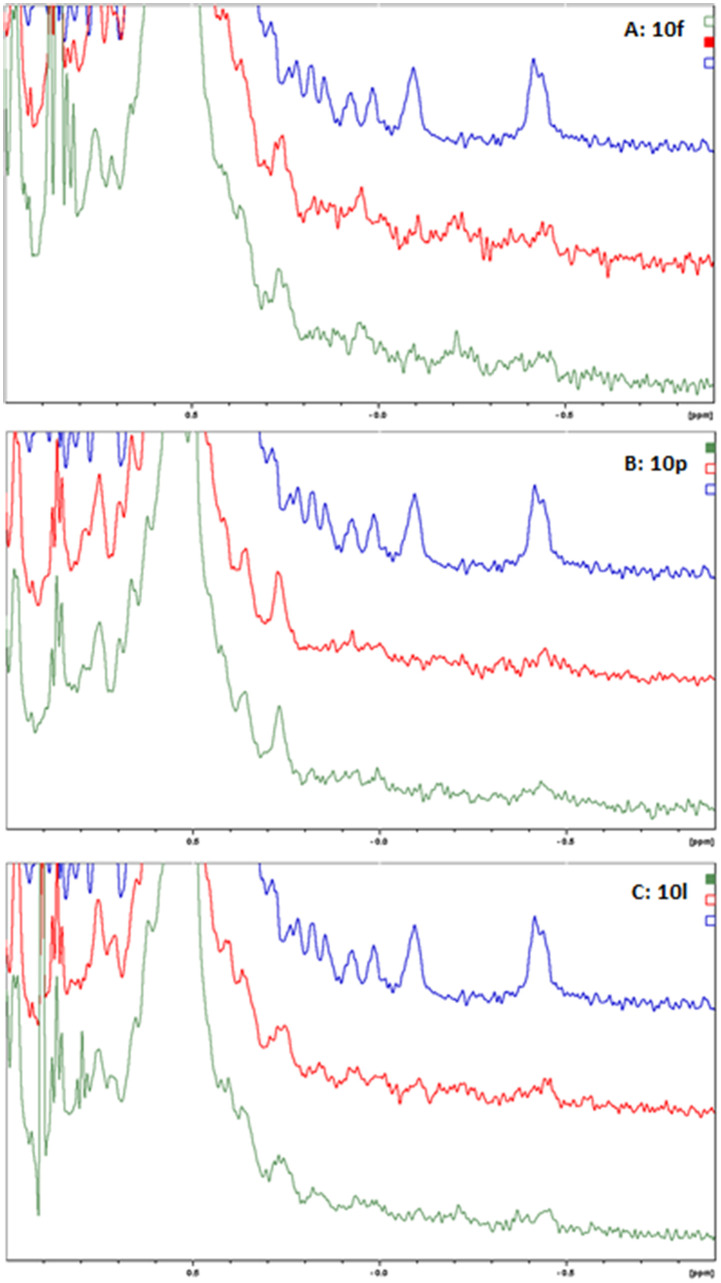
Aliphatic part of the ^1^H NMR spectra of PD-L1 with tetrazole-based inhibitors. ^1^H NMR spectra of hPD-L1 protein (blue), PD-L1 and compounds: (A) 10f, (B) 10p, and (C) 10l in protein to inhibitor molar ratios of 1 : 1 (red) and 1 : 10 (green), respectively.

To confirm the dimerization of the PD-L1 complex, molecules 10f, 10l and 10p were tested for the interaction with human PD-L1(hPD-L1) using a ^1^H NMR titration experiment and showed protein oligomerization upon addition of the ligand at the molar ratio of protein to inhibitor equal to 1 : 1 (Fig. 2), which is indicated as broadening of the hPD-L1 proton signals in the spectra. The corresponding behaviour is characteristic of hPD-L1 oligomerization, previously observed by our group with BMS reference compounds.^20^

### 2.4 X-ray crystallography

**Fig. 3:**
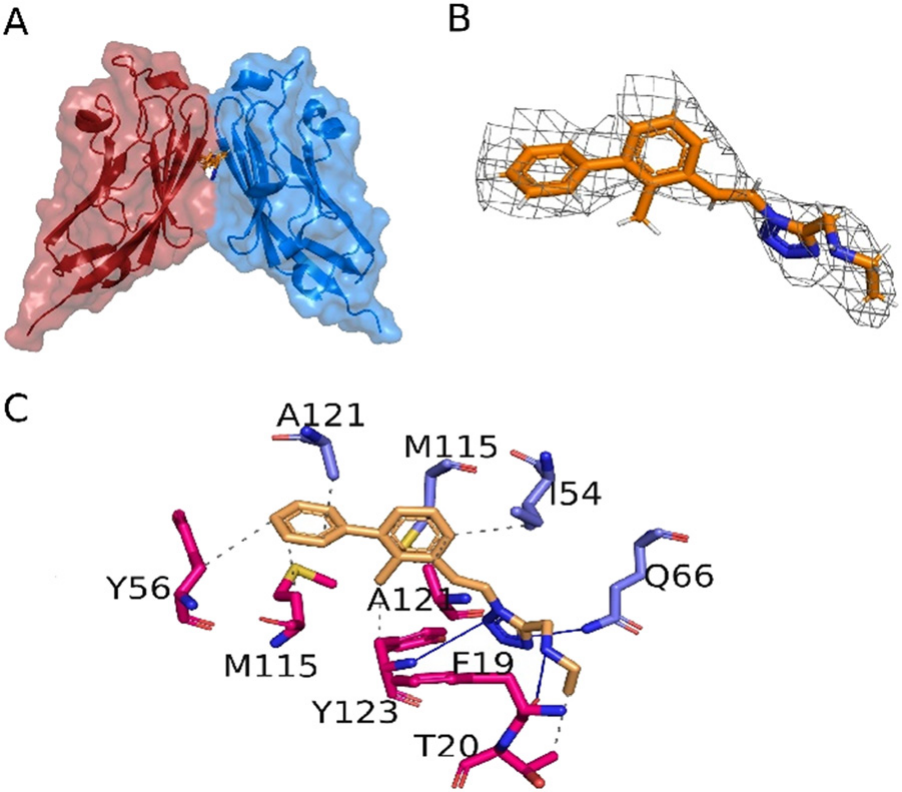
Binding of 10f to the PD-L1 dimer. A) Overlaid surface and cartoon representation of the PD-L1 dimer with subunit A in red and subunit B in blue. Compound 10f is located in the cleft at the interface (orange stick representation). B) The electron density around compound 10f. C) Interactions of PD-L1 with 10f; hydrophobic interactions are shown as gray dashed lines, whereas hydrogen bonds are depicted as blue lines. Deposited PDB ID: 8P64.

The interactions between 10f and the PD-L1 dimer were elucidated at the molecular level by X-ray structure analysis (resolution of 3.31 Å) of the cocrystal (Fig. 3A, Table S1†). The electron density well explains the inhibitor structure (Fig. 3B) and its interactions with the PD-L1 chains (Fig. 3C). Compound 10f interacts with PD-L1 through a series of hydrophobic interactions and hydrogen bonds in accordance with our modelling studies (Fig. 1A). The biphenyl moiety is stabilized between both PD-L1 subunits *via* hydrophobic interactions with _A_PD-L1 Tyr56, Met115 and Tyr123 as well as _B_PD-L1 Ala121, Met115 and Ile54, all well-known from previously reported similar co-structures.^29^ The tetrazole moiety likely not only increases the solubility of compound 10f, but also provides additional interaction *via* hydrogen bonds with the _A_Phe19 carbonyl backbone and _A_Tyr123 nitrogen as well as _B_Gln66 side chain amine further stabilizing the inhibitor and enhancing its affinity towards PD-L1. The compound with the highest affinity to PD-L1 is 10p and has a MW of 421 Dalton and a promising CNS penetration prediction based on the CNS-MPO score of 4.75, which is remarkably high.^30^ In view of the increasing interest to target glioblastoma with immune checkpoint inhibitors, 10p and similar compounds deserve future attention.^31^

## 3. Conclusions

In conclusion, based on our previously described pharmacophore model, we designed a novel scaffold, 1,5-disubstituted tetrazoles, as a PD-L1 dimerizer. The tetrazoles can be synthesized in a convenient fashion by using multicomponent reaction chemistry with high variations of amines and aldehydes. The overall good, predicted physicochemical properties make the tetrazole scaffold promising for further optimization.

## Conflicts of interest

The authors declare no conflicts of interest.

## Supplementary Material

MD-015-D3MD00746D-s001
