# Supplementary material for: 1,5-Disubstituted tetrazoles as PD-1/PD-L1 antagonists
Source: RSC Med Chem. 2024 Feb 21;15(4):1210–5. doi: 10.1039/d3md00746d (PMC11042242; doi:10.1039/d3md00746d)
Supplement: MD-015-D3MD00746D-s001 [file MD-015-D3MD00746D-s001.pdf]

Electronic Supplementary Material (ESI) for RSC Medicinal Chemistry.
This journal is © The Royal Society of Chemistry 2024

Supporting Information

1,5-disubstituted Tetrazoles as PD-1/PD-L1 Antagonist

Robin van der Straat[1], Rosalie Draijer[1], Ewa Surmiak[2], Roberto Butera[1], Lennart Land[1], Katarzyna Magiera-Mularz[2], Bogdan Musielak[2], Jacek Plewka[2], Tad A. Holak[2] and Alexander Dömling[†*3]

[1] Department of Pharmacy, Drug Design Group, University of Groningen, A. Deusinglaan 1, Groningen 9700AV, The Netherlands.

[2] Department of Organic Chemistry, Faculty of Chemistry, Jagiellonian University, 30-387 Kraków, Poland

[3] Department of Innovative Chemistry, Palacký University, CATRIN, Šlechtitelů 241/27, 779 00 Olomouc, Czech Republic

[†]corresponding author
[*]E-mail: alexander.domling@upol.cz

Table of Contents

General information 3
Experimental procedures and analytical data 4
NMR spectra 11
NMR binding assay 30
Protein expression and purification 30
PD-L1 cocrystalization 30
Crystal structure determination and refinement 31
Homogenous Time Resolved Fluorescence 32
References: 34

General information

Reagents were available from commercial suppliers (Sigma Aldrich, ABCR, Acros and AK Scientific) and used without any purification unless otherwise noted. Thin layer chromatography was performed on Fluka precoated silica gel plates (0.20 mm thick, particle size 25 μm). Flash chromatography was performed on a Teledyne ISCO Combiflash Rf, using RediSep Rf Normal-phase Silica Flash Columns (Silica Gel 60 Å, 230 – 400 mesh) and on a Reveleris® X2 Flash Chromatography, using Grace® Reveleris Silica flash cartridges (40 grams, 24 grams, 12 grams and 3 grams). All HTRF experiments were performed using a Cisbio Bioassays Human PD-1/PD-L1 biochemical binding assay. Nuclear magnetic resonance spectra were recorded on a Bruker Avance 500 spectrometer. Chemical shifts for ^{1}H NMR were reported in ppm relative to TMS (δ = 0.00 ppm) or the corresponding solvent peak ($CDCl_3$ δ = 7.26 ppm) and coupling constants were reported in Hertz (Hz). The following abbreviations were used for spin multiplicity: s = singlet, bs = broad singlet, d = doublet, dd = doublet of doublets, t = triplet, ddt = doublet of doublets of triplets, q = quartet, and m = multiplet. Chemical shifts for ^{13}C NMR were reported in ppm relative to the solvent peak (Chloroform-d δ = 77.2 ppm). High resolution mass spectra (HRMS) were recorded using an Orbitrap-Velos Proat a resolution of 60,000.

Experimental procedures and analytical data

<u>Procedure A</u>: General procedure for the Ugi tetrazole reaction

A mixture of aldehyde (1 eq.), amine (1 eq.) and scandium triflate (5 mol %) in DCM/MeOH (1:1, 1.0 M) was placed in a glass vial equipped with a magnetic stirring bar. After 15 minutes of stirring $TMSN_3$ (1 eq.) followed by isocyanide **9a-b** (1.5 eq.) was added to the mixture. This mixture was stirred for 24 hours at room temperature. Evaporation of the solvents was followed by purification by silica gel flash chromatography using PE-EA as eluent to obtain the corresponding products **10a-r**.

(E)-3-methyl-1-(1-(2-(2-methyl-[1,1'-biphenyl]-3-yl)vinyl)-1H-tetrazol-5-yl)-N-(2-morpholinoethyl)butan-1-amine (10a)

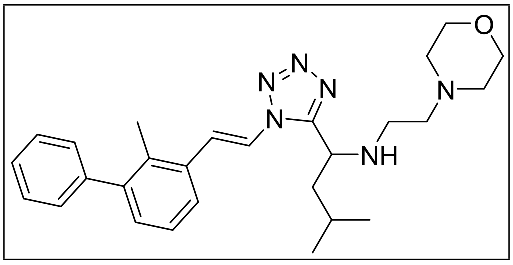

Synthesis according to procedure **A** afforded **10a** (134 mg, 0.29 mmol, 40 %) as a yellow oil; **^{1}H NMR** (500 MHz, $CDCl_3$) δ 7.93 (d, *J* = 14.2 Hz, 1H), 7.84 (d, *J* = 14.2 Hz, 1H), 7.49 (dd, *J* = 7.4, 1.7 Hz, 1H), 7.46 – 7.40 (m, 2H), 7.39 – 7.34 (m, 1H), 7.34 – 7.24 (m, 4H), 4.43 (t, *J* = 7.4 Hz, 1H), 3.72 – 3.62 (m, 4H), 2.66 – 2.58 (m, 1H), 2.55 – 2.34 (m, 7H), 2.32 (s, 3H), 2.06 (s, 1H), 1.85 – 1.77 (m, 1H), 1.77 – 1.69 (m, 1H), 1.67 – 1.56 (m, 1H), 0.99 (d, *J* = 6.5 Hz, 3H), 0.93 (d, *J* = 6.6 Hz, 3H). **^{13}C NMR** (126 MHz, $CDCl_3$) δ 155.4, 143.5, 141.7, 134.4, 133.7, 130.9, 129.3, 128.3, 127.2, 126.0, 125.3, 124.7, 120.6, 67.0, 57.8, 53.6, 52.6, 43.8, 43.5, 25.0, 22.8, 22.2, 17.4. HRMS (ESI) m/z calculated for $C_{27}H_{36}N_6O$ $[M+H]^+$: 461.3029, found $[M+H]^+$: 461.3012.

(E)-N-(2,3-dimethoxybenzyl)-2-methyl-1-(1-(2-(2-methyl-[1,1'-biphenyl]-3-yl)vinyl)-1H-tetrazol-5-yl)propan-1-amine (10b)

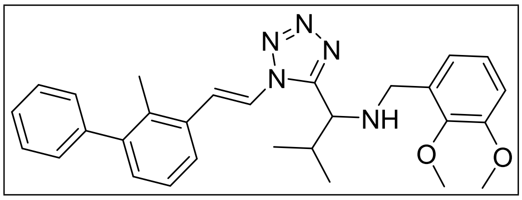

Synthesis according to procedure **A** afforded **10b** (130 mg, 0.26 mmol, 37 %) as a white solid. **mp:** 79 – 82 C; **^{1}H NMR** (500 MHz, $CDCl_3$) δ 7.85 (d, *J* = 14.2 Hz, 1H), 7.81 (d, *J* = 14.2 Hz, 1H), 7.48 – 7.40 (m, 3H), 7.38 – 7.34 (m, 1H), 7.32 – 7.27 (m, 2H), 7.27 – 7.23 (m, 2H), 6.99 (t, *J* = 7.9 Hz, 1H), 6.86 (dd, *J* = 8.2, 1.5 Hz, 1H), 6.77 (dd, *J* = 7.6, 1.5 Hz, 1H), 4.04 (d, *J* = 7.6 Hz, 1H), 3.85 (s, 3H), 3.80 (s, 3H), 3.69 (d, *J* = 13.0 Hz, 1H), 3.55 (d, *J* = 13.1 Hz, 1H), 2.30 (s, 3H), 2.19 (td, *J* = 9.7, 9.1, 5.6 Hz, 1H), 2.13 (dt, *J* = 13.8, 6.9 Hz, 1H), 1.03 (d, *J* = 6.7 Hz, 3H), 0.82 (d, *J* = 6.8 Hz, 3H). **^{13}C NMR** (126 MHz, $CDCl_3$) δ 154.7, 152.8, 147.5, 143.3, 141.8, 134.4, 133.8, 132.6, 130.7, 129.4, 128.3, 127.2, 126.0, 125.5, 124.4, 124.2, 122.1, 121.0, 112.0, 60.8, 59.65, 55.8, 47.3, 33.1, 19.5, 19.4, 17.4. HRMS (ESI) m/z calculated for $C_{29}H_{33}N_5O_2$ $[M+H]^+$: 484.2712, found $[M+H]^+$: 484.2700.

(E)-2-chloro-N-(furan-2-ylmethyl)-1-(1-(2-(2-methyl-[1,1'-biphenyl]-3-yl)vinyl)-1H-tetrazol-5-yl)ethan-1-amine (10c)

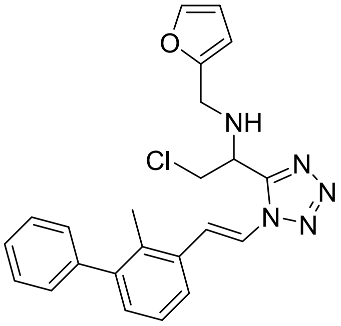

Synthesis according to procedure **A** afforded **10c** (88 mg, 0.21 mmol, 29 %) as a yellow/brown solid. **mp:** 71 – 73 C; **^{1}H NMR** (500 MHz, $CDCl_3$) δ 7.84 (d, *J* = 14.1 Hz, 1H), 7.75 (d, *J* = 14.1 Hz, 1H), 7.50 (dd, *J* = 7.2, 2.0 Hz, 1H), 7.46 – 7.41 (m, 2H), 7.40 – 7.35 (m, 1H), 7.33 – 7.26 (m, 5H), 6.28 (dd, *J* = 3.2, 1.9 Hz, 1H), 6.15 (d, *J* = 3.2 Hz, 1H), 4.54 (t, *J* = 6.5 Hz, 1H), 4.04 (dd, *J* = 11.3, 6.8 Hz, 1H), 3.96 (dd, *J* = 11.2, 6.2 Hz, 1H), 3.85 (d, *J* = 14.6 Hz, 1H), 3.74 (d, *J* = 14.6 Hz, 1H), 2.45 (s, 1H), 2.30 (s, 3H). **^{13}C NMR** (126 MHz, $CDCl_3$) δ 152.6, 151.7, 143.5, 142.7, 141.7, 134.5, 133.4, 131.1, 129.4, 128.3, 127.2, 126.1, 125.8, 125.5, 120.1, 110.6, 108.5, 53.8, 44.9, 43.3, 17.4. HRMS (ESI) m/z calculated for $C_{23}H_{22}ClN_5O$ $[M+H]^+$: 420.1591, found $[M+H]^+$: 420.1573.

(E)-1-cyclopropyl-1-(1-(2-(2-methyl-[1,1'-biphenyl]-3-yl)vinyl)-1H-tetrazol-5-yl)-N-(pyridin-4-ylmethyl)methanamine (10d)

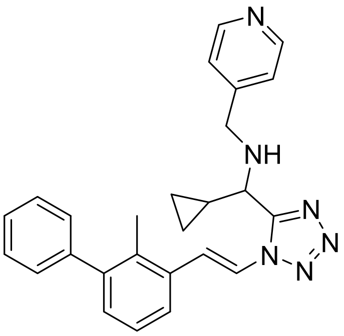

Synthesis according to procedure **A** afforded **10d** (148 mg, 0.35 mmol, 49 %) as an orange oil. **^{1}H NMR** (500 MHz, $CDCl_3$) δ 8.55 (dd, *J* = 5.9, 2.6 Hz, 2H), 7.90 (d, *J* = 14.2 Hz, 1H), 7.86 (d, *J* = 14.3 Hz, 1H), 7.47 – 7.42 (m, 3H), 7.40 – 7.35 (m, 1H), 7.33 – 7.27 (m, 4H), 7.22 (dd, *J* = 6.0, 2.7 Hz, 2H), 3.75 (d, *J* = 14.6 Hz, 1H), 3.71 (d, *J* = 14.4 Hz, 1H), 3.64 (d, *J* = 9.3 Hz, 1H), 2.30 (s, 3H), 1.39 – 1.32 (m, 1H), 0.94 – 0.85 (m, 1H), 0.80 – 0.72 (m, 1H), 0.63 – 0.51 (m, 1H), 0.47 – 0.34 (m, 2H). ^{13}C NMR (126 MHz, $CDCl_3$) δ 154.0, 149.6, 149.4, 148.4, 143.5, 141.6, 134.4, 133.4, 131.0, 129.3, 128.3, 127.2, 126.0, 125.2, 125.0, 123.1, 120.2, 58.7, 50.5, 17.4, 15.4, 5.4, 3.0. HRMS (ESI) m/z calculated for $C_{26}H_{26}N_6$ $[M+H]^+$: 423.2297, found $[M+H]^+$: 423.2280.

(E)-2,2-dimethyl-N-((1-(2-(2-methyl-[1,1'-biphenyl]-3-yl)vinyl)-1H-tetrazol-5-yl)methyl)propan-1-amine (10e)

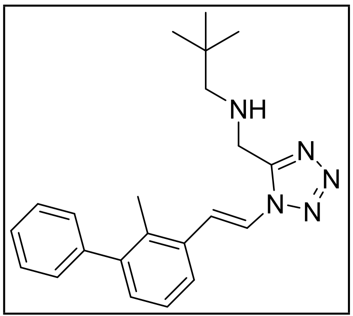

Synthesis according to procedure **A** afforded **10e** (58 mg, 0.16 mmol, 45 %) as a yellow solid. **mp:** 91 – 93 C; **^{1}H NMR** (500 MHz, $CDCl_3$) δ 7.87 (d, *J* = 14.2 Hz, 1H), 7.77 (d, *J* = 14.2 Hz, 1H), 7.49 (dt, *J* = 6.9, 1.4 Hz, 1H), 7.46 – 7.40 (m, 2H), 7.40 – 7.34 (m, 1H), 7.33 – 7.24 (m, 4H), 4.23 (s, 2H), 2.41 (s, 2H), 2.31 (s, 3H), 0.92 (s, 9H), 0.91 – 0.83 (m, 1H). **^{13}C NMR** (126 MHz, $CDCl_3$) δ 152.4, 143.5, 141.7, 134.5, 133.6, 131.0, 129.4, 128.3, 127.2, 126.1, 125.3, 125.0, 120.4, 62.1, 43.5, 31.7, 27.8, 17.5. HRMS (ESI) m/z calculated for $C_{22}H_{27}N_5$ $[M+H]^+$: 362.2344, found $[M+H]^+$: 362.2328.

(E)-N-((1-(2-(2-methyl-[1,1'-biphenyl]-3-yl)vinyl)-1H-tetrazol-5-yl)methyl)ethanamine (10f)

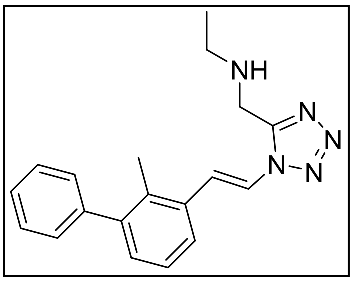

Synthesis according to procedure **A** afforded **10f** (35 mg, 0.11 mmol, 30 %) as an orange oil. **^{1}H NMR** (500 MHz, $CDCl_3$) δ 7.89 (d, *J* = 14.2 Hz, 1H), 7.67 (d, *J* = 14.2 Hz, 1H), 7.50 (d, *J* = 7.5 Hz, 1H), 7.46 – 7.40 (m, 2H), 7.40 – 7.34 (m, 1H), 7.34 – 7.27 (m, 4H), 4.22 (s, 2H), 2.74 (q, *J* = 7.1, 1.4 Hz, 2H), 2.31 (s, 3H), 1.30 – 1.24 (m, 1H), 1.15 (t, *J* = 7.1 Hz, 3H). **^{13}C NMR** (126 MHz, $CDCl_3$) δ 152.4, 143.5, 141.7, 134.5, 133.6, 131.0, 129.4, 128.3, 127.2, 126.1, 125.4, 125.4, 120.2, 44.1, 42.5, 17.4, 15.2. HRMS (ESI) m/z calculated for $C_{19}H_{21}N_5$ $[M+H]^+$: 320.1875, found $[M+H]^+$: 320.1863.

(E)-3-((1-(1-(2-(2-methyl-[1,1'-biphenyl]-3-yl)vinyl)-1H-tetrazol-5-yl)ethyl)amino)propan-1-ol (10g)

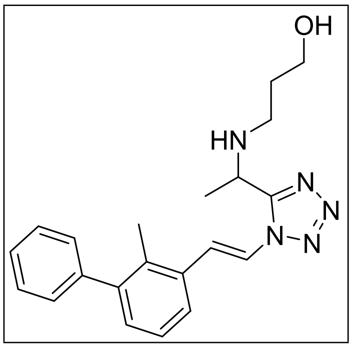

Synthesis according to procedure **A** afforded **10g** (29 mg, 0.08 mmol, 16 %) as a yellow oil. **^{1}H NMR** (500 MHz, $CDCl_3$) δ 7.86 (d, *J* = 14.1 Hz, 1H), 7.61 (dd, *J* = 14.1, 1.3 Hz, 1H), 7.51 (dd, *J* = 7.3, 1.8 Hz, 1H), 7.44 (t, *J* = 7.1, 1.0 Hz, 2H), 7.38 (t, 1H), 7.34 – 7.28 (m, 4H), 4.39 (q, 1H), 3.78 (t, *J* = 5.6 Hz, 2H), 2.75 (ddq, *J* = 17.8, 11.7, 6.2 Hz, 2H), 2.31 (s, 3H), 2.27 – 1.96 (m, 1H), 1.73 (p, *J* = 6.0 Hz, 2H), 1.59 (d, *J* = 6.8 Hz, 3H). **^{13}C NMR** (126 MHz, $CDCl_3$) δ 155.9, 143.5, 141.7, 134.5, 133.4, 131.1, 129.4, 128.3, 127.3, 126.2, 125.8, 125.4, 119.6, 62.7, 49.3, 46.5, 31.8, 20.8, 17.5. HRMS (ESI) m/z calculated for $C_{21}H_{25}N_5O$ $[M+H]^+$: 364.2137, found $[M+H]^+$: 364.2122.

(1r,3r)-3-(((1-((E)-2-(2-methyl-[1,1'-biphenyl]-3-yl)vinyl)-1H-tetrazol-5-yl)methyl)amino)adamantan-1-ol (10h)

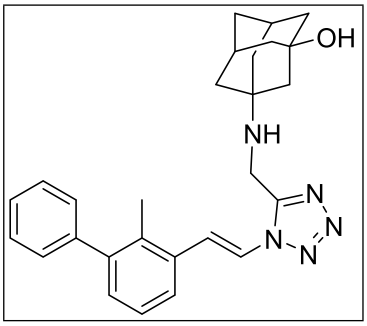

Synthesis according to procedure **A** afforded **10h** (40 mg, 0.09 mmol, 18 %) as a yellow oil. **^{1}H NMR** (500 MHz, $CDCl_3$) δ 7.87 (d, *J* = 14.2 Hz, 1H), 7.74 (dd, *J* = 14.2, 1.0 Hz, 1H), 7.48 (d, *J* = 7.5, 1.6 Hz, 1H), 7.44 (t, *J* = 7.4, 6.5, 1.1 Hz, 2H), 7.38 (t, 1H), 7.33 – 7.28 (m, 4H), 4.23 (s, 2H), 2.32 (m, 5H), 1.86 – 1.30 (m, 14H). **^{13}C NMR** (126 MHz, $CDCl_3$) δ 153.0, 143.5, 141.7, 134.5, 133.7, 131.0, 129.4, 128.4, 127.3, 126.2, 125.3, 125.0, 120.3, 69.7, 54.6, 50.2, 44.5, 41.3, 35.1, 35.1, 30.8, 17.5. HRMS (ESI) m/z calculated for $C_{27}H_{31}N_5O$ $[M+H]^+$: 442.2607, found $[M+H]^+$: 442.2592.

(E)-3-((1-(2-(2-methyl-[1,1'-biphenyl]-3-yl)vinyl)-1H-tetrazol-5-yl)methyl)-1,3-oxazepane (10i)

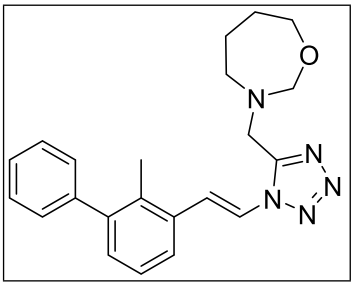

Synthesis according to procedure **A** afforded **10i** (28 mg, 0.07 mmol, 15 %) as a brown oil. **^{1}H NMR** (500 MHz, $CDCl_3$) δ 7.86 (d, *J* = 14.2 Hz, 1H), 7.56 (d, *J* = 14.2 Hz, 1H), 7.46 (dt, 1H), 7.43 (d, *J* = 7.6 Hz, 2H), 7.37 (t, 1H), 7.32 – 7.27 (m, 4H), 4.44 (s, 2H), 4.31 (s, 2H), 3.80 (t, *J* = 5.4 Hz, 2H), 2.86 (t, 2H), 2.30 (s, 3H), 1.92 – 1.81 (m, 4H). **^{13}C NMR** (126 MHz, $CDCl_3$) δ 151.5, 143.5,

141.7, 134.5, 133.6, 131.0, 129.4, 128.3, 127.2, 126.1, 125.4, 125.3, 112.0, 84.8, 69.8, 52.6, 44.7, 29.7, 24.7, 17.5. HRMS (ESI) m/z calculated for $C_{22}H_{25}N_5O$ $[M+H]^+$: 376.2137, found $[M+H]^+$: 376.2124.

(E)-5-(((1-(2-(2-methyl-[1,1'-biphenyl]-3-yl)vinyl)-1H-tetrazol-5-yl)methyl)amino)pentan-1-ol (10j)

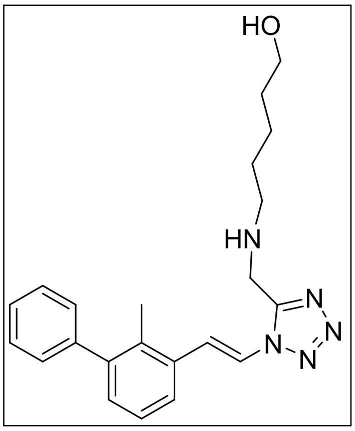

Synthesis according to procedure **A** afforded **10j** (18 mg, 0.05 mmol, 10 %) as a yellow oil. ^1H NMR (500 MHz, $CDCl_3$) δ 7.87 (d, *J* = 14.2 Hz, 1H), 7.66 (d, *J* = 14.3 Hz, 1H), 7.50 (dd, *J* = 7.3, 1.8 Hz, 1H), 7.46 – 7.40 (m, 2H), 7.40 – 7.35 (m, 1H), 7.33 – 7.28 (m, 4H), 4.22 (s, 2H), 3.63 (t, *J* = 6.5 Hz, 2H), 2.70 (t, *J* = 7.0 Hz, 2H), 2.31 (s, 3H), 2.17 (s, 1H), 1.63 – 1.50 (m, 4H), 1.48 – 1.38 (m, 2H), 1.27 – 1.24 (m, 1H). **^{13}C NMR** (126 MHz, $CDCl_3$) δ 152.3, 143.5, 141.7, 134.5, 133.5, 131.0, 129.4, 128.3, 127.2, 126.1, 125.4, 125.4, 120.1, 62.8, 49.6, 42.7, 32.5, 29.7, 23.5, 17.4. HRMS (ESI) m/z calculated for $C_{22}H_{27}N_5O$ $[M+H]^+$: 378.2294, found $[M+H]^+$: 378.2279.

(E)-3-((1-(2-(2-methyl-[1,1'-biphenyl]-3-yl)vinyl)-1H-tetrazol-5-yl)methyl)oxazolidine (10k)

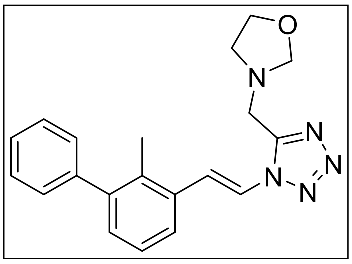

Synthesis according to procedure **A** afforded **10k** (28 mg, 0.08 mmol, 16%) as a brown oil. **^1H NMR** (500 MHz, $CDCl_3$) δ 7.93 (d, *J* = 14.2 Hz, 1H), 7.69 (d, *J* = 14.3 Hz, 1H), 7.51 (dd, *J* = 7.2, 2.0 Hz, 1H), 7.44 (t, *J* = 8.1, 6.6 Hz, 2H), 7.37 (t, *J* = 7.0, 6.5, 1.5 Hz, 1H), 7.33 – 7.27 (m, 4H), 4.38 (s, 2H), 4.14 (s, 2H), 3.88 (t, *J* = 6.9 Hz, 2H), 3.02 (t, *J* = 7.0 Hz, 2H), 2.31 (s, 3H). **^{13}C NMR** (126 MHz, $CDCl_3$) δ 151.1, 143.5, 141.7, 134.6, 133.5, 131.1, 129.4, 128.4, 127.3, 126.2, 125.8, 125.5, 112.1, 86.7, 63.3, 51.6, 47.0, 17.5. HRMS (ESI) m/z calculated for $C_{20}H_{21}N_5O$ $[M+H]^+$: 348.1824, found $[M+H]^+$: 348.1809.

(E)-3-((1-(2-(2-methyl-[1,1'-biphenyl]-3-yl)vinyl)-1H-tetrazol-5-yl)methyl)-1,3-oxazinane (10l)

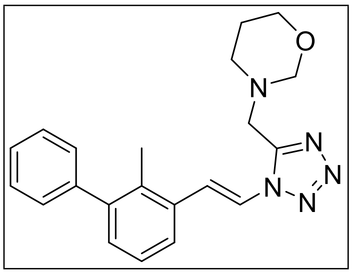

Synthesis according to procedure **A** afforded **10l** (20 mg, 0.06 mmol, 11%) as a brown oil. **^{1}H NMR** (500 MHz, $CDCl_3$) δ 7.88 (d, *J* = 14.2 Hz, 1H), 7.58 (dd, *J* = 14.2, 0.9 Hz, 1H), 7.48 (d, 1H), 7.43 (t, *J* = 7.4, 1.1 Hz, 2H), 7.37 (t, 1H), 7.33 – 7.28 (m, 4H), 4.40 (s, 2H), 4.30 (s, 2H), 3.90 (t, *J* = 5.4 Hz, 2H), 2.91 (t, *J* = 5.6 Hz, 2H), 2.31 (s, 3H), 1.40 – 1.28 (m, 2H). **^{13}C NMR** (126 MHz, $CDCl_3$) δ 151.0, 143.5, 141.7, 134.5, 133.5, 131.1, 129.4, 128.3, 127.2, 126.2, 125.6, 125.4, 120.1, 84.8, 68.1, 49.2, 44.6, 25.0, 21.7, 17.5. HRMS (ESI) m/z calculated for $C_{21}H_{23}N_5O$ $[M+H]^+$: 362.1981, found $[M+H]^+$: 362.1959.

(1r,3r)-3-(((1-((E)-3-(2,3-dihydrobenzo[b][1,4]dioxin-6-yl)-2-methylstyryl)-1H-tetrazol-5-yl)methyl)amino)adamantan-1-ol (10m)

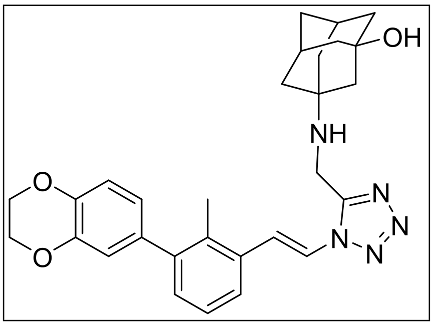

Synthesis according to procedure **A** afforded **10m** (183 mg, 0.37 mmol, 73%) as an off-white solid. **mp:** 171 – 173 C; **^{1}H NMR** (500 MHz, $CDCl_3$) δ 7.84 (d, *J* = 14.2 Hz, 1H), 7.71 (d, *J* = 14.3 Hz, 1H), 7.48 – 7.42 (m, 1H), 7.31 – 7.20 (m, 2H), 6.91 (d, *J* = 8.1 Hz, 1H), 6.83 – 6.80 (m, 1H), 6.76 (dt, *J* = 7.8, 2.2 Hz, 1H), 4.31 (s, 4H), 4.21 (s, 2H), 2.36 – 2.24 (m, 5H), 2.08 – 2.01 (m, 1H), 1.74 – 1.62 (m, 6H), 1.63 – 1.57 (m, 4H), 1.56 – 1.47 (m, 2H), 1.26 (t, *J* = 7.3 Hz, 1H). **^{13}C NMR** (126 MHz, $CDCl_3$) δ 153.0, 143.2, 142.9, 142.9, 135.1, 134.6, 133.6, 131.0, 126.1, 125.1, 125.1, 122.6, 120.2, 118.3, 117.1, 69.7, 64.6, 64.5, 54.6, 50.2, 44.4, 41.3, 35.1, 30.8, 17.5. HRMS (ESI) m/z calculated for $C_{29}H_{33}N_5O_3$ $[M+H]^+$: 500.2661, found $[M+H]^+$: 500.2643.

(E)-5-(((1-(3-(2,3-dihydrobenzo[b][1,4]dioxin-6-yl)-2-methylstyryl)-1H-tetrazol-5-yl)methyl)amino)pentan-1-ol (10n)

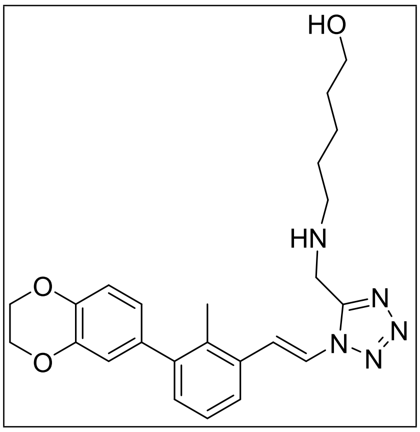

Synthesis according to procedure **A** afforded **10n** (32 mg, 0.07 mmol, 14%) as a yellow solid. **mp:** 125 – 127 C; **^{1}H NMR** (500 MHz, $CDCl_3$) δ 7.86 (d, *J* = 14.2 Hz, 1H), 7.65 (d, *J* = 14.2 Hz, 1H), 7.47 (dd, *J* = 6.8, 2.3 Hz, 1H), 7.30 – 7.22 (m, 2H), 6.92 (d, *J* = 8.2 Hz, 1H), 6.81 (d, 1H), 6.76 (dd, *J* = 8.2, 1.4 Hz, 1H), 4.31 (s, 4H), 4.21 (s, 2H), 3.62 (t, *J* = 6.5 Hz, 2H), 2.69 (t, *J* = 7.0 Hz, 2H), 2.32 (s, 3H), 1.61 – 1.50 (m, 4H), 1.47 – 1.38 (m, 2H), 1.32 – 1.20 (m, 1H), 0.94 – 0.84 (m, 1H). **^{13}C NMR** (126 MHz, $CDCl_3$) δ 152.3, 143.3, 142.9, 135.1, 134.7, 133.5, 131.1, 126.1, 125.5, 125.2, 122.6, 120.1, 118.3, 117.1, 64.6, 64.6, 62.8, 49.6, 42.7, 32.6, 29.7, 23.5, 17.5. HRMS (ESI) m/z calculated for $C_{24}H_{29}N_5O_3$ $[M+H]^+$: 436.2348, found $[M+H]^+$: 436.2336.

(E)-4-(((1-(3-(2,3-dihydrobenzo[b][1,4]dioxin-6-yl)-2-methylstyryl)-1H-tetrazol-5-yl)methyl)amino)butan-1-ol (10o)

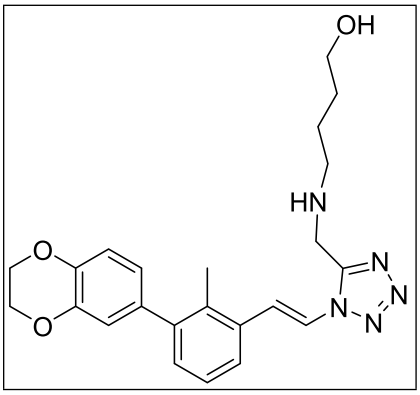

Synthesis according to procedure **A** afforded **10o** (24 mg, 0.06 mmol, 11%) as a brown oil. ^{1}H NMR (500 MHz, $CDCl_3$) δ 7.85 (d, *J* = 14.2 Hz, 1H), 7.56 (d, *J* = 14.1 Hz, 1H), 7.48 (dd, *J* = 6.8, 2.4 Hz, 1H), 7.30 – 7.22 (m, 2H), 6.92 (d, *J* = 8.2 Hz, 1H), 6.81 (d, *J* = 2.1 Hz, 1H), 6.76 (dd, *J* = 8.2, 2.1 Hz, 1H), 4.31 (s, 4H), 4.21 (s, 2H), 3.64 (t, *J* = 5.8 Hz, 2H), 2.74 (t, 2H), 2.32 (s, 3H), 1.69 – 1.60 (m, 4H), 1.28 – 1.12 (m, 1H), 0.96 – 0.82 (m, 1H). **^{13}C NMR** (126 MHz, $CDCl_3$) 152.1, 143.3, 142.9, 135.1, 134.7, 133.4, 131.2, 126.1, 126.0, 125.3, 122.6, 119.7, 118.3, 117.1, 64.6, 64.6, 62.7, 49.5, 42.4, 30.9, 26.9, 17.5. HRMS (ESI) m/z calculated for $C_{23}H_{27}N_5O_3$ $[M+H]^+$: 422.2192, found $[M+H]^+$: 422.2173.

(E)-3-((1-(1-(3-(2,3-dihydrobenzo[b][1,4]dioxin-6-yl)-2-methylstyryl)-1H-tetrazol-5-yl)ethyl)amino)propan-1-ol (10p)

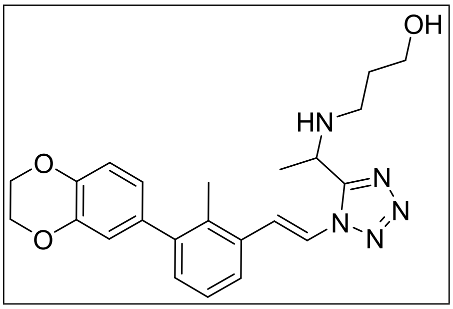

Synthesis according to procedure **A** afforded **10p** (26 mg, 0.06 mmol, 12%) as a yellow oil. **^{1}H NMR** (500 MHz, $CDCl_3$) δ 7.84 (d, *J* = 14.1 Hz, 1H), 7.60 (d, *J* = 14.1 Hz, 1H), 7.48 (dd, *J* = 6.9, 2.2 Hz, 1H), 7.33 – 7.22 (m, 2H), 6.92 (d, *J* = 8.2 Hz, 1H), 6.81 (d, *J* = 2.1 Hz, 1H), 6.76 (dd, *J* = 8.2, 2.0 Hz, 1H), 4.39 (q, *J* = 6.9 Hz, 1H), 4.31 (s, 4H), 4.31 – 4.28 (m, 1H), 3.77 (t, *J* = 5.6 Hz, 2H), 2.82 – 2.66 (m, 2H), 2.33 (s, 3H), 1.73 (p, *J* = 6.0 Hz, 2H), 1.58 (d, *J* = 6.9 Hz, 3H). **^{13}C NMR** (126 MHz, $CDCl_3$) δ 155.9, 143.2, 142.9, 135.0, 134.6, 133.3, 131.1, 126.1, 125.8, 125.2, 122.5, 119.5, 118.2, 117.1, 64.6, 64.5, 62.5, 49.2, 46.4, 31.8, 20.7, 17.5. HRMS (ESI) m/z calculated for $C_{23}H_{27}N_5O_3$ $[M+H]^+$: 422.2192, found $[M+H]^+$: 422.2174.

(E)-2-(((1-(3-(2,3-dihydrobenzo[b][1,4]dioxin-6-yl)-2-methylstyryl)-1H-tetrazol-5-yl)methyl)amino)ethan-1-ol (10q)

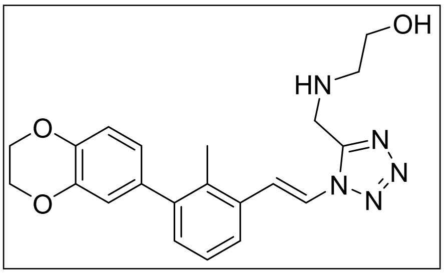

Synthesis according to procedure **A** afforded **10q** (20 mg, 0.05 mmol, 10%) as a brown oil. **^{1}H NMR** (500 MHz, $CDCl_3$) δ 7.85 (d, *J* = 14.2 Hz, 1H), 7.55 (d, *J* = 14.1 Hz, 1H), 7.47 (dd, *J* = 6.5, 2.6 Hz, 1H), 7.30 – 7.22 (m, 2H), 6.92 (d, *J* = 8.2 Hz, 1H), 6.81 (d, *J* = 2.0 Hz, 1H), 6.76 (dd, *J* = 8.2, 2.1 Hz, 1H), 4.32 – 4.29 (m, 6H), 4.26 (s, 2H), 3.73 (t, 2H), 2.87 (t, 2H), 2.32 (s, 3H). **^{13}C NMR** (126 MHz, $CDCl_3$) δ 152.3, 143.3, 142.9, 135.0, 134.7, 133.3, 131.2, 126.1, 125.9, 125.2, 122.6, 119.6, 118.3, 117.1, 64.6, 64.6, 61.4, 51.0, 42.3, 17.5. HRMS (ESI) m/z calculated for $C_{21}H_{23}N_5O_3$ $[M+H]^+$: 394.1879, found $[M+H]^+$: 394.1860.

(E)-3-((1-(3-(2,3-dihydrobenzo[b][1,4]dioxin-6-yl)-2-methylstyryl)-1H-tetrazol-5-yl)methyl)-1,3-oxazinane (10r)

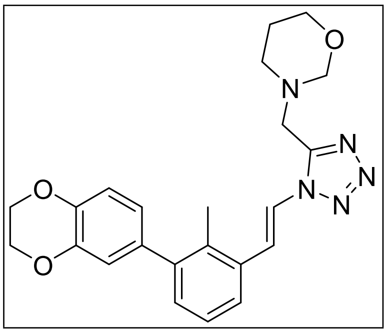

Synthesis according to procedure **A** afforded **10r** (63 mg, 0.15 mmol, 30%) as an off-white powder. **mp:** 83 – 85 C; **^{1}H NMR** (500 MHz, $CDCl_3$) δ 7.86 (d, J = 14.3 Hz, 1H), 7.56 (d, J = 14.2 Hz, 1H), 7.45 (dd, J = 6.8, 2.3 Hz, 1H), 7.31 – 7.22 (m, 2H), 6.92 (d, J = 8.3 Hz, 1H), 6.81 (d, J = 2.1 Hz, 1H), 6.79 – 6.74 (m, 1H), 4.39 (s, 2H), 4.31 (s, 4H), 4.30 (s, 2H), 3.90 (t, J = 5.4 Hz, 2H), 2.91 (t, J = 5.6 Hz, 2H), 2.32 (s, 3H), 1.77 (p, J = 5.4 Hz, 2H). **^{13}C NMR** (126 MHz, $CDCl_3$) δ 151.0, 143.2, 142.9, 142.8, 135.0, 134.6, 133.4, 131.0, 126.0, 125.5, 125.2, 122.5, 120.0, 118.2, 117.0, 84.6, 68.0, 64.5, 64.5, 49.2, 44.5, 21.6, 17.4. HRMS (ESI) m/z calculated for $C_{23}H_{25}N_5O_3$ $[M+H]^+$: 420.2035, found $[M+H]^+$: 420.2024.

NMR spectra

(E)-3-(2-isocyanovinyl)-2-methyl-1,1'-biphenyl (9a)

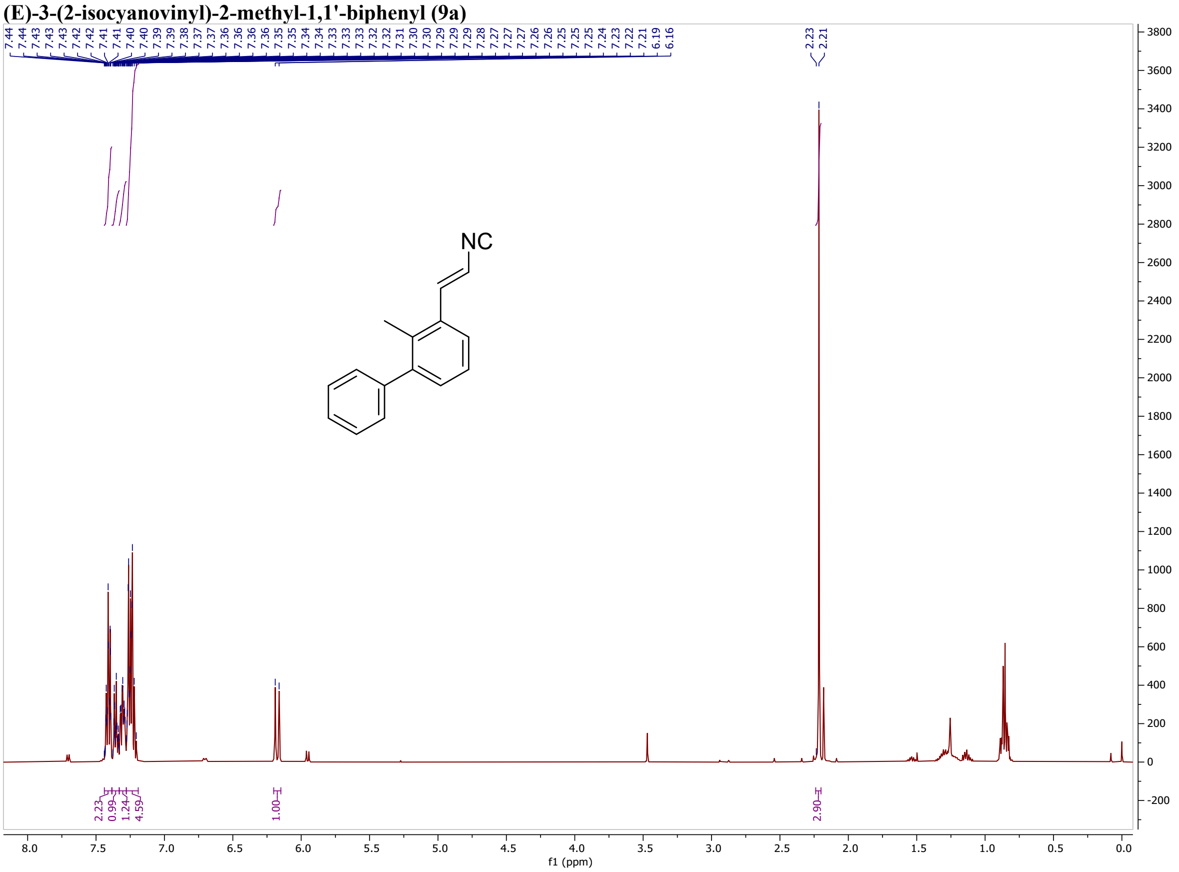

(E)-6-(3-(2-isocyanovinyl)-2-methylphenyl)-2,3-dihydrobenzo[b][1,4]dioxine (9b)

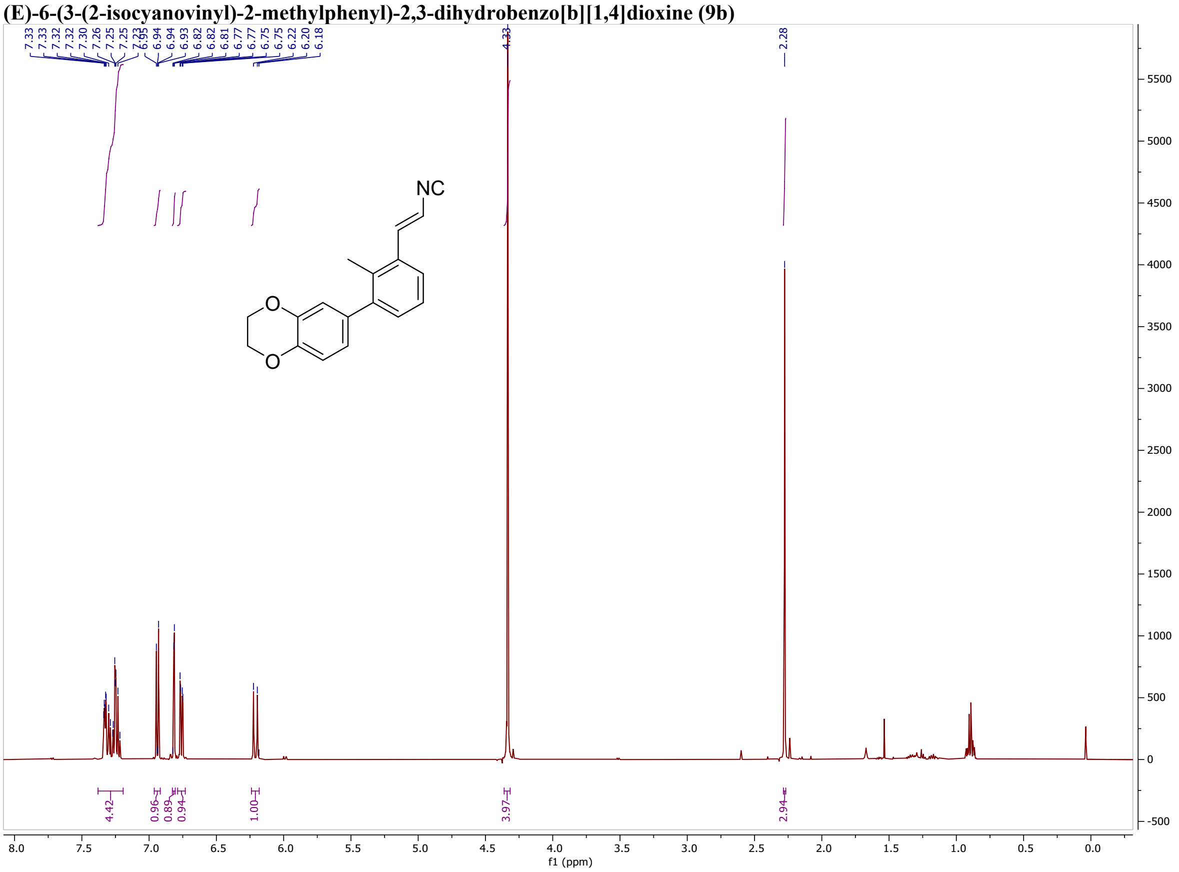

(E)-3-methyl-1-(1-(2-(2-methyl-[1,1'-biphenyl]-3-yl)vinyl)-1H-tetrazol-5-yl)-N-(2-morpholinoethyl)butan-1-amine (10a)

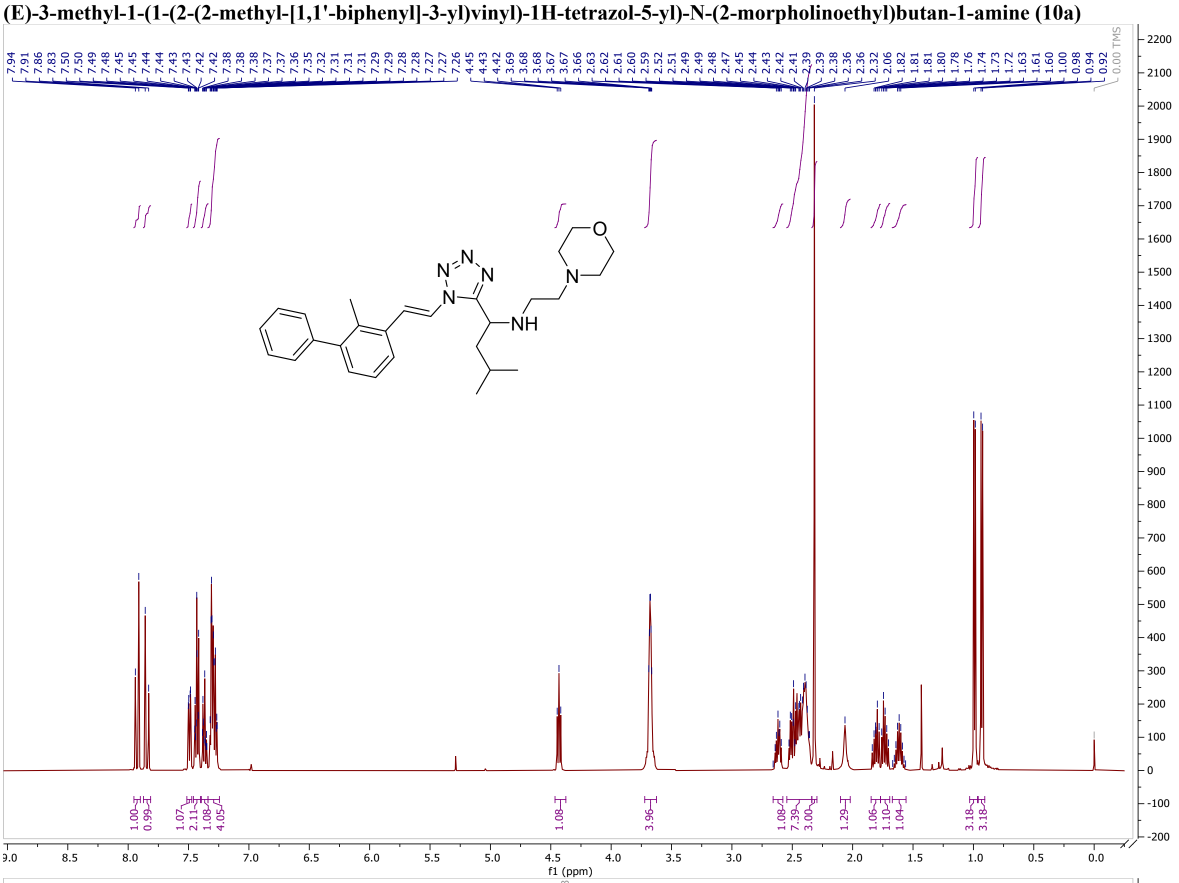

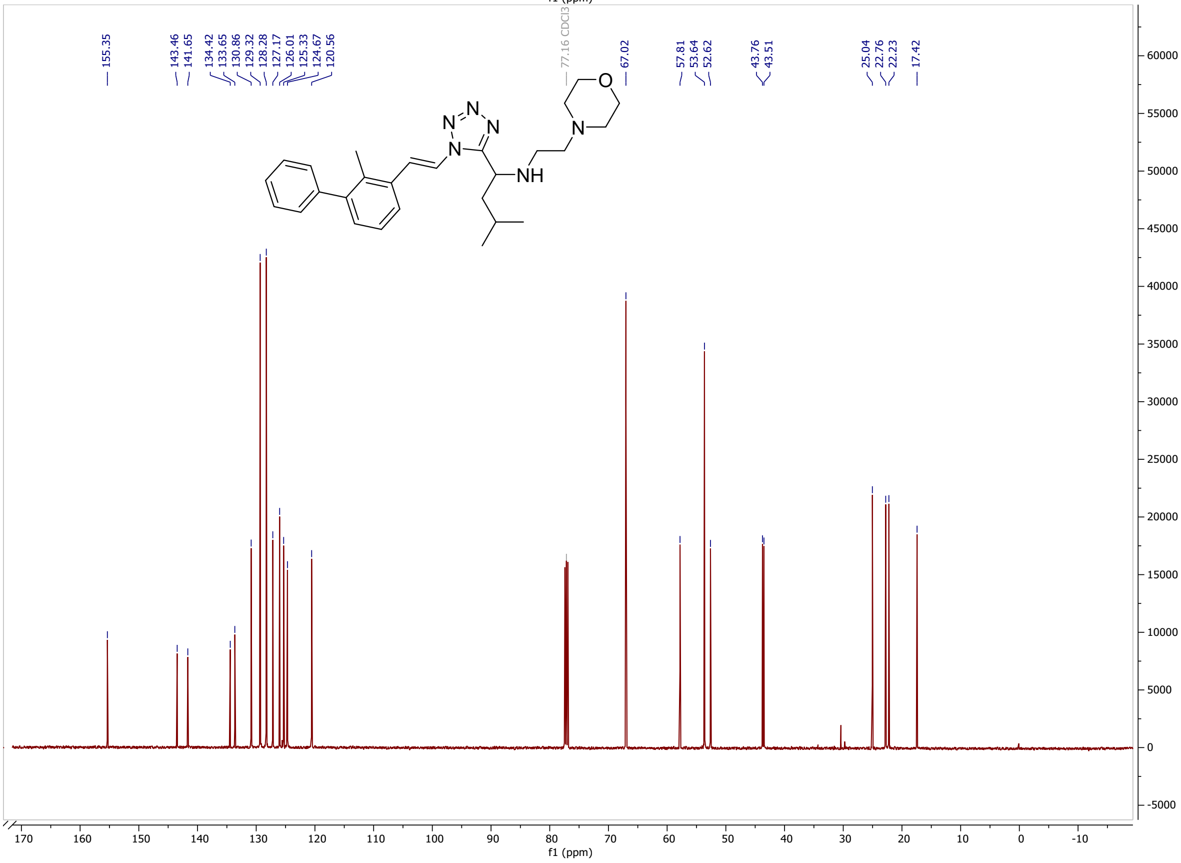

(E)-N-(2,3-dimethoxybenzyl)-2-methyl-1-(1-(2-(2-methyl-[1,1'-biphenyl]-3-yl)vinyl)-1H-tetrazol-5-yl)propan-1-amine (10b)

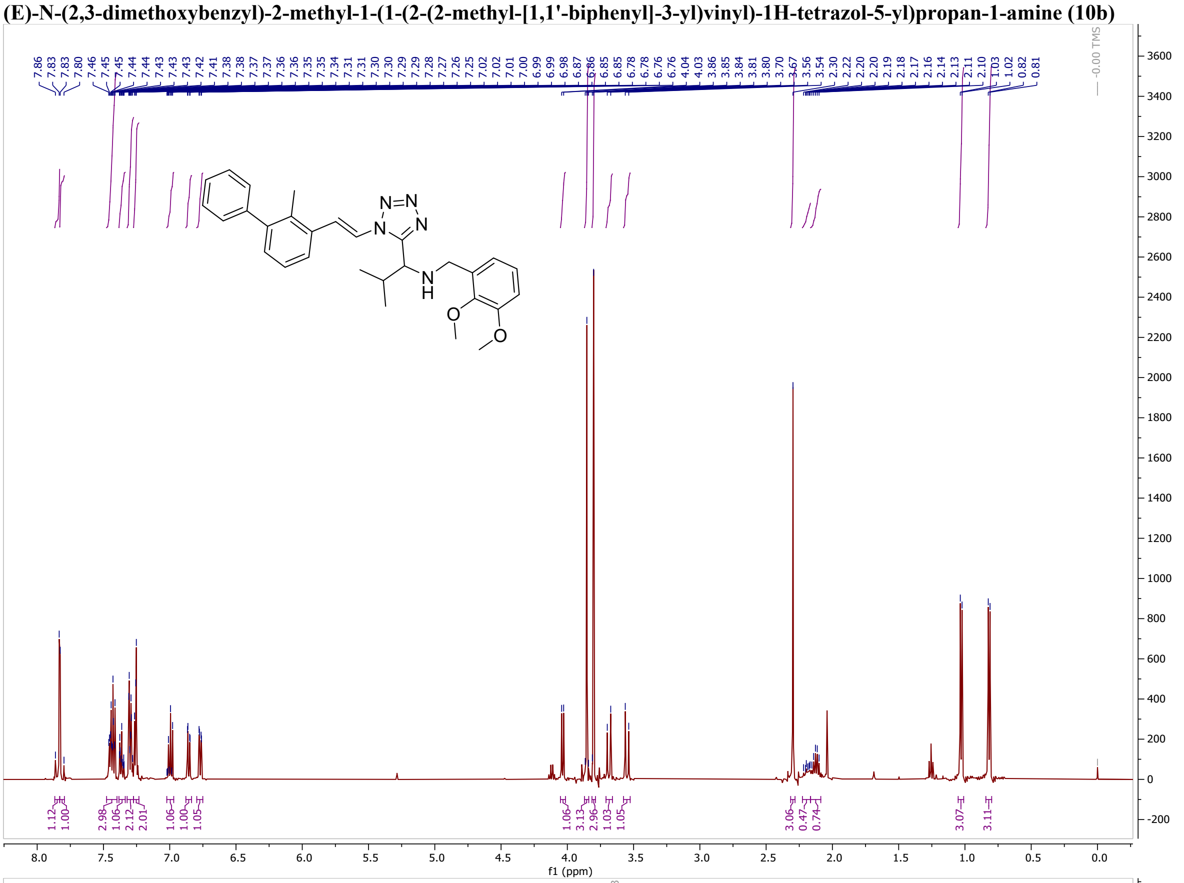

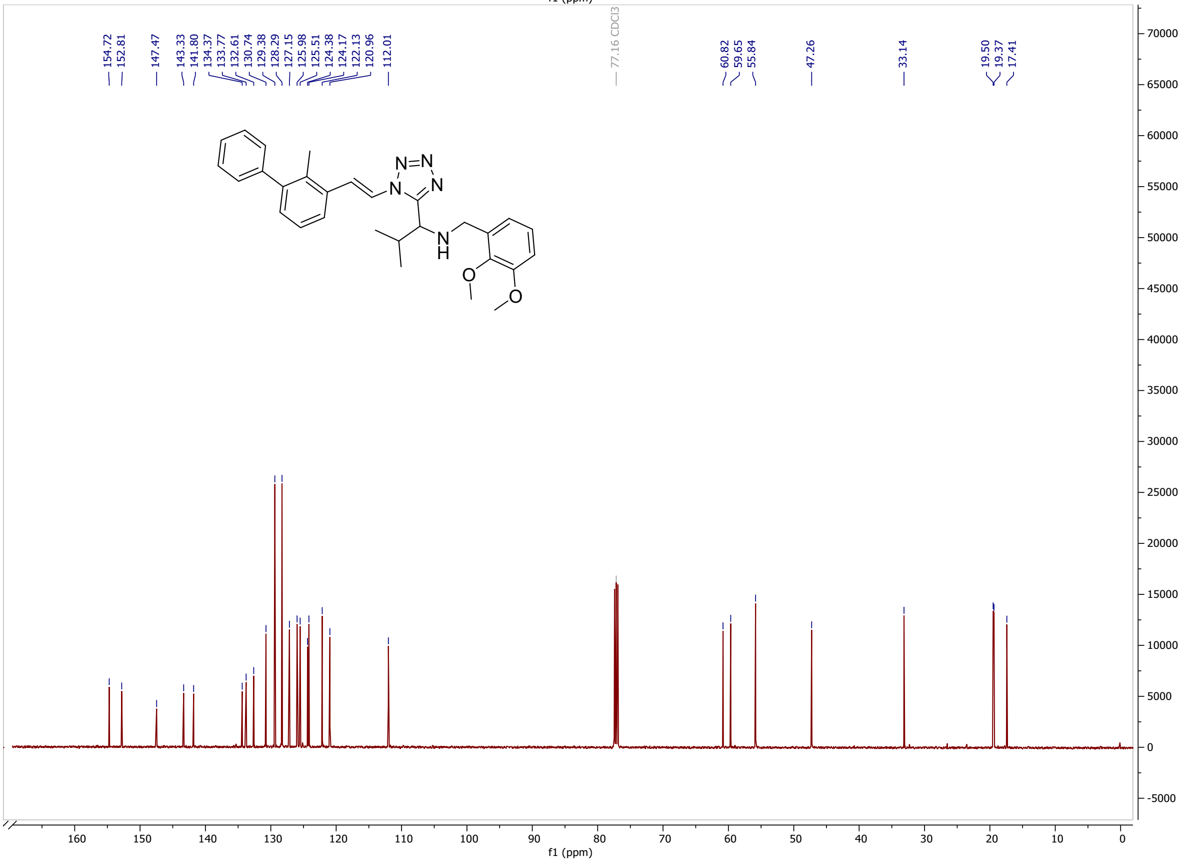

(E)-2-chloro-N-(furan-2-ylmethyl)-1-(1-(2-(2-methyl-[1,1'-biphenyl]-3-yl)vinyl)-1H-tetrazol-5-yl)ethan-1-amine (10c)

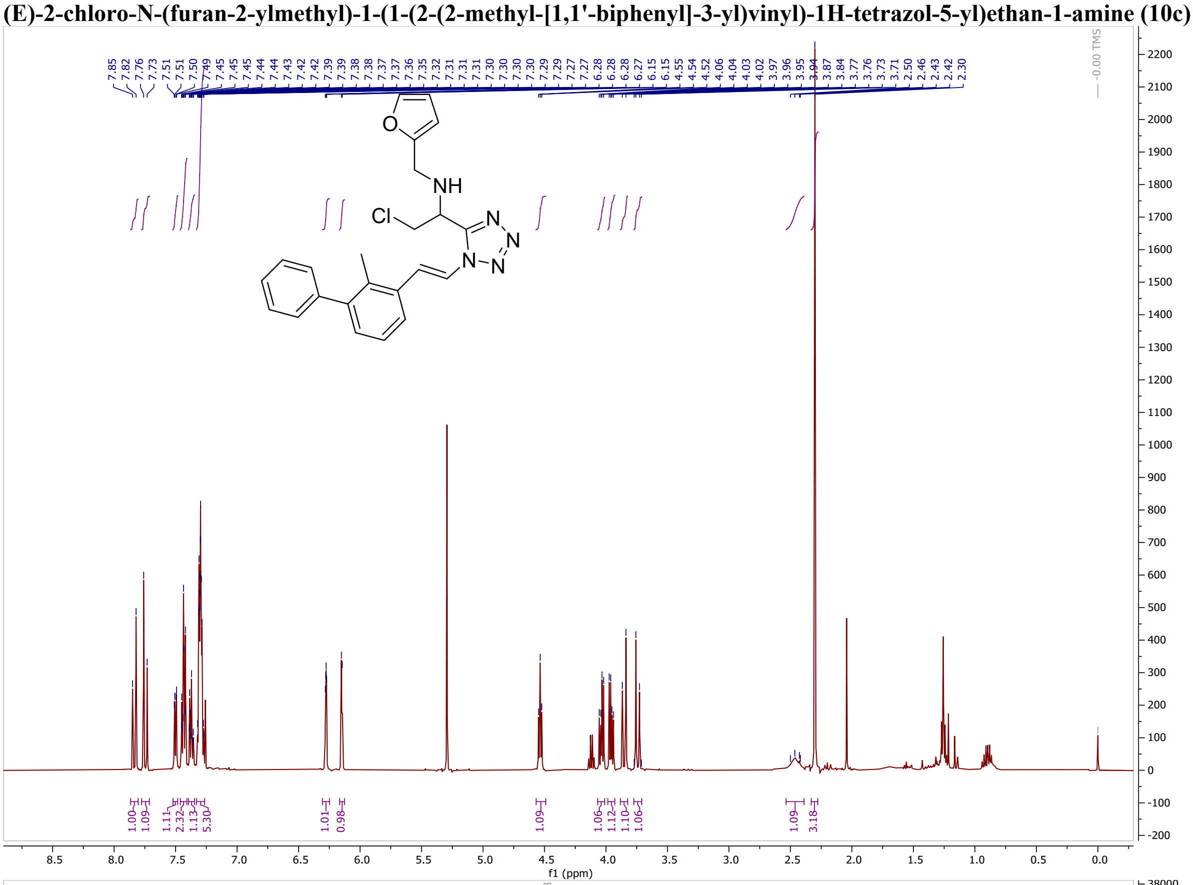

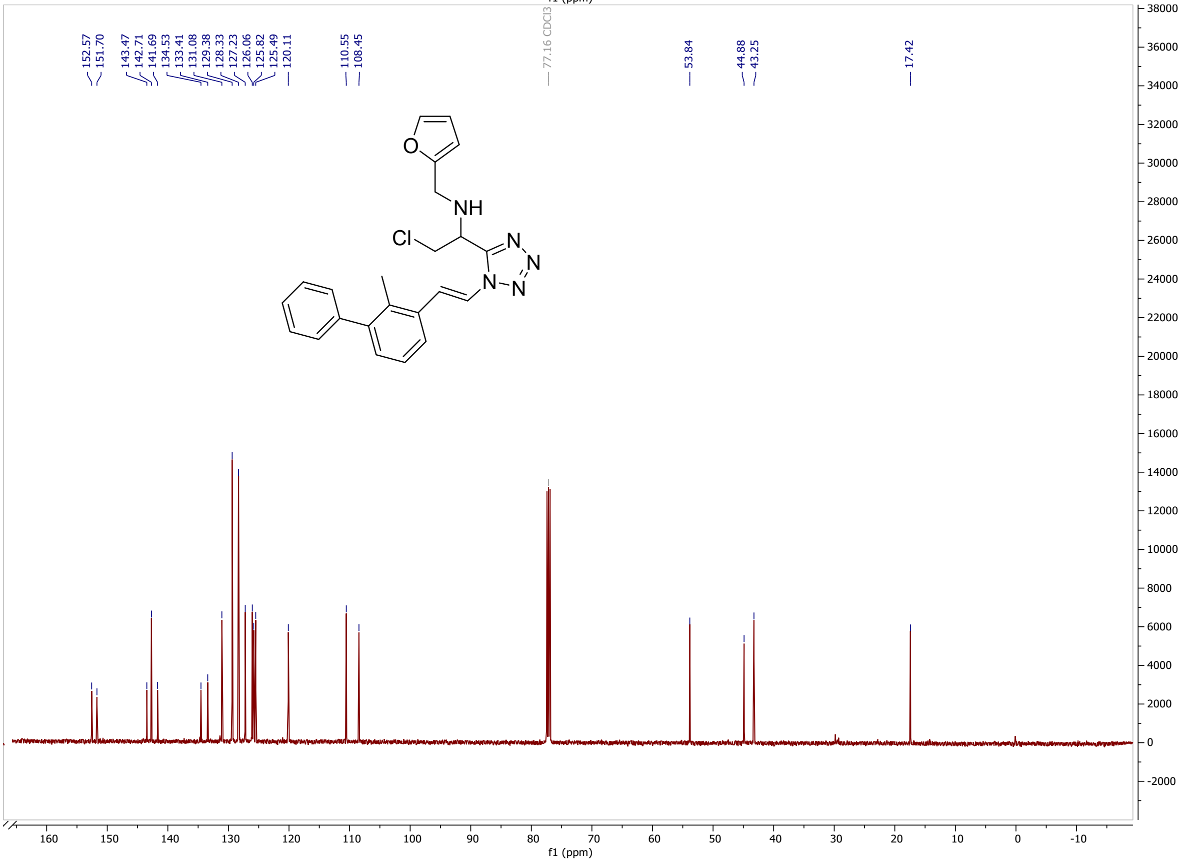

(E)-1-cyclopropyl-1-(1-(2-(2-methyl-[1,1'-biphenyl]-3-yl)vinyl)-1H-tetrazol-5-yl)-N-(pyridin-4-ylmethyl)methanamine (10d)

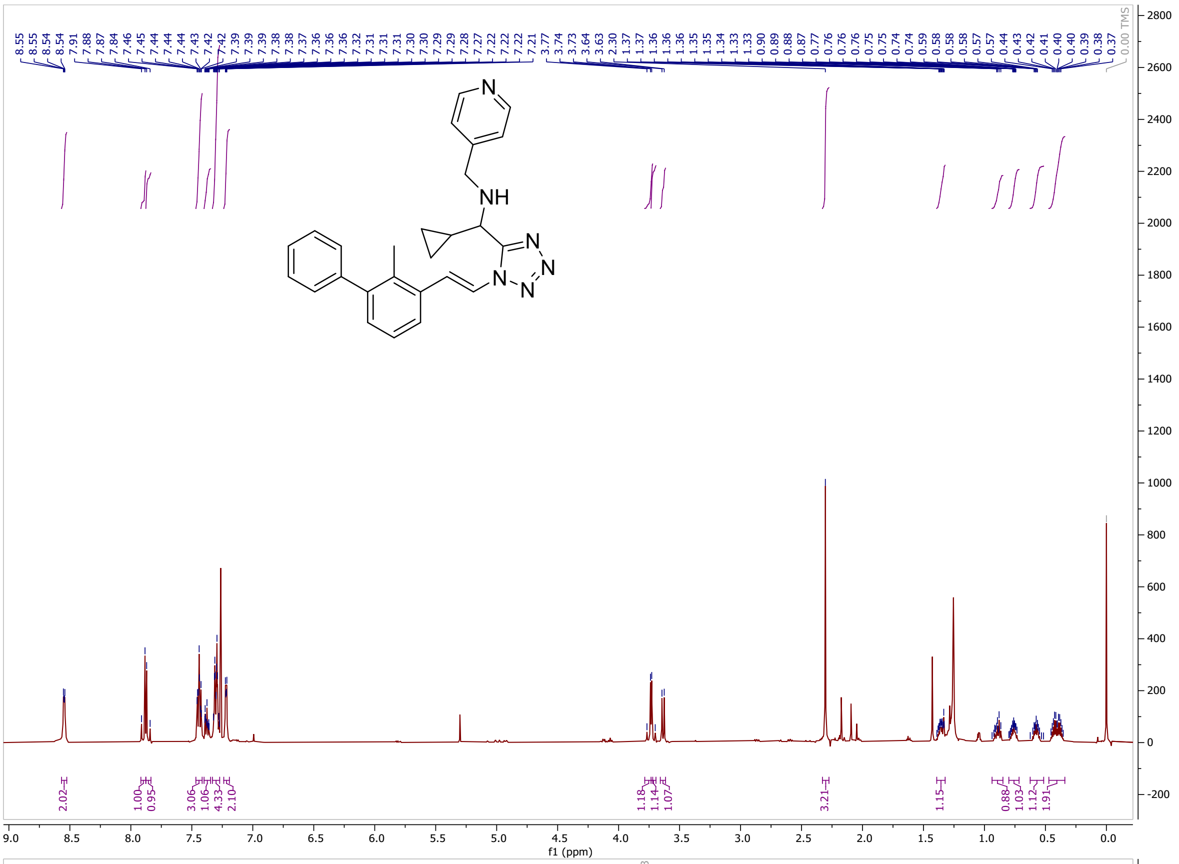

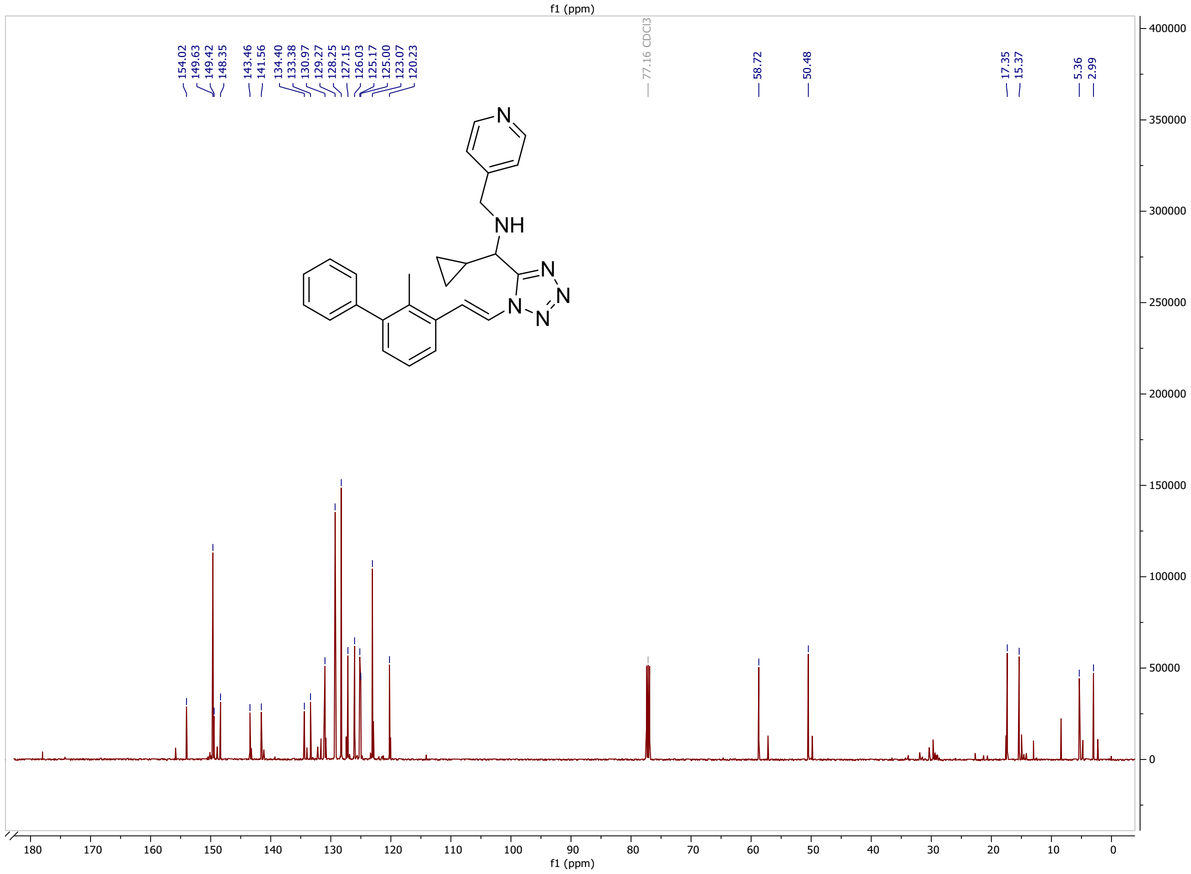

(E)-2,2-dimethyl-N-((1-(2-(2-methyl-[1,1'-biphenyl]-3-yl)vinyl)-1H-tetrazol-5-yl)methyl)propan-1-amine (10e)

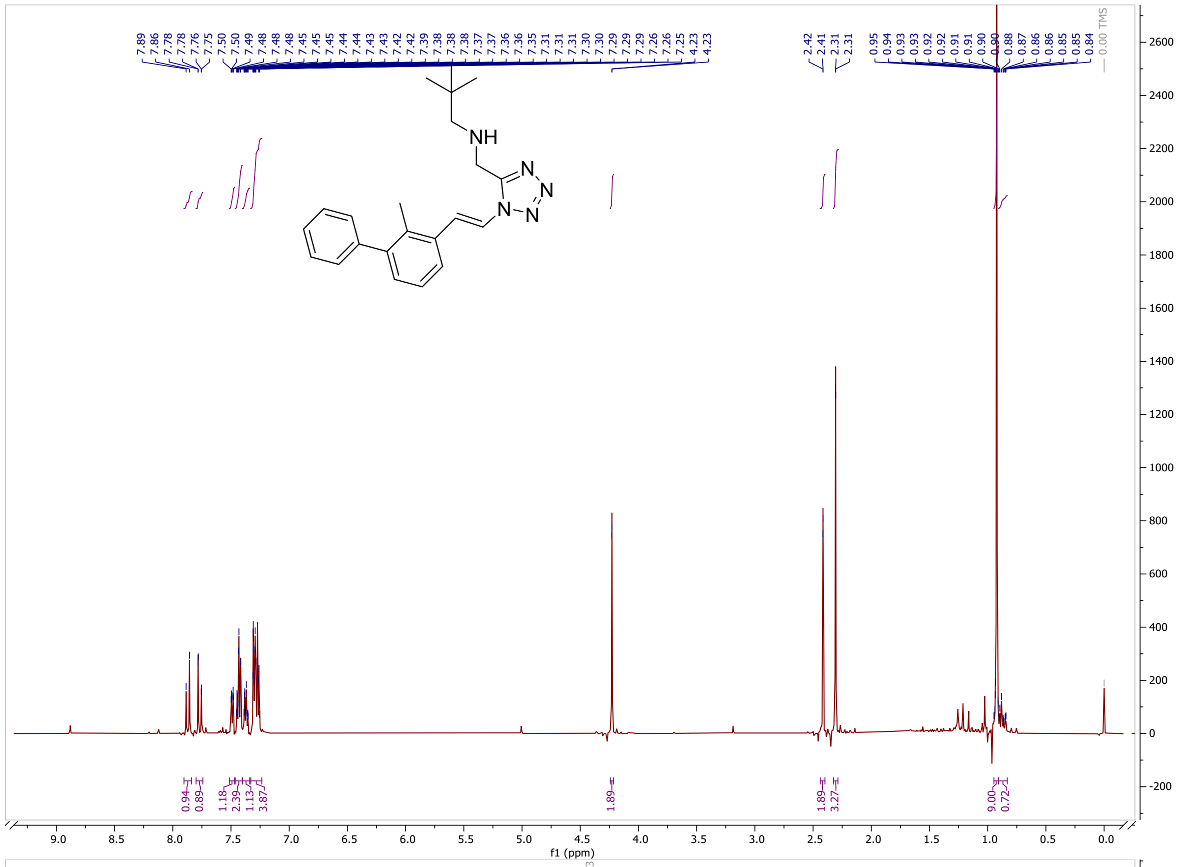

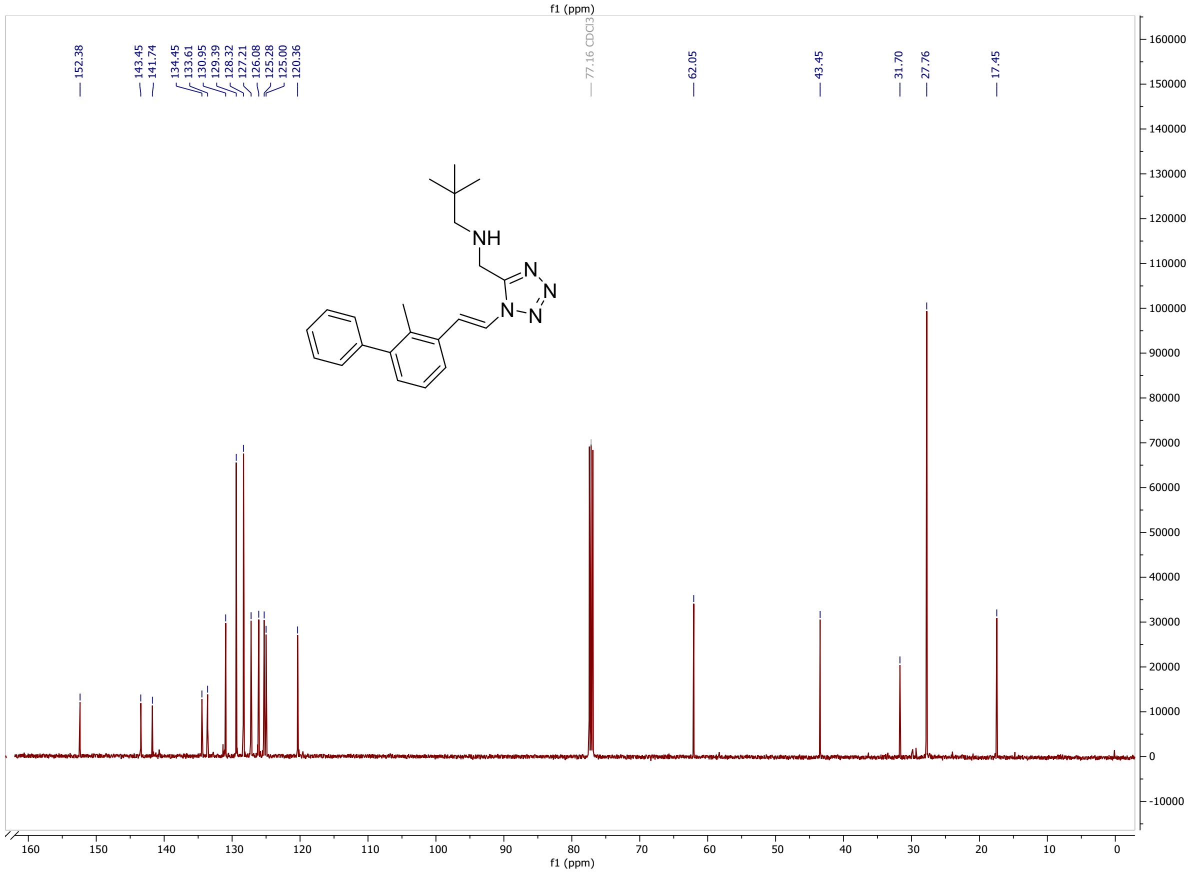

(E)-N-((1-(2-(2-methyl-[1,1'-biphenyl]-3-yl)vinyl)-1H-tetrazol-5-yl)methyl)ethanamine (10f)

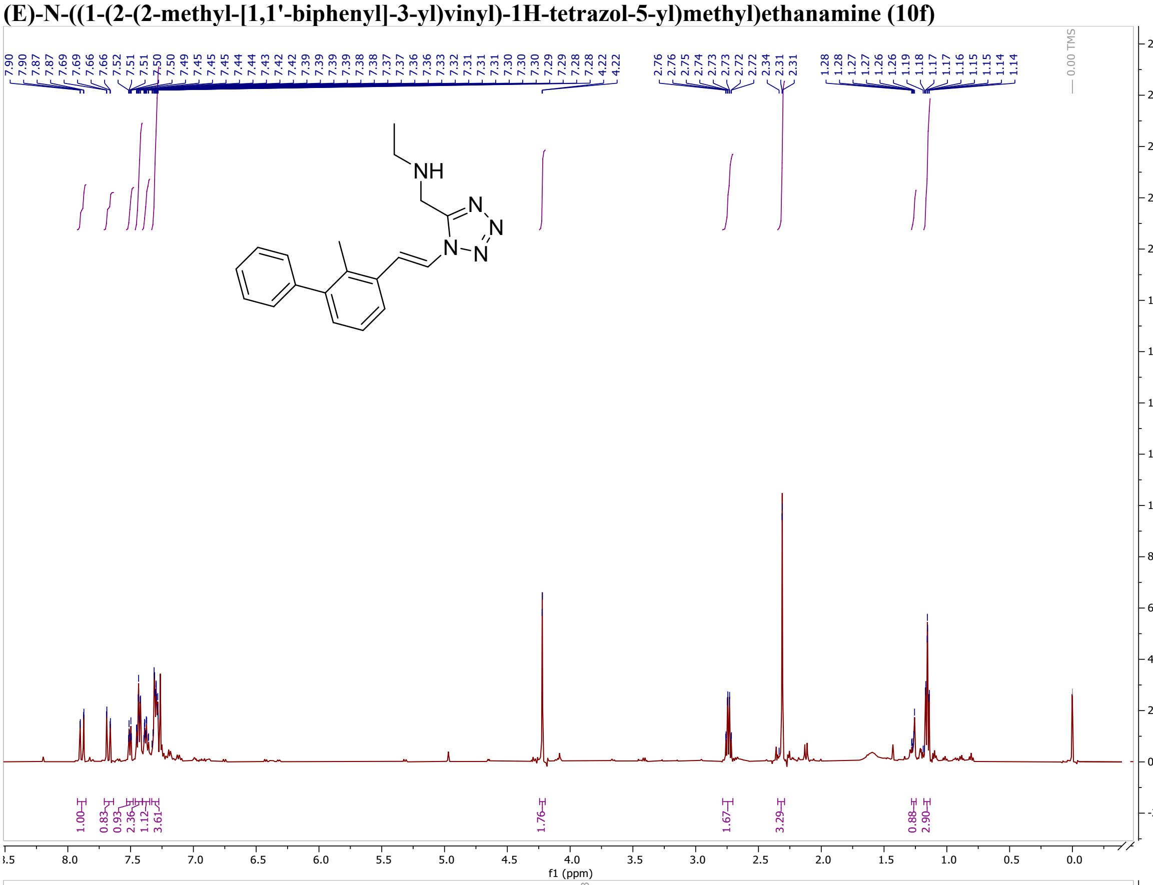

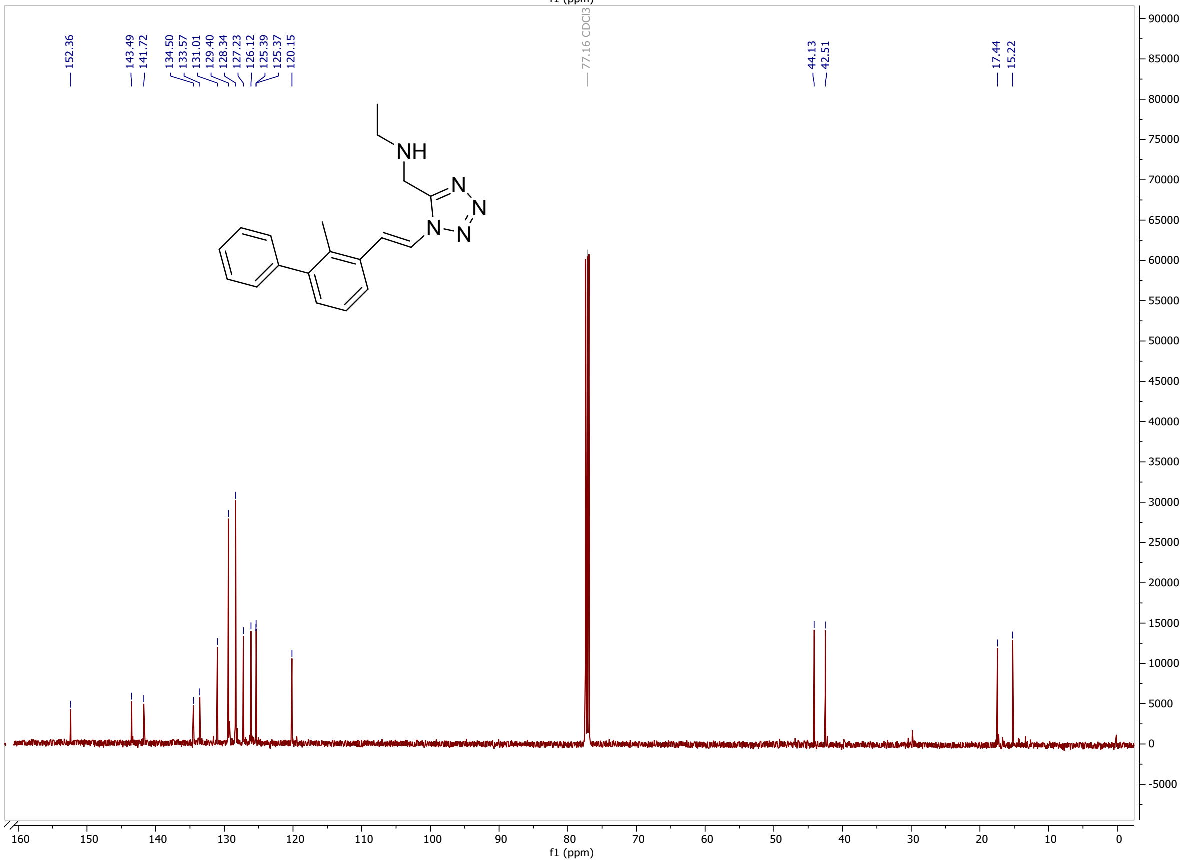

(E)-3-((1-(1-(2-(2-methyl-[1,1'-biphenyl]-3-yl)vinyl)-1H-tetrazol-5-yl)ethyl)amino)propan-1-ol (10g)

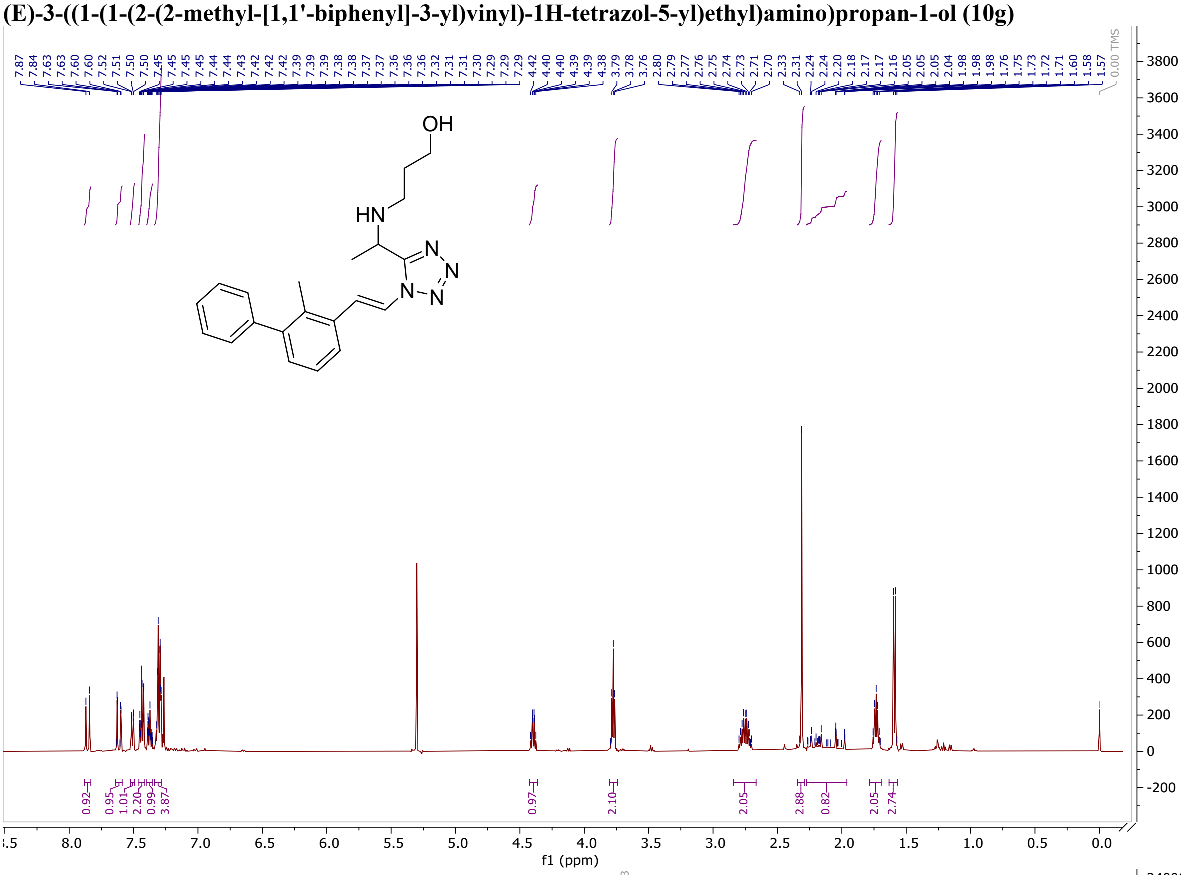

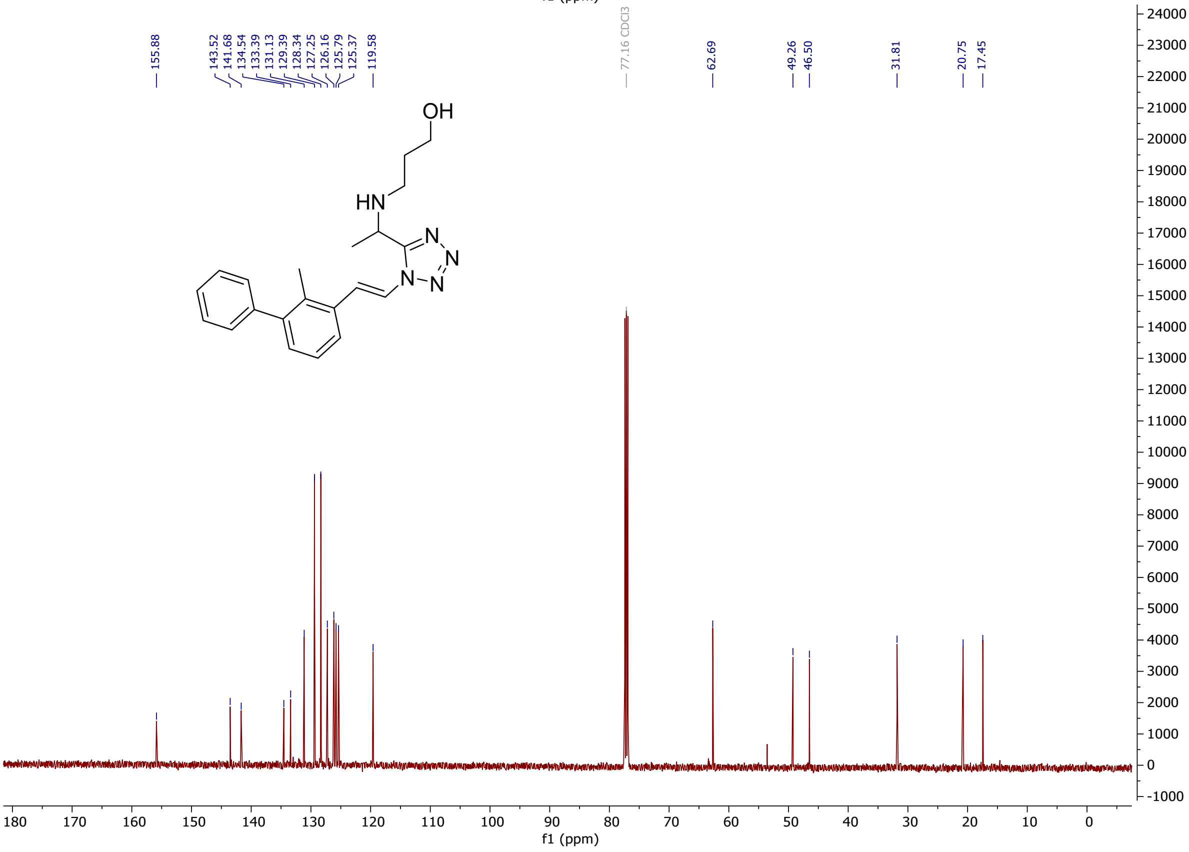

(1r,3r)-3-(((1-((E)-2-(2-methyl-[1,1'-biphenyl]-3-yl)vinyl)-1H-tetrazol-5-yl)methyl)amino)adamantan-1-ol (10h)

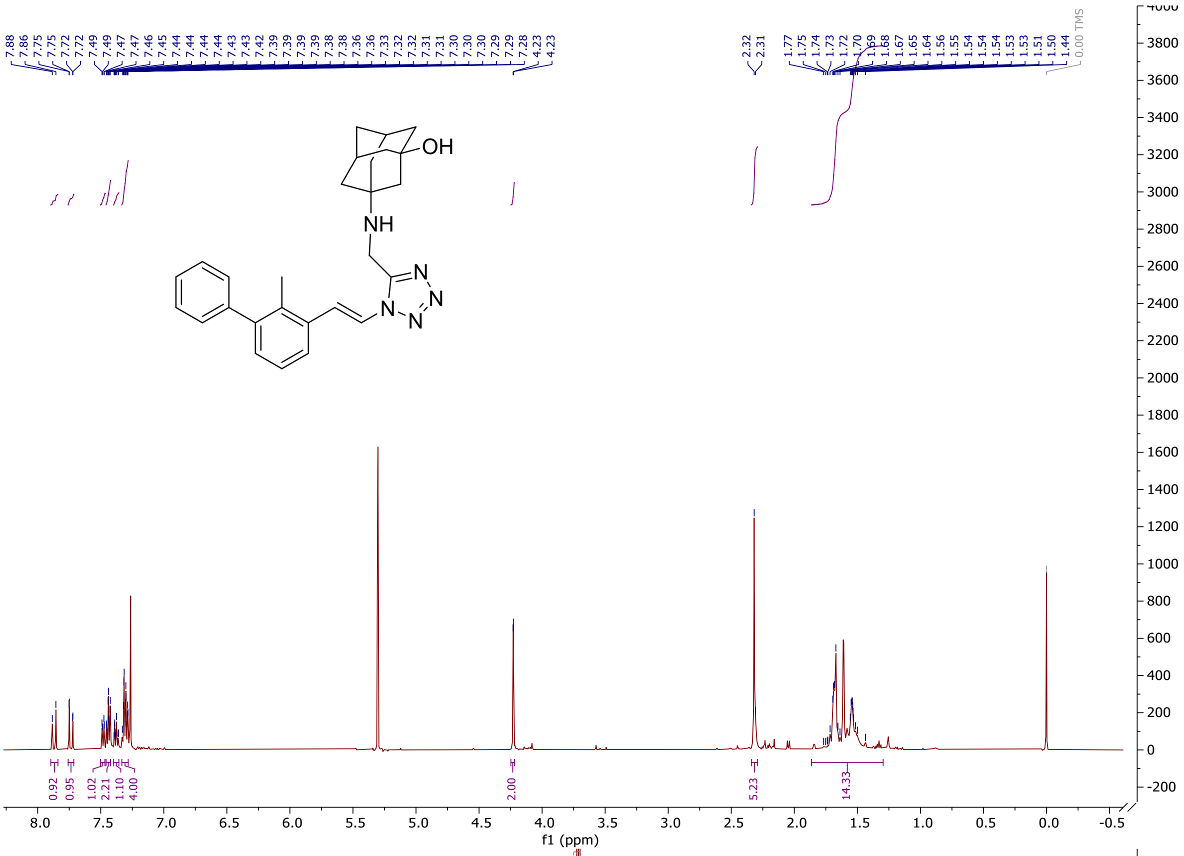

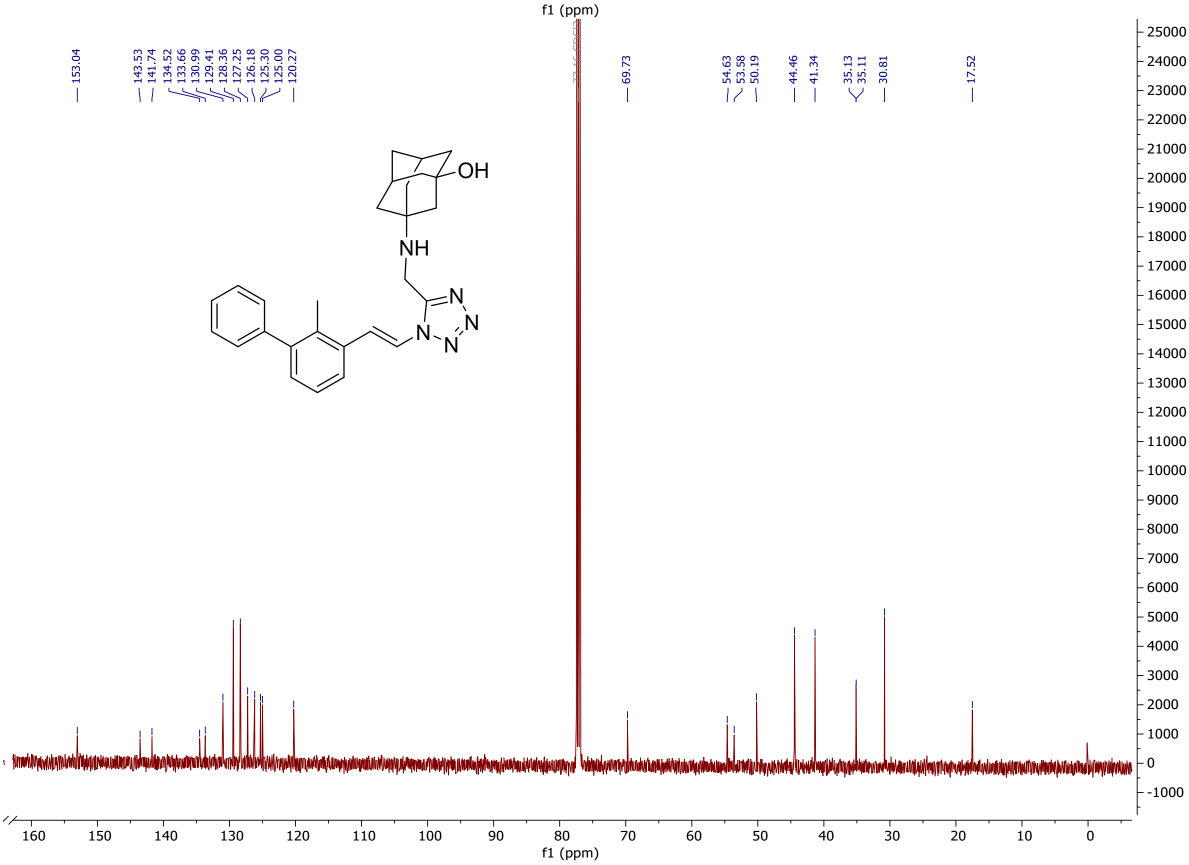

(E)-3-((1-(2-(2-methyl-[1,1'-biphenyl]-3-yl)vinyl)-1H-tetrazol-5-yl)methyl)-1,3-oxazepane (10i)

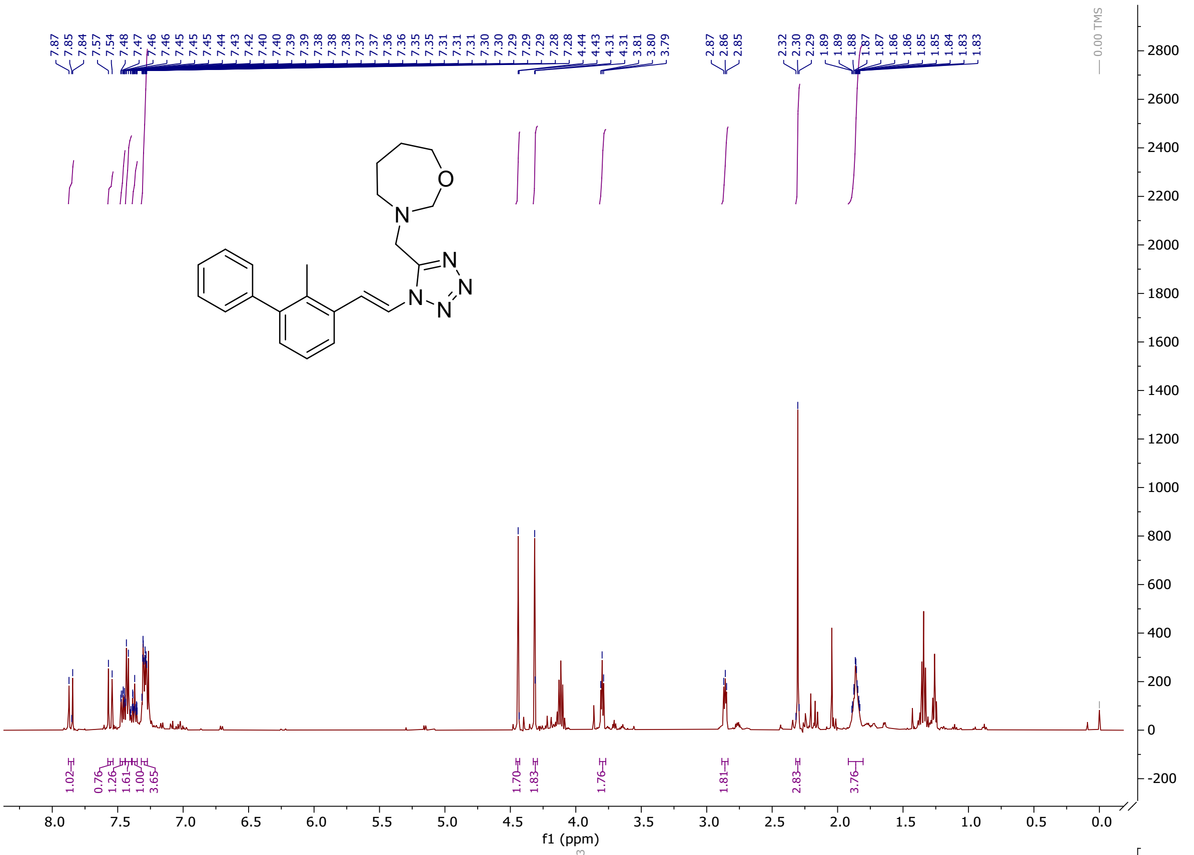

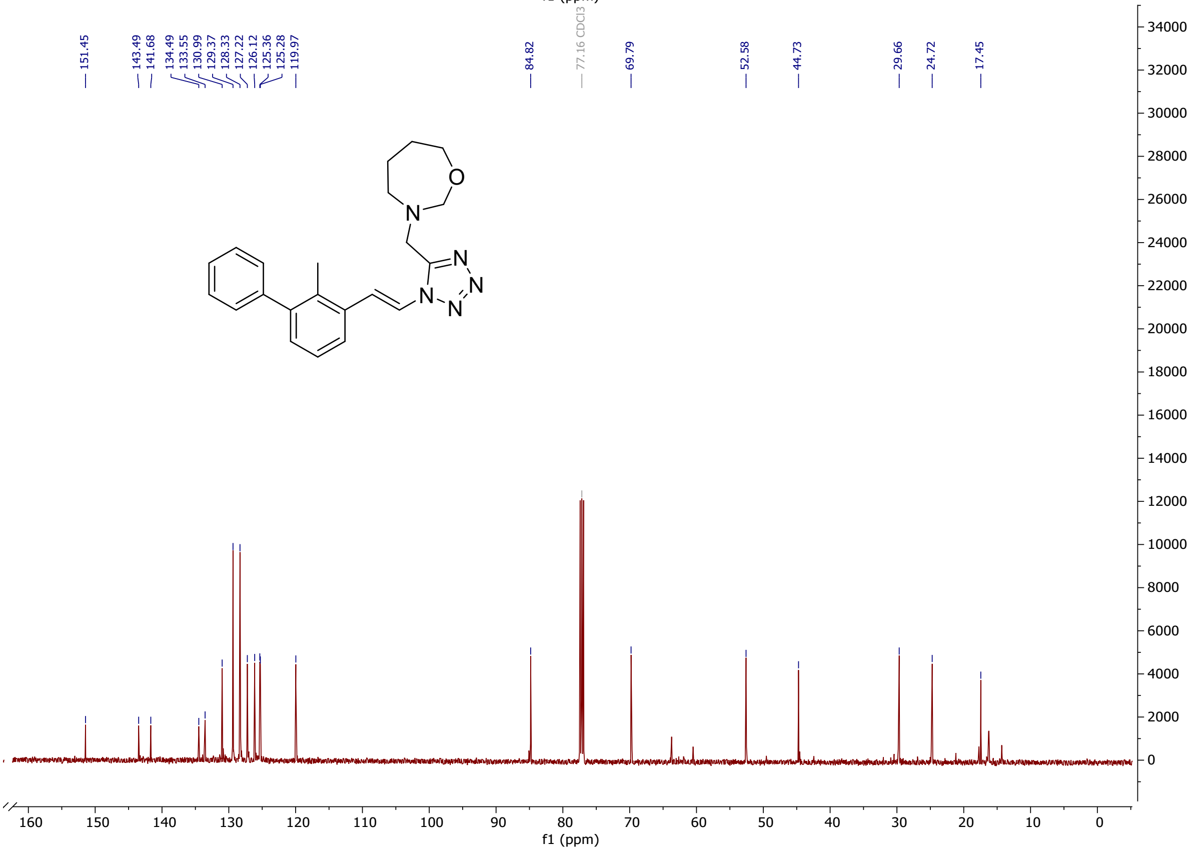

(E)-5-(((1-(2-(2-methyl-[1,1'-biphenyl]-3-yl)vinyl)-1H-tetrazol-5-yl)methyl)amino)pentan-1-ol (10j)

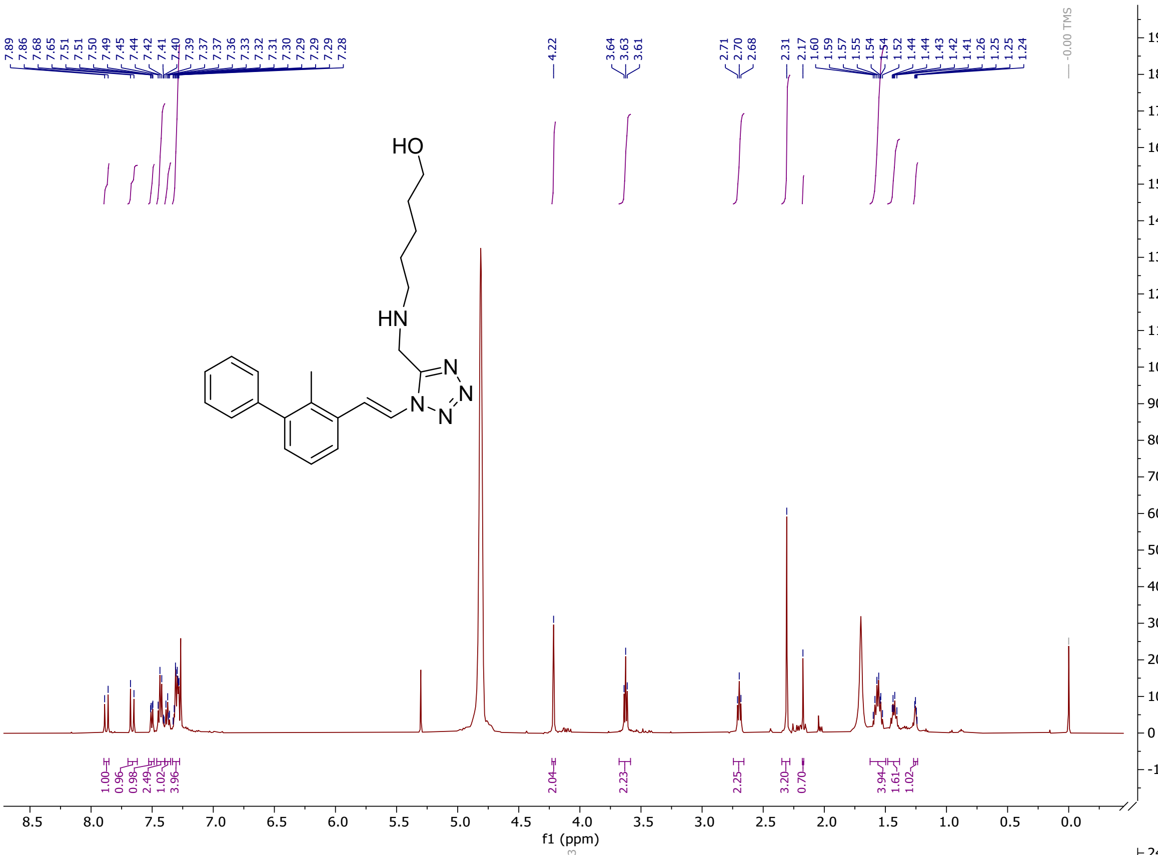

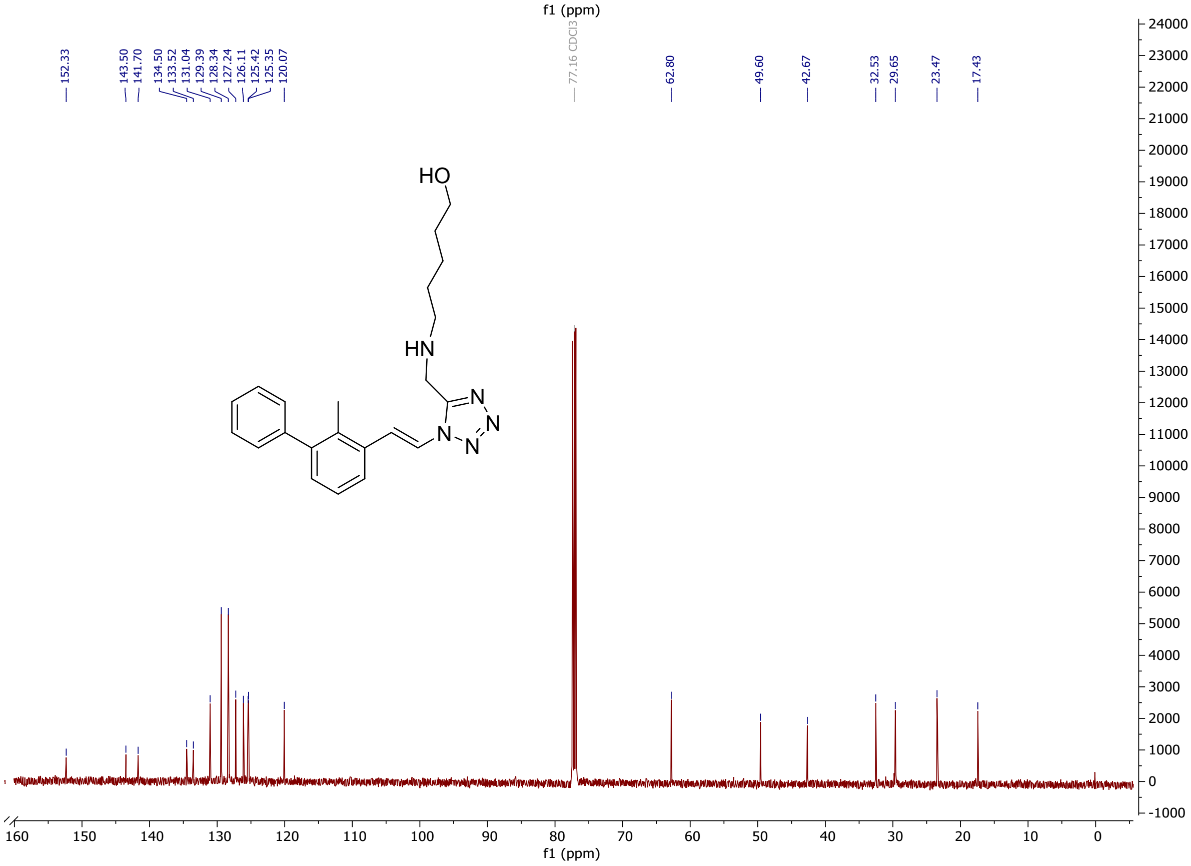

(E)-3-((1-(2-(2-methyl-[1,1'-biphenyl]-3-yl)vinyl)-1H-tetrazol-5-yl)methyl)oxazolidine (10k)

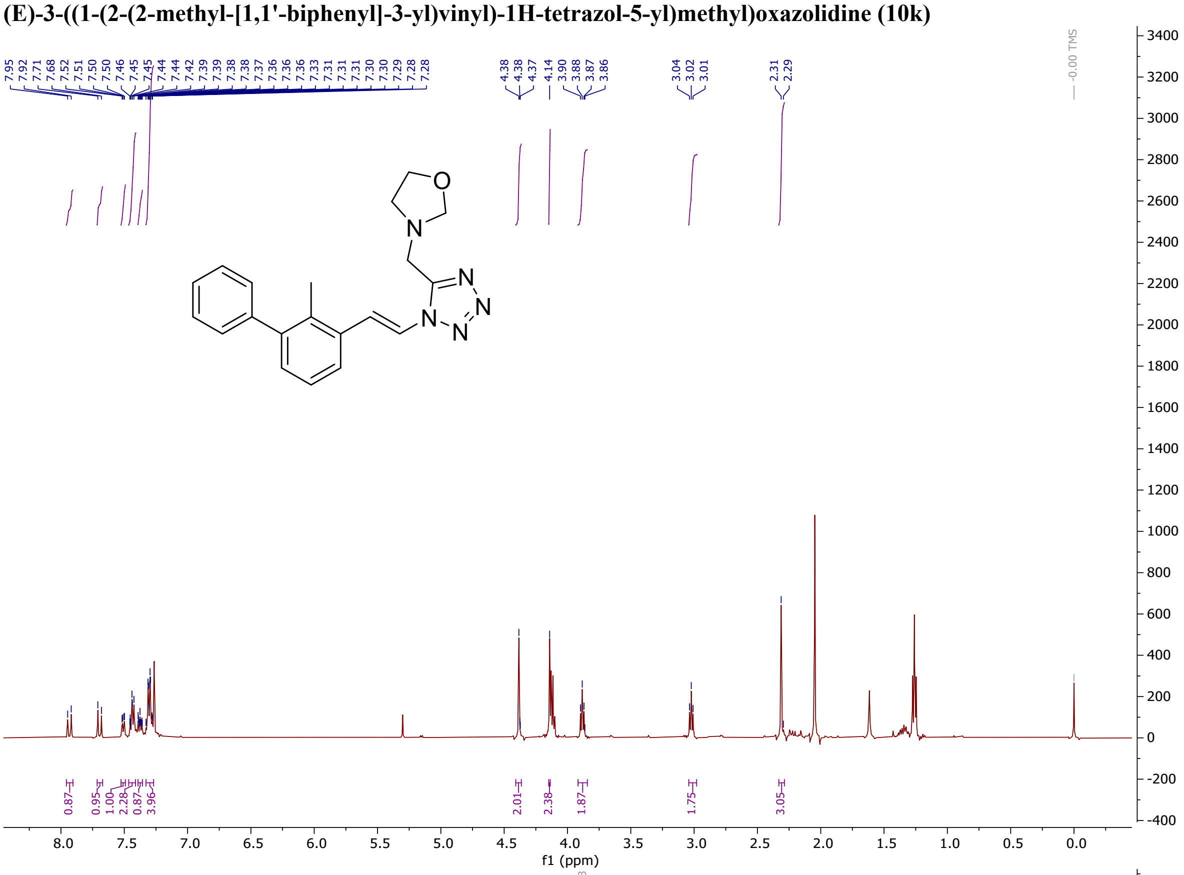

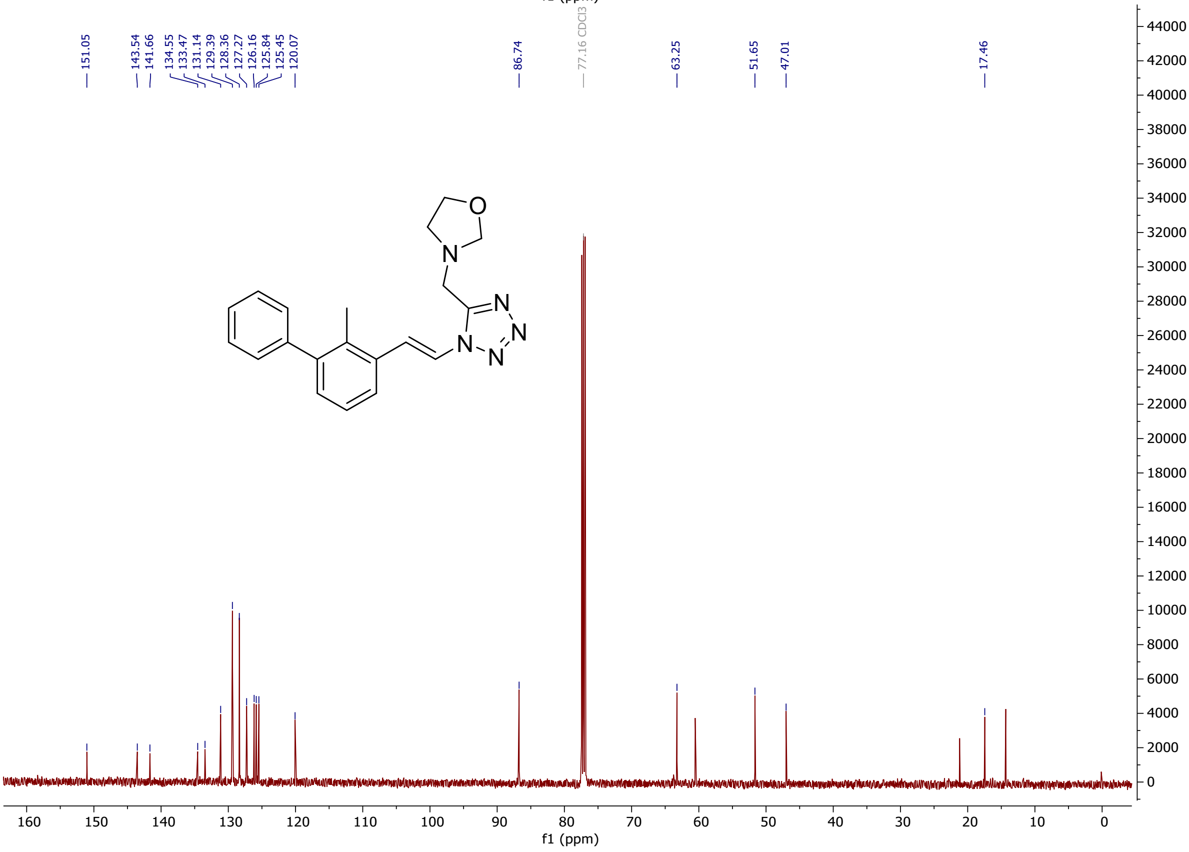

(E)-3-((1-(2-(2-methyl-[1,1'-biphenyl]-3-yl)vinyl)-1H-tetrazol-5-yl)methyl)-1,3-oxazinane (10l)

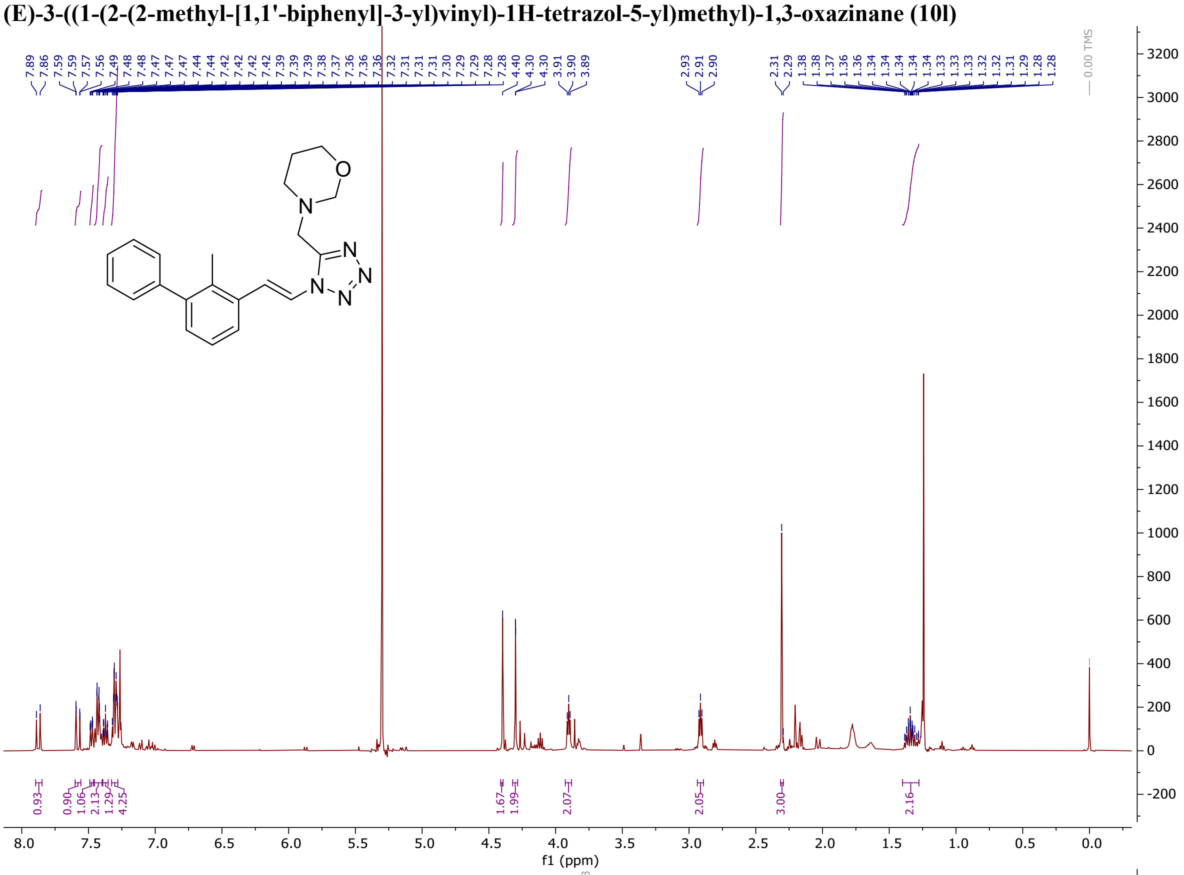

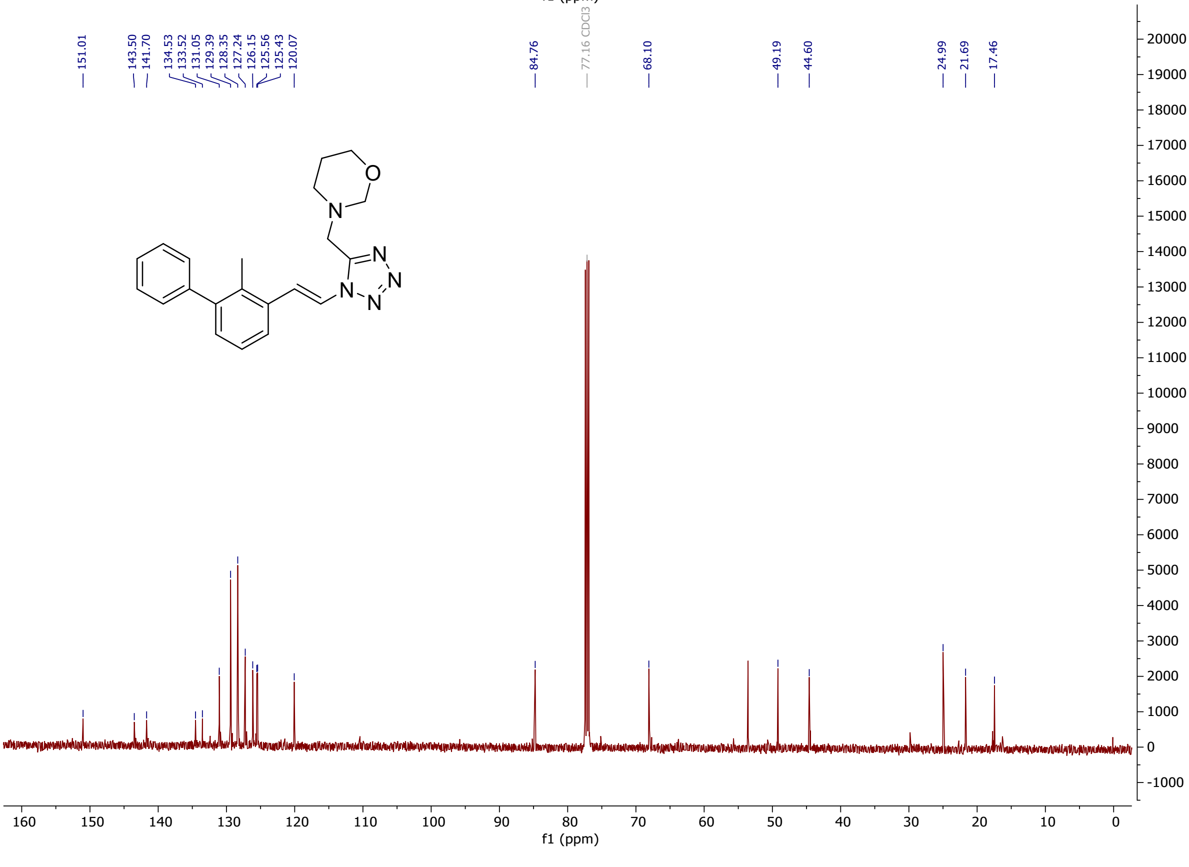

(1r,3r)-3-(((1-((E)-3-(2,3-dihydrobenzo[b][1,4]dioxin-6-yl)-2-methylstyryl)-1H-tetrazol-5-yl)methyl)amino)adamantan-1-ol (10m)

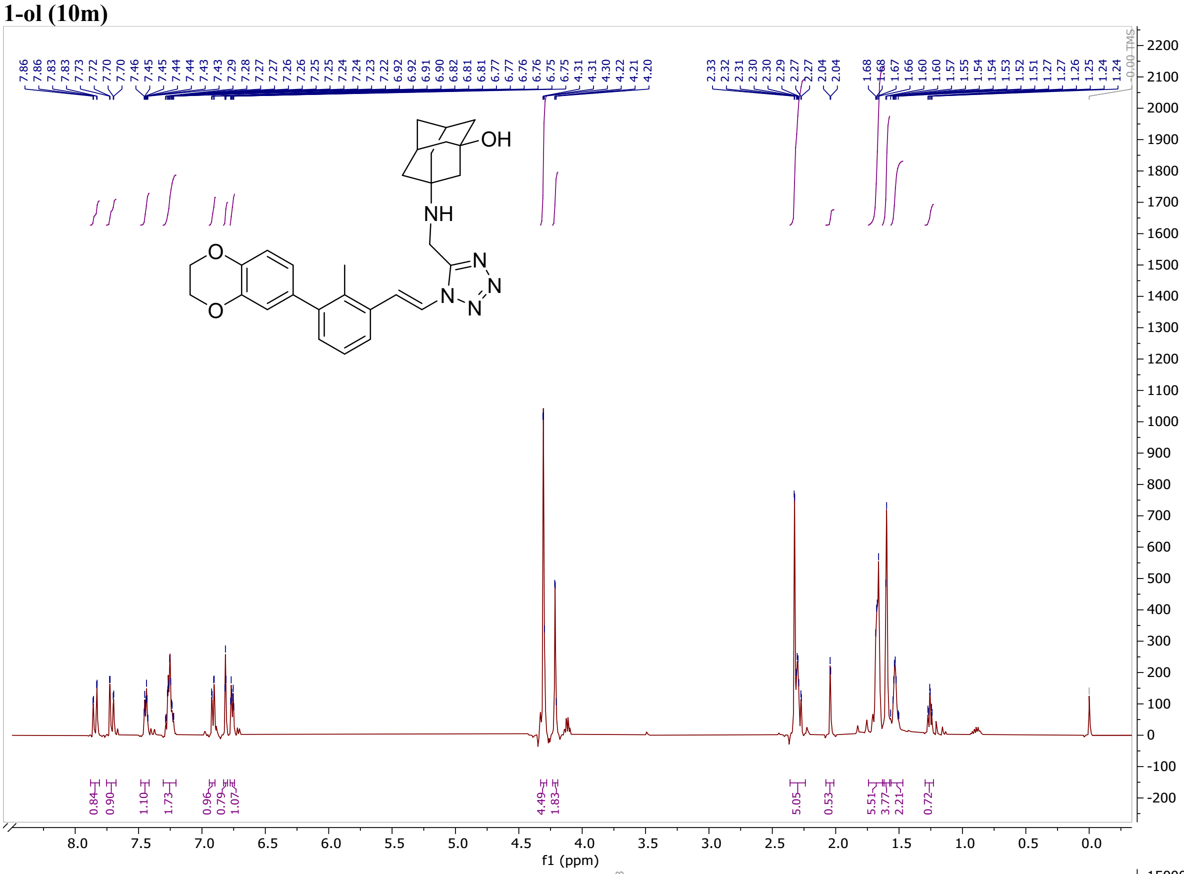

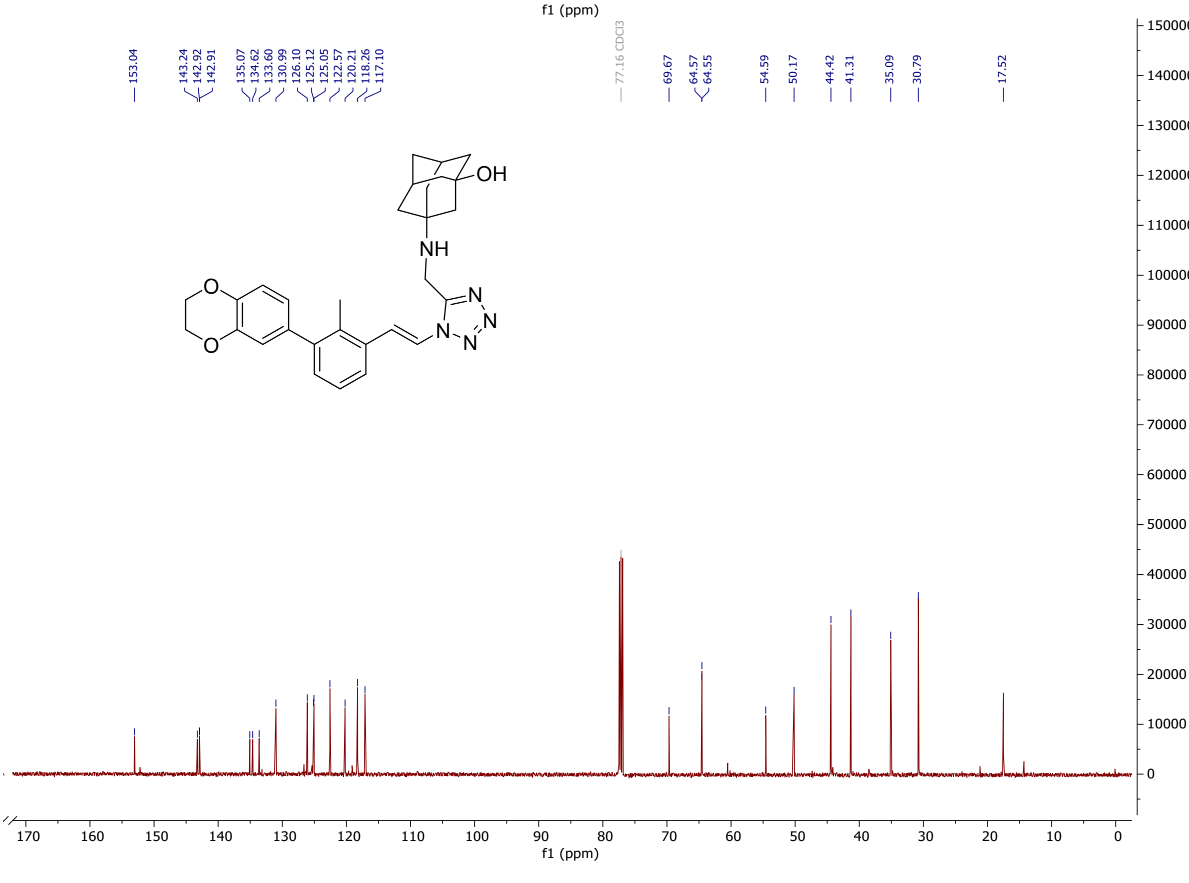

(E)-5-(((1-(3-(2,3-dihydrobenzo[b][1,4]dioxin-6-yl)-2-methylstyryl)-1H-tetrazol-5-yl)methyl)amino)pentan-1-ol (10n)

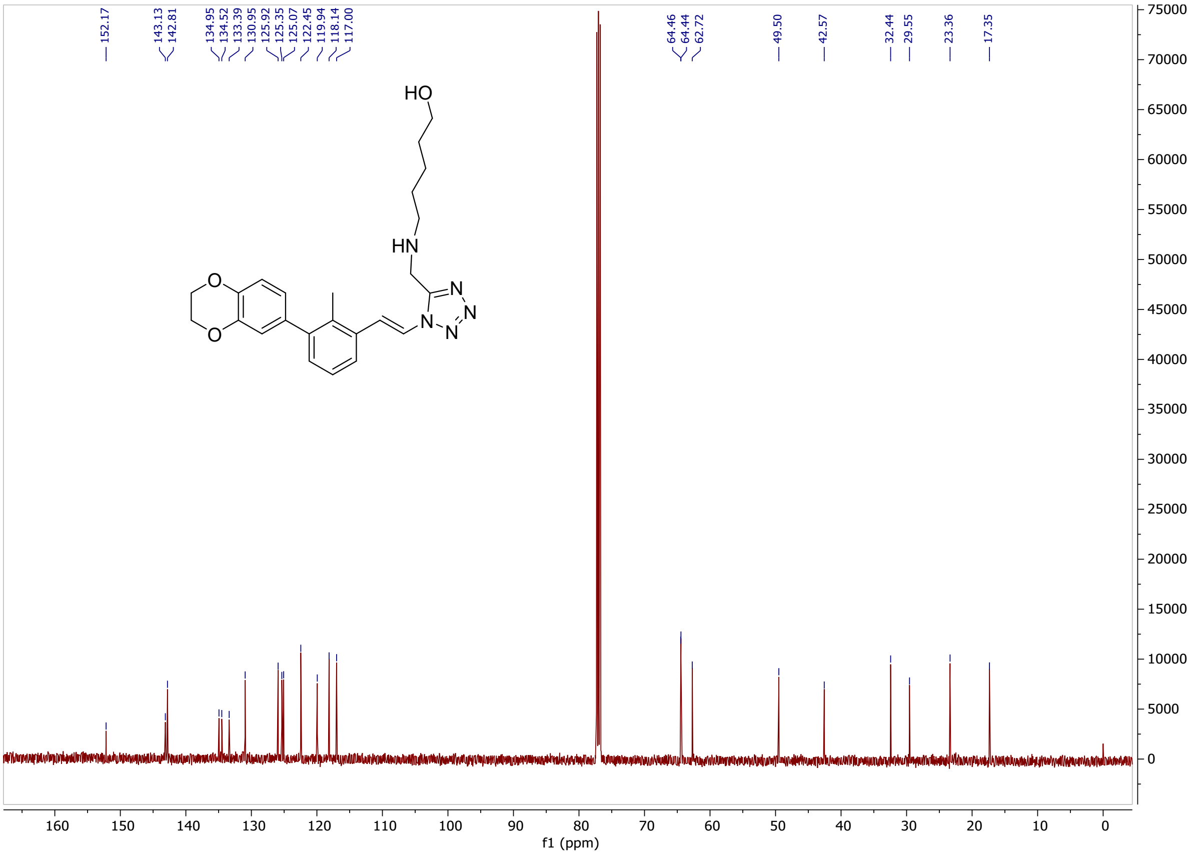

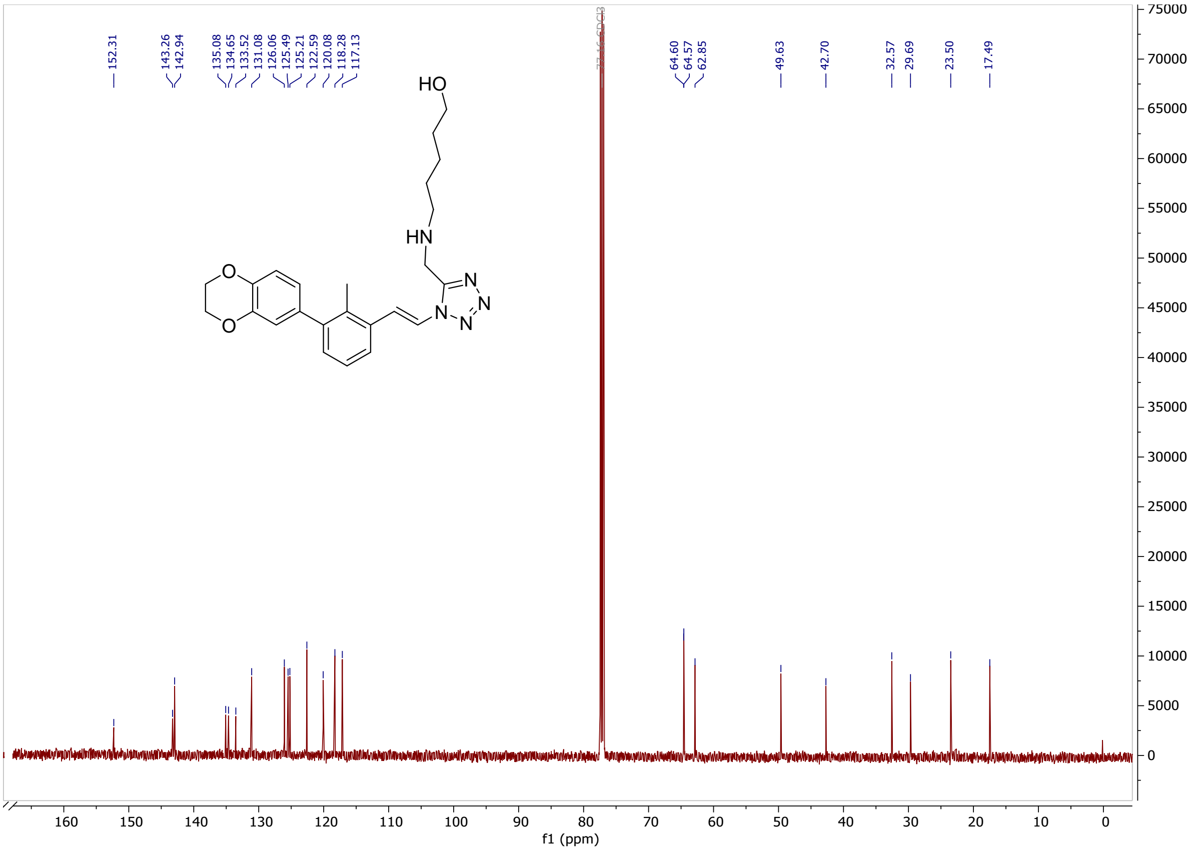

(E)-4-(((1-(3-(2,3-dihydrobenzo[b][1,4]dioxin-6-yl)-2-methylstyryl)-1H-tetrazol-5-yl)methyl)amino)butan-1-ol (10o)

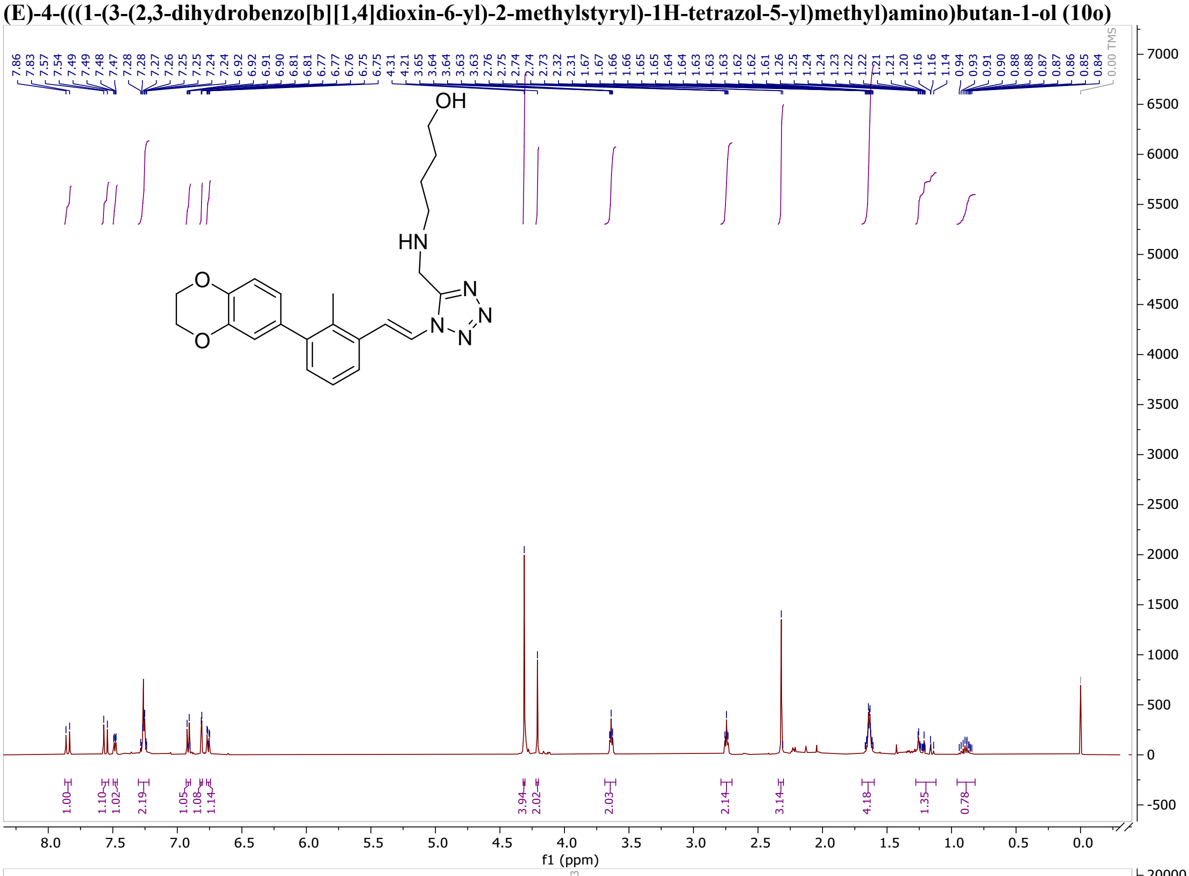

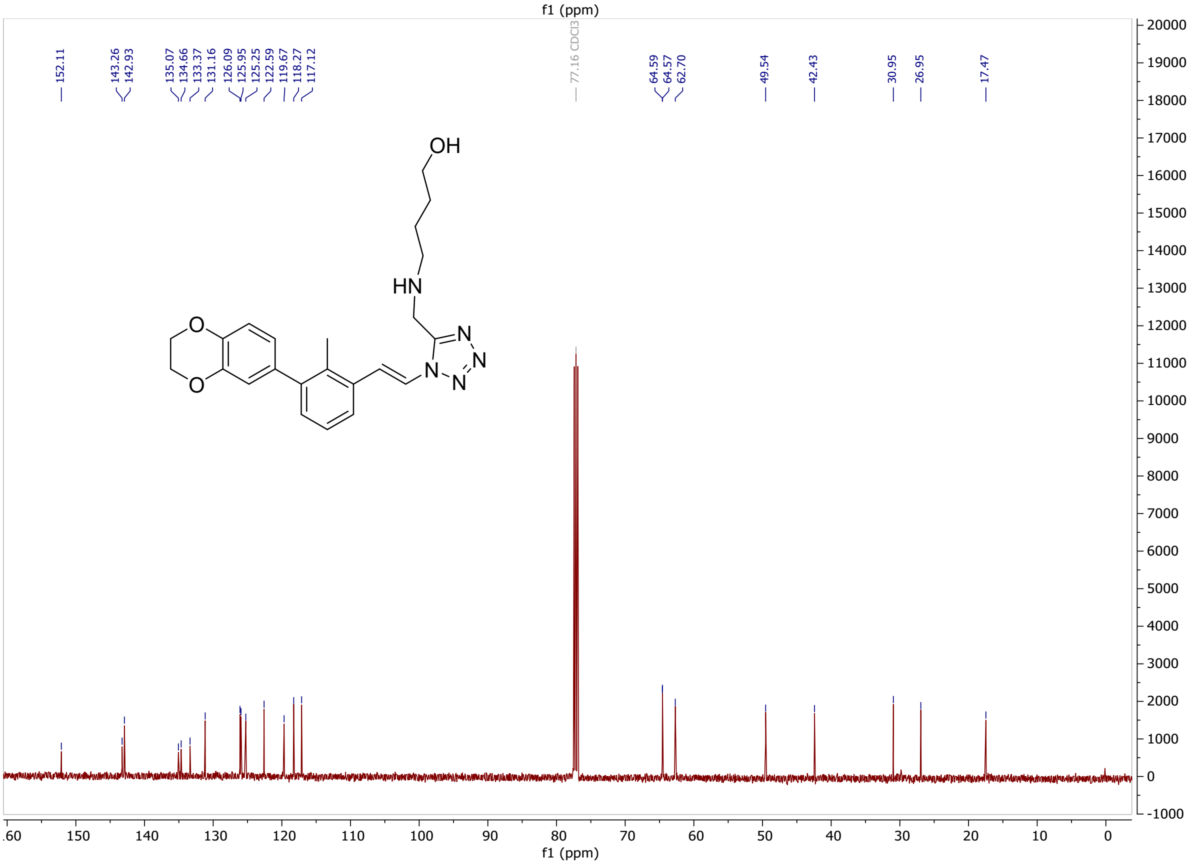

(E)-3-((1-(1-(3-(2,3-dihydrobenzo[b][1,4]dioxin-6-yl)-2-methylstyryl)-1H-tetrazol-5-yl)ethyl)amino)propan-1-ol (10p)

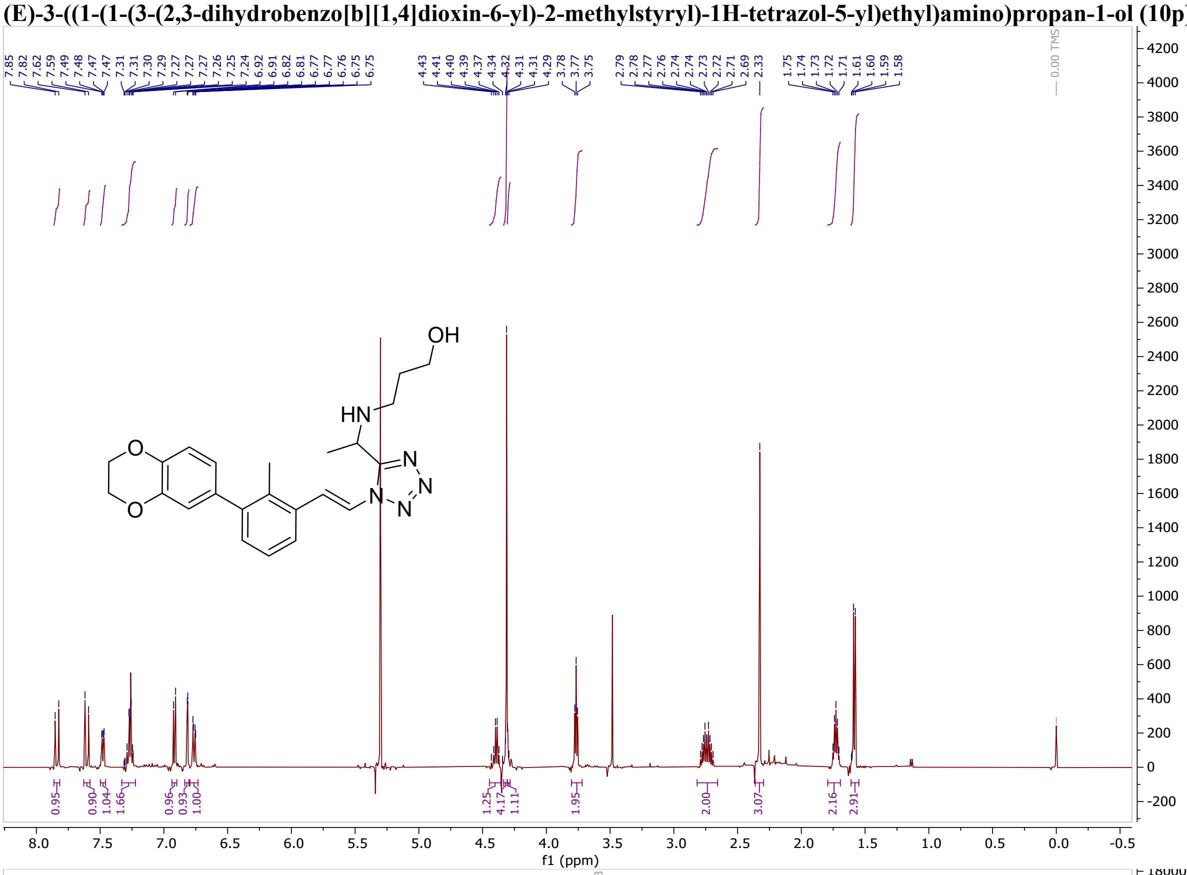

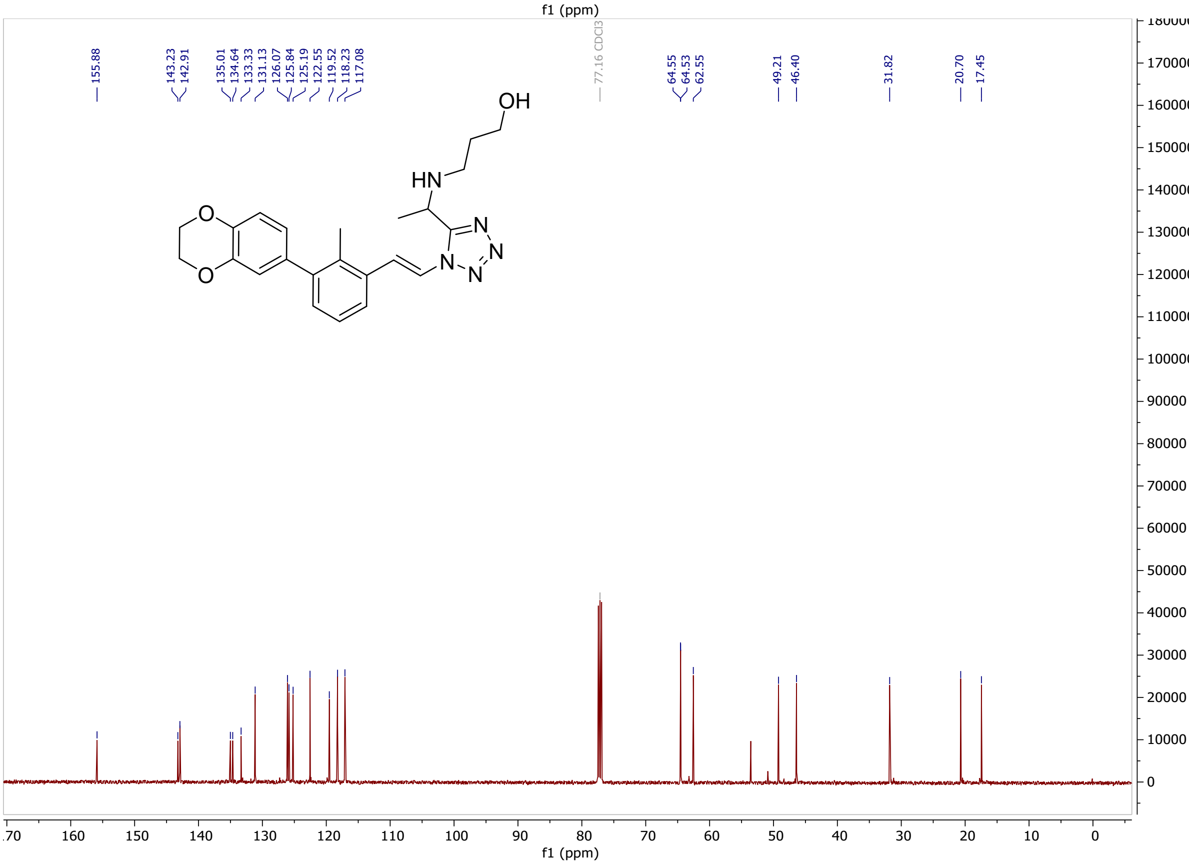

(E)-2-(((1-(3-(2,3-dihydrobenzo[b][1,4]dioxin-6-yl)-2-methylstyryl)-1H-tetrazol-5-yl)methyl)amino)ethan-1-ol (10q)

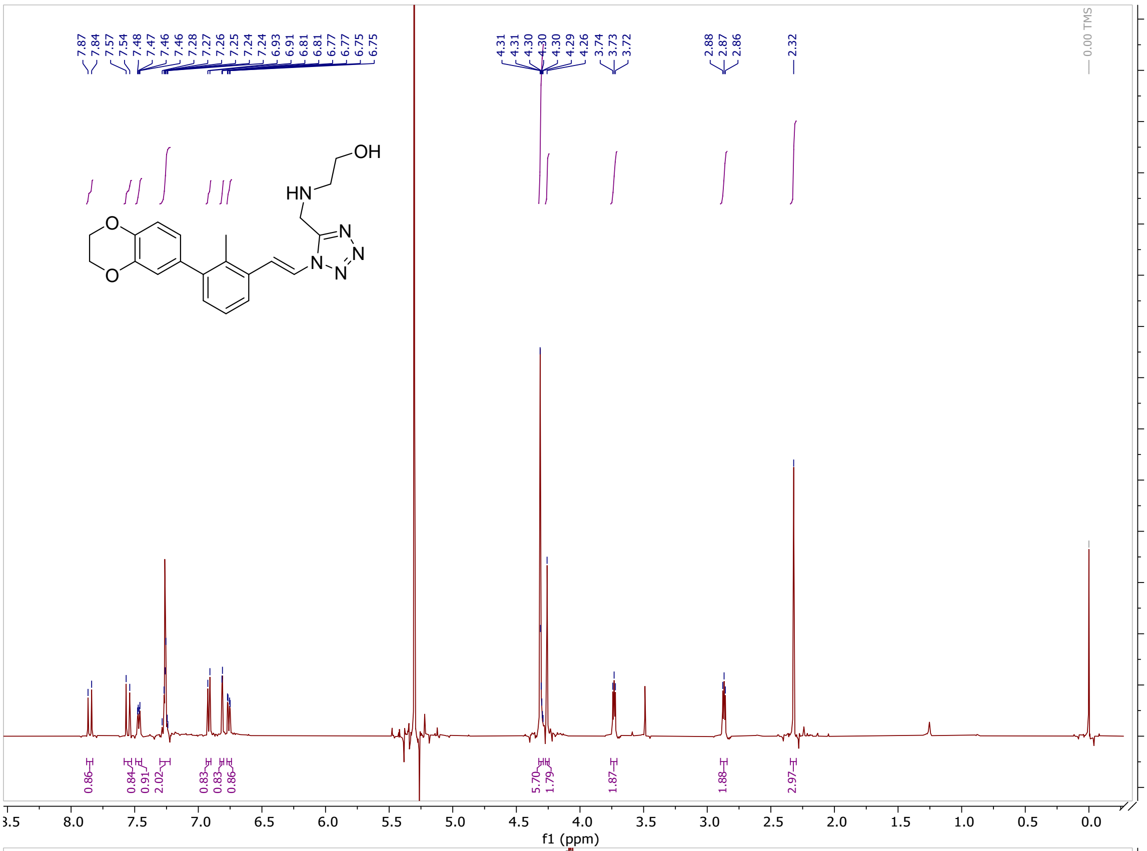

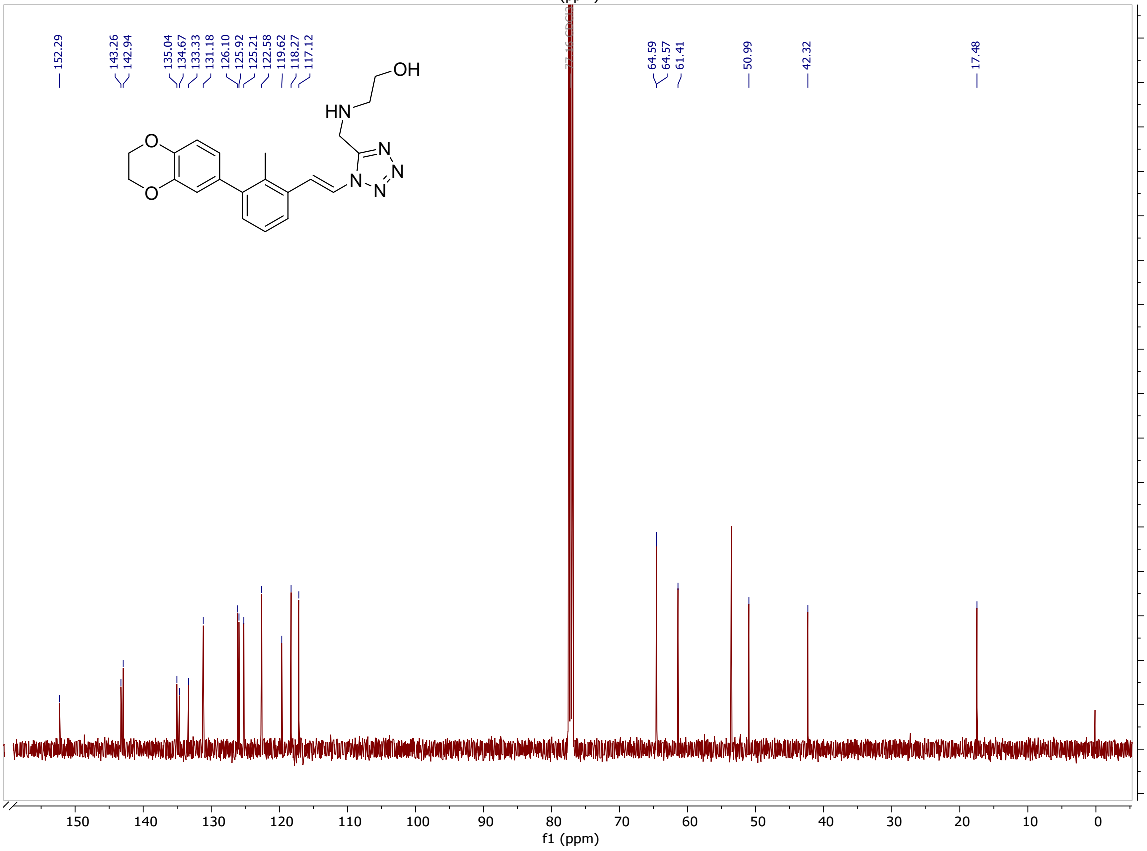

(E)-3-((1-(3-(2,3-dihydrobenzo[b][1,4]dioxin-6-yl)-2-methylstyryl)-1H-tetrazol-5-yl)methyl)-1,3-oxazinane (10r)

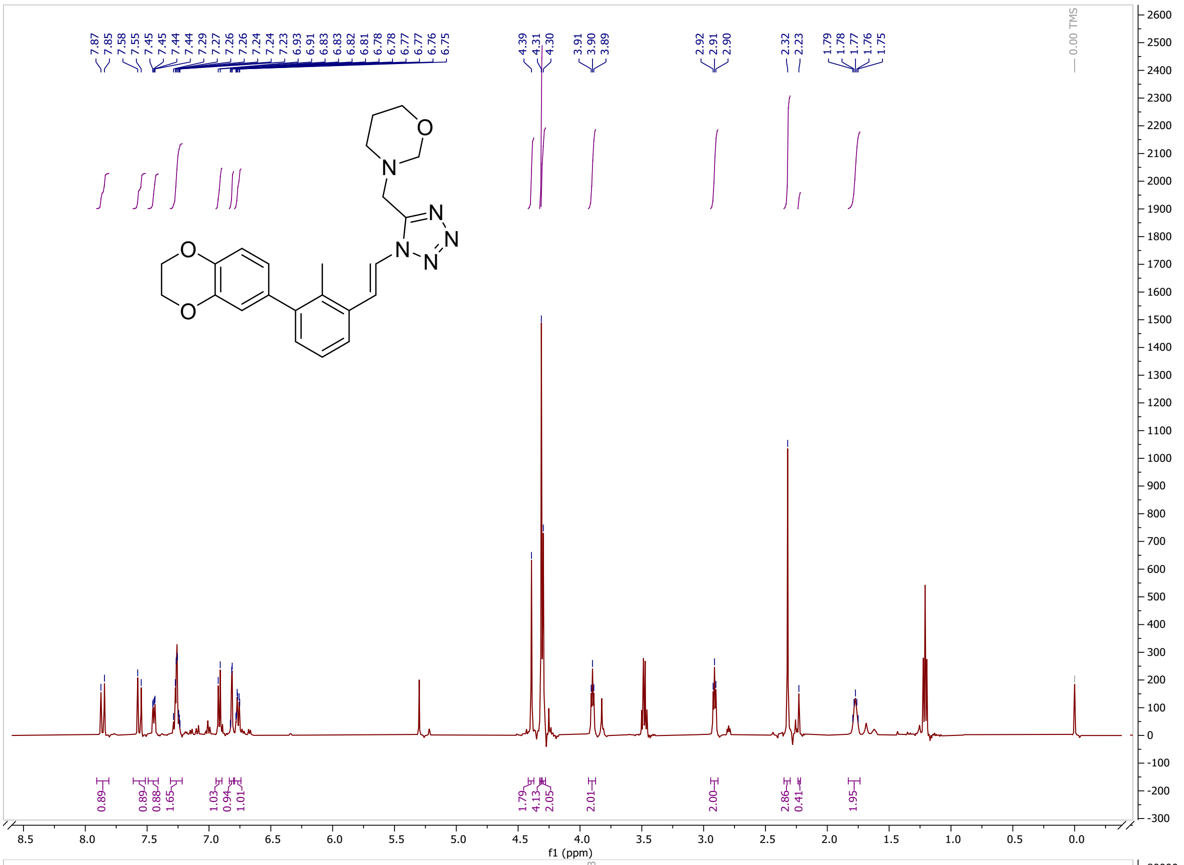

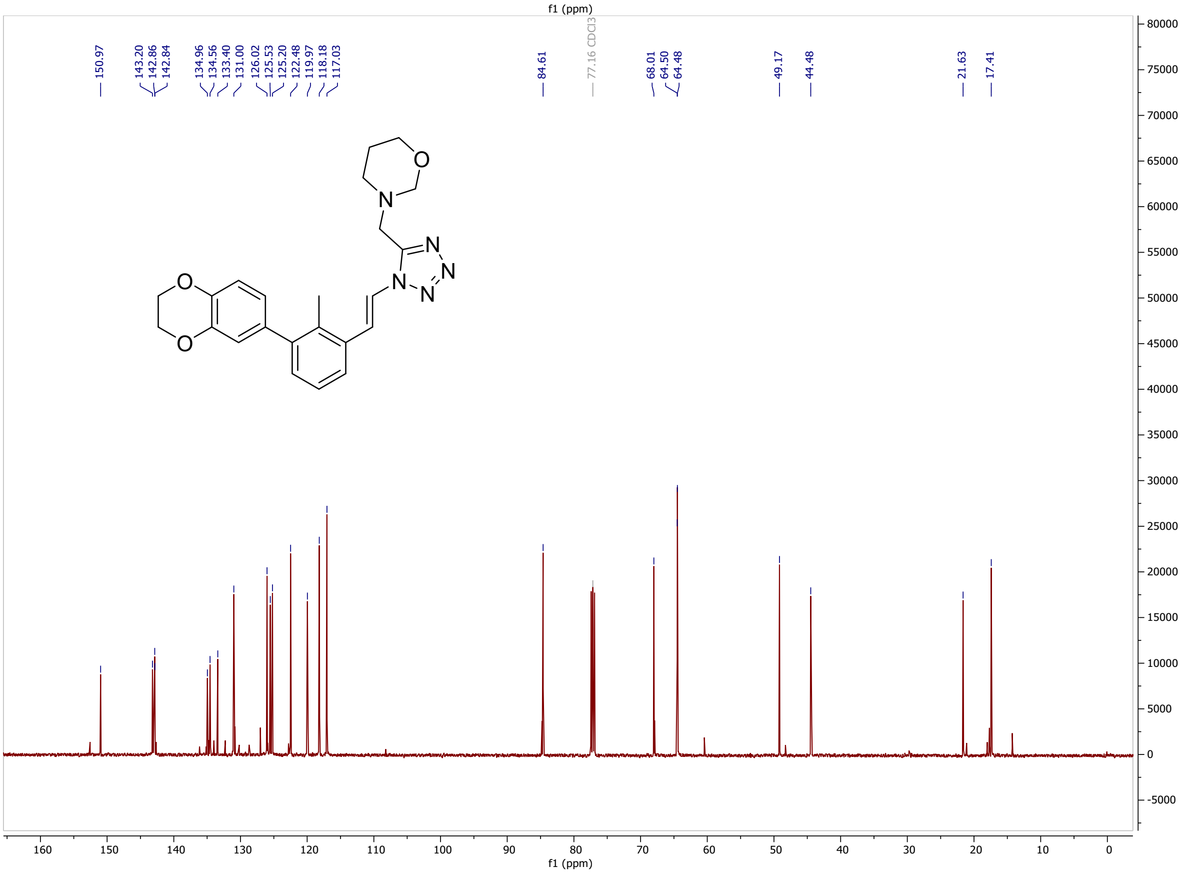

NMR binding assay

NMR spectra were recorded in PBS pH 7.4 containing 10% (v/v) of D_2O added to the samples to provide the lock signal. Water suppression was carried out using the WATERGATE pulse sequence. All the spectra were recorded at 300 K using a Bruker Avance III 600 MHz spectrometer. The spectra were recorded at the protein/ligand at the molar ratio 1:1 and 1:10. The sample were prepared by adding the appropriate amounts of inhibitor, from 50 mM ligand stock solution in DMSO-d_6, to the protein solution of PD-L1 at a concentration of 0.2 mM. Spectra were visualized using *TopSpin 3.2*

Protein expression and purification

E. coli strain BL21 was transformed with pET-21b plasmid carrying PD-L1 gene (amino acids 18-134). The bacteria were cultured in LB at 37°C until OD_{600nm} of 0.6 when the recombinant protein production was induced with 1 mM IPTG. The protein production was continued overnight. Inclusion bodies were collected by centrifugation, washed twice with 50 mM Tris-HCl pH 8.0 containing 200 mM NaCl, 10 mM EDTA, 10 mM 2-mercaptoethanol and 0.5% Triton X-100, followed by a single wash with the same buffer but with no Triton X-100. The washed inclusion bodies were resuspended overnight in 50 mM Tris-HCl pH 8.0, 6 M GuHCl, 200 mM NaCl and 10 mM 2-mercaptoethanol and clarified with centrifugation. Refolding of PD-L1 was performed by drop-wise dilution into 0.1 M Tris-HCl pH 8.0 containing 1 M L-arginine hydrochloride, 0.25 mM oxidized glutathione and 0.25 mM reduced glutathione. The refolded protein was dialyzed 3 times against 10 mM Tris-HCl pH 8.0 containing 20 mM NaCl and purified by size exclusion chromatography using Superdex 75 and PBS pH 7.4. The quality of the refolded protein was evaluated by SDS-PAGE and NMR.

PD-L1 cocrystalization

Purified PD-L1 was concentrated to 5 mg/ml, mixed with the inhibitor in 1:3 molar ratio (protein:compound) and clarified by centrifugation at 15 000 × g for 10 min. Supernatant was used for screening using a sitting-drop vapor diffusion method and commercially available buffer sets. Diffraction-quality crystals were obtained at room temperature from the condition containing: 1.2 M S38 sodium citrate tribasic dihydrate 0.01 M sodium borate, pH 8.5. The crystal was flash-cooled in liquid nitrogen.

Crystal structure determination and refinement

The X-ray diffraction data were collected at the BESSY II (Berlin Adlershof, Germany). The data were indexed, integrated, and scaled using XDS, XSCALE, and Aimless (Evans & Murshudov, 2013; Kabsch & IUCr, 2010; Krug et al., 2012). Dataset was then processed in CCP4 cloud (Krissinel et al., 2018). Initial phases were obtained by molecular replacement calculated in Phaser 2.8.3 (McCoy et al., 2007). using PDB ID: 5C3T experimental model. The model building was performed in Coot (Emsley et al., 2010) and refinement was performed using Refmac5 (Murshudov et al., 2011). Coordinates and structure factors were deposited in the Protein Data Bank under accession code PDB: 8P64.

Table S1 Data collection and refinement statistics

Data collection	
Wavelength [Å]	0.9184
Space group	P 31 2 1 (152)
Unit cell parameters [Å] and angles	73.45 73.45 96.08<br>90.0 90.0 120.0
Resolution limit [Å]	48.04-3.31 (3.51-3.31)
No. of reflections	50033 (7778)
No. of uniques	4743 (743)
Multiplicity	10.55 (10.47)
I/σI	9.39 (0.96)
R_meas [%]	25.8 (292.0)
Completeness [%]	99.9 (99.9)
B(Wilson) [A^2]	86.42
Mosaicity [deg]	0.180
CC(1/2)	99.7 (42.3)
ISa	20.05
Refinement statistics	
Rwork	0.2340
Rfree	0.2970
FSC average	0.9440
RMSD bonds	0.0076
RMSD angles	1.7440
Ramachandran favoured (%)	95.2
Ramachandran allowed (%)	3.9
Ramachandran outliers (%)	0.9
Rotamer outliers	10.2
Clash score	5.8
MolProbity score	2.41

*high resolution shell parameters in the parentheses.

Homogenous Time Resolved Fluorescence

HTRF was performer using the certified Cis-Bio assay according to the manufacturer's guidelines. The experiments were performed at 5 nM of hPD-L1 and 50 nM of hPD-1 in the final formulation at 20 µL final volume in the well. After mixing all components according to Cis-Bio protocol, the plate was left for 1h incubation at room temperature followed by TR-FRET measurement on Tecan Spark 20M. To determine the activity of tested compounds data was collected for 2 different concentrations of the ligand: 50 nM and 5 nM in the final volume. For the several active compounds (**10f**, **10l**, **10p**) half maximal inhibitory concentration (IC_{50}) was measured with 6 different concentrations of the ligand in the final volume. All measurements were performed on two individual dilution series, unless states otherwise. Collected data was background subtracted on the negative control, normalized on the positive control, averaged and for IC_{50} determination fitted with normalized Hill's equation to determine the IC_{50} value using *Mathematica 12*. For remaining compounds, which were measured in scouting mode, the inhibitory constants were approximated by translation of the average Hill's fits of fully described compounds to match the experimental datapoints. Since all compounds reported here are closely related, the slope of Hill's fit that determines the shape of the curve is therefore similar across them. The estimation was performed using *Mathematica 12.*

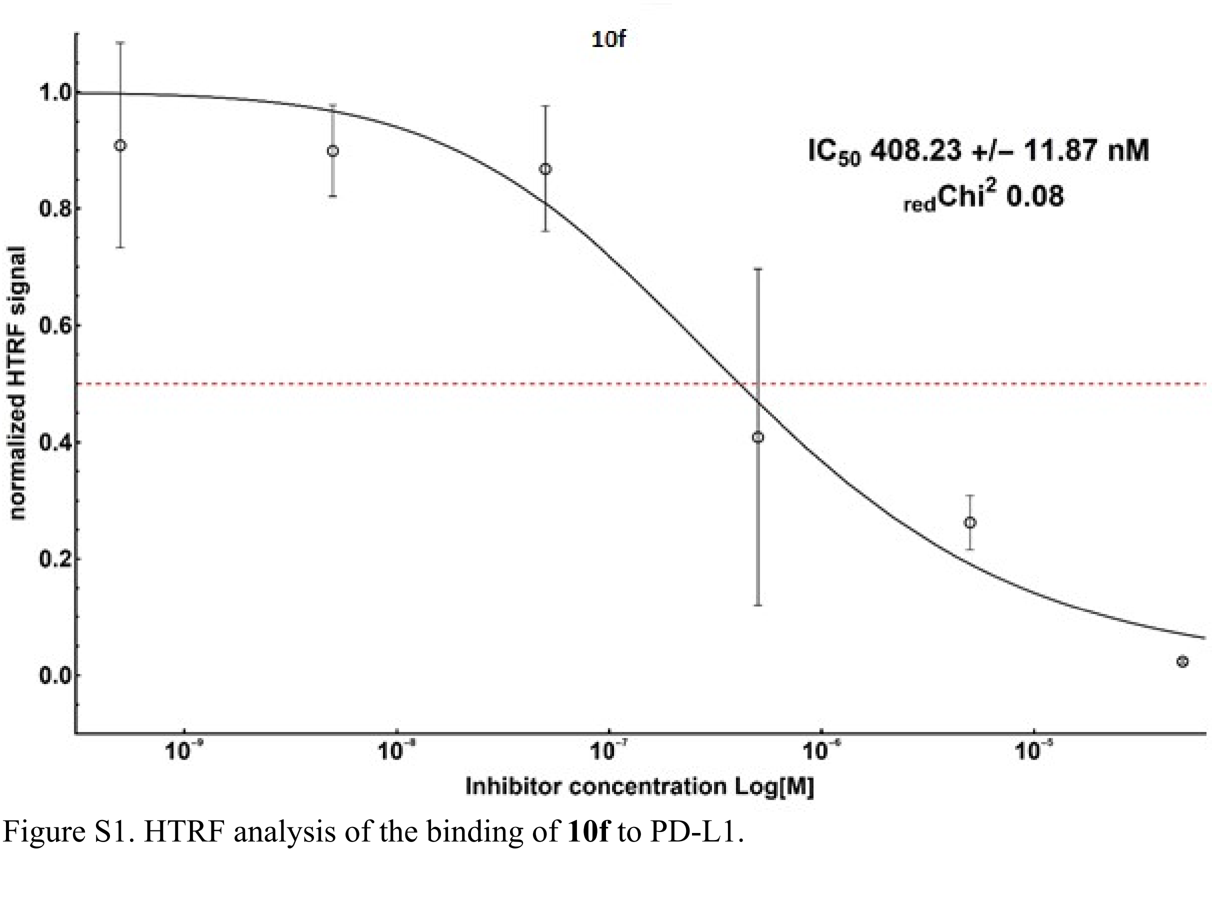

Figure S1. HTRF analysis of the binding of **10f** to PD-L1.

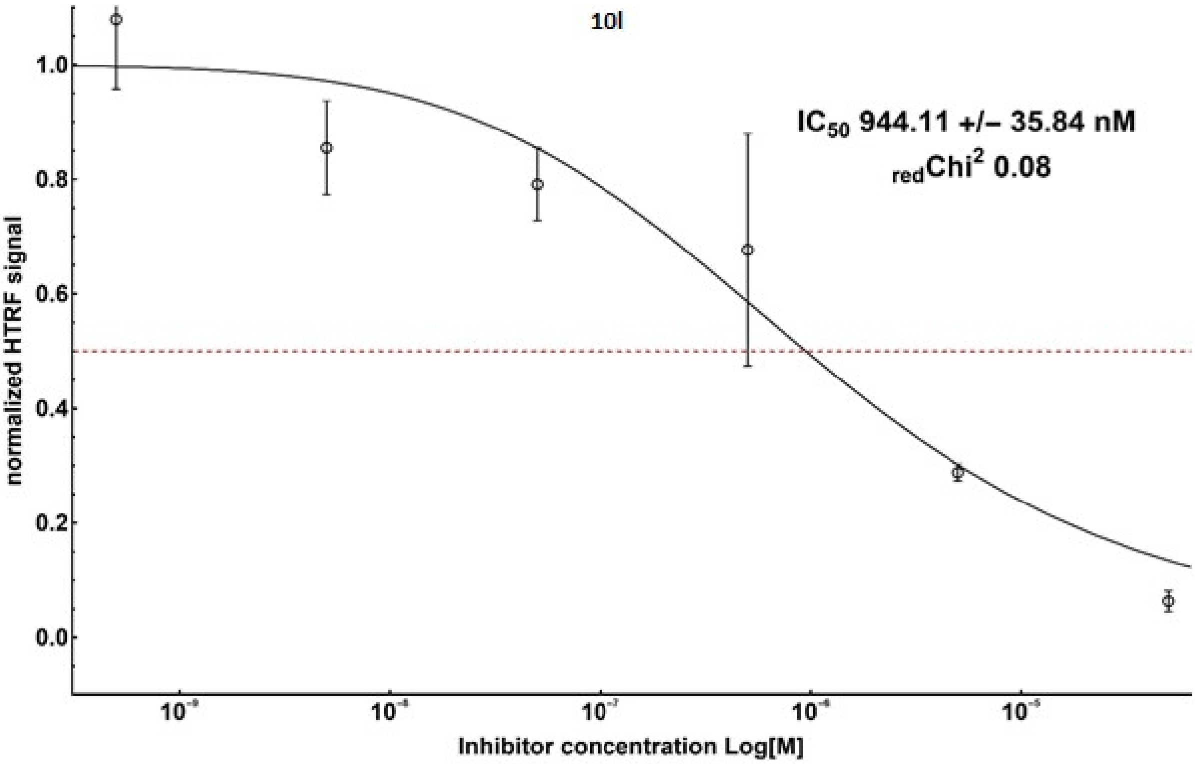

Figure S2. HTRF analysis of the binding of **10l** to PD-L1.

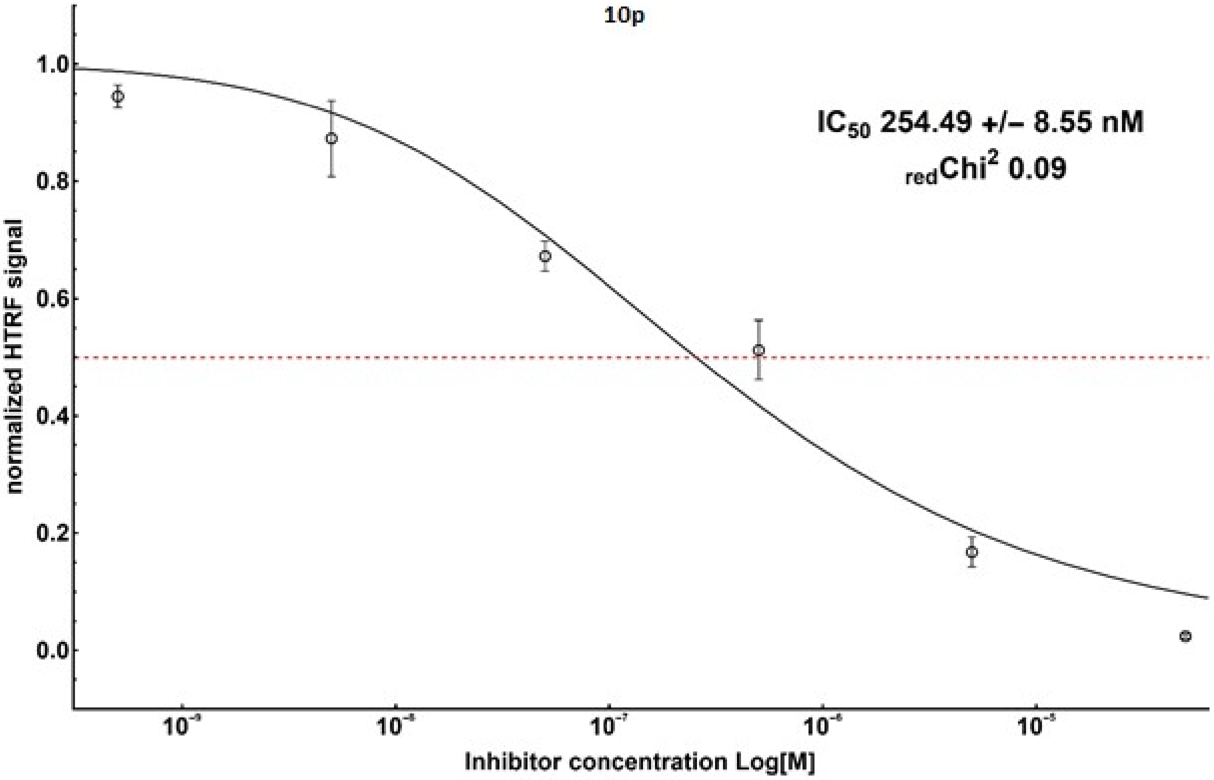

Figure S3. HTRF analysis of the binding of **10p** to PD-L1.

References:

Emsley, P., Lohkamp, B., Scott, W. G., & Cowtan, K. (2010). Features and development of Coot. *Urn:Issn:0907-4449*, *66*(4), 486–501. https://doi.org/10.1107/S0907444910007493

Evans, P. R., & Murshudov, G. N. (2013). How good are my data and what is the resolution? *Urn:Issn:0907-4449*, *69*(7), 1204–1214. https://doi.org/10.1107/S0907444913000061

Kabsch, W., & IUCr. (2010). XDS. *Urn:Issn:0907-4449*, *66*(2), 125–132. https://doi.org/10.1107/S0907444909047337

Krissinel, E., Uski, V., Lebedev, A., Winn, M., & Ballard, C. (2018). Distributed computing for macromolecular crystallography. *Urn:Issn:2059-7983*, *74*(2), 143–151. https://doi.org/10.1107/S2059798317014565

Krug, M., Weiss, M. S., Heinemann, U., & Mueller, U. (2012). XDSAPP: a graphical user interface for the convenient processing of diffraction data using XDS. *Urn:Issn:0021-8898*, *45*(3), 568–572. https://doi.org/10.1107/S0021889812011715

McCoy, A. J., Grosse-Kunstleve, R. W., Adams, P. D., Winn, M. D., Storoni, L. C., & Read, R. J. (2007). Phaser crystallographic software. *Urn:Issn:0021-8898*, *40*(4), 658–674. https://doi.org/10.1107/S0021889807021206

Murshudov, G. N., Skubák, P., Lebedev, A. A., Pannu, N. S., Steiner, R. A., Nicholls, R. A., Winn, M. D., Long, F., & Vagin, A. A. (2011). REFMAC5 for the refinement of macromolecular crystal structures. *Urn:Issn:0907-4449*, *67*(4), 355–367. https://doi.org/10.1107/S0907444911001314
